# Understanding Multi‐Stage Charge Storage on Nanoporous Carbons in Zn‐Ion Hybrid Capacitors

**DOI:** 10.1002/adma.202502422

**Published:** 2025-05-06

**Authors:** Jiaxin Li, Kangkang Ge, Anastatios Orestis Grammenos, Pierre‐Louis Taberna, Patrice Simon, Markus Antonietti, Mateusz Odziomek

**Affiliations:** ^1^ Colloid Chemistry Department Max Planck Institute of Colloids and Interfaces Am Mühlenberg 1 14476 Potsdam Germany; ^2^ CIRIMAT, UMR CNRS 5085 Université Paul Sabatier Toulouse III 118 route de Narbonne Toulouse 31062 France

**Keywords:** charge storage, electrochemical hydrogen storage, porous carbons, Zn‐ion hybrid capacitors

## Abstract

Zn‐ion hybrid capacitors (ZIHCs) are promising high‐power energy storage devices. However, the underlying charge storage mechanisms, especially the influence of proton storage, remain poorly understood. Herein, the model porous carbons are synthesized having similar specific surface areas (SSAs) and surface chemistry but different pore sizes. They highlight the role of supermicropores and small mesopores (0.86–4 nm) enabling a high capacity of 198 mAh g^−1^ (capacitance of 446 F g^−1^), while larger mesopores (4–13 nm) significantly enhance cycling stability, exceeding 0.6 million cycles. Electrochemical studies, including EQCM analysis, reveal a 4‐stage charge‐storage process under cathodic polarization, comprising adsorption and desolvation of hydrated Zn^2+^ ions, followed by water reduction, catalyzed by Zn^2+^, and formation of H_ad_. The rising pH leads to the formation of insoluble zinc hydroxysulfate hydrates (ZHS). Depending on the pore architecture, the precipitation of ZHS has different effects on the overall stability of cycling. The study overall: (i) presents a simplified method for pore control in carbon synthesis; (ii) discuss the effect of pore size on charge storage and cycling stability in respect of ZHS formation; (iii) sheds light on the charge storage mechanism indicating the important contribution of cation effect known from electrocatalysis on faradaic charge storage mechanism.

## Graphical abstract

Porous carbons with tunable pore sizes are synthesized to investigate the impact of pore size on charge storage in Zn‐ion hybrid capacitors. An unusual multi‐stage charge storage mechanism is revealed, involving a transition from capacitive Zn^2+^ ion storage to faradaic H_ad_ adsorption on the carbon surface, providing new insights into Zn‐ion electrochemistry.

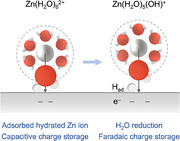

## 1 Introduction

Aqueous ZIHCs, composed of a battery‐like metallic Zn anode and a capacitive porous carbon cathode in aqueous electrolytes, are gaining notable attention for their sustainability, moisture/oxygen tolerance, and excellent electrochemical performance, including high specific power/energy and durability.^[^ ^1^, ^2^, ^3^ ^]^ These advantages primarily arise from Zn, which is an abundant and cost‐effective element. Zn anode offers a high theoretical specific capacity (820 mAh g^−1^), a low and stable redox potential (−0.76 V vs standard hydrogen electrode), and compatibility with non‐flammable, highly conductive aqueous electrolytes. On the cathode, porous carbons, with high SSAs, tunable pore sizes, and robust stability, ensure good rate capability and cycling stability.^[^ ^4^ ^]^

Despite these advantages of ZIHCs, their underlying charge storage mechanisms, specifically the processes occurring at the cathode/electrolyte interfaces, remain only partially understood. Charge storage in ZIHCs is typically attributed to a combination of faradaic Zn stripping/plating at the anode and capacitive processes at the cathode.^[^ ^1^, ^5^ ^]^ Drawing from supercapacitor studies, the cathodic process is most commonly explained using a capacitive charge storage model that encompasses electrochemical double‐layer capacitive or pseudocapacitive behaviors, i.e., the adsorption/desorption of Zn ions and anions on the carbon surface via electrostatic forces or chemical interactions.^[^ ^5^, ^6^, ^7^, ^8^, ^9^, ^10^, ^11^ ^]^ Additionally, protons from Zn ion hydrolysis (referred to as free H^+^) are also believed to contribute to charge storage,^[^ ^5^, ^6^, ^7^, ^8^, ^9^ ^]^ as indicated by increased pH at the cathode surface^[^ ^12^ ^]^ and the significantly degraded performance observed when aqueous electrolytes are replaced with aprotic electrolytes, which eliminate the influences of protons and water.^[^ ^7^, ^13^ ^]^

However, this model contradicts with experimental observations, leaving several questions unsolved. First, the extremely low concentration of free H^+^ (ca. 10^−4^ M) in mildly acidic electrolytes (pH = 3–5) makes their contribution to charge storage negligible compared to Zn ions, which are ≈10 000 times more concentrated (see analysis in, Section S1, Supporting Information).^[^ ^14^ ^]^ This contrasts previous reports emphasizing the significant role of protons in charge storage,^[^ ^6^, ^7^ ^]^ and suggests the presence of additional mechanisms. Further, it remains unclear how proton storage interacts spatiotemporally with Zn ion storage, how to compare the respective contributions of Zn ions and protons, and how zinc hydroxysulfate hydrates (ZHS) impacts overall charge storage in ZIHCs. These uncertainties point to the need for a revised model to better describe the cathodic charge storage mechanism in ZIHCs. Recently, Dai et al. proposed a “catalysis model” as an alternative to the traditional ion‐shuttle model in Zn‐ion batteries, highlighting the central role of electrochemical *OH adsorption/desorption during water dissociation, which is enhanced by interactions with Zn ions and explains the performance exceeding that of the traditional model.^[^ ^15^ ^]^ We hypothesize that water dissociation also occurs on carbon surfaces in ZIHCs.

The charge storage mechanism in ZIHCs also depends on the pore structure. Pore size has been shown to significantly impact the electrochemical properties of conventional supercapacitors.^[^ ^16^, ^17^, ^18^, ^19^, ^20^ ^]^ Smaller pores allow ions to approach the pore walls more closely, enhancing charge screening and increasing capacitance, with maximum capacitance achieved when pore size matches the dimensions of desolvated ions.^[^ ^16^, ^19^ ^]^ However, very small pores can hinder ion movement due to steric hindrance and slow desolvation, potentially causing electrolyte depletion in nanopores.^[^ ^20^ ^]^ In contrast, larger pores facilitate faster ion transport, acting as “highways” for efficient ion movement through porous networks. In comparison to supercapacitors, charge storage in ZIHC cathodes involves not only ion shuttling but also the precipitation/dissolution of solid ZHS, which adds complexity to how pore size distribution affects their overall charge storage behaviors. While some recent studies have explored the influence of pore size on ZIHC performance,^[^ ^5^, ^9^, ^12^, ^21^ ^]^ the lack of suitable model materials that can isolate pore size effects from other variables like compositions and SSAs makes the analysis challenging.

Based on our previous studies, we synthesized model porous carbons with similar SSA and surface composition but different pore size distributions for studying the charge storage and the pore size effect in ZIHCs. Detailed electrochemical measurements reveal at least four consecutive charge storage processes, including i) Zn ion adsorption and ii) dehydration, followed by iii) Zn^2+^ and potential‐promoted water reduction alongside simultaneous (iv) ZHS formation. Part of the capacitive charge stored through electrostatic interaction between (de)hydrated Zn^2^⁺ and negatively charged carbon is affected by ZHS precipitation, counterbalanced by the faradaic process of electrochemical hydrogen storage. Our findings highlight the significant contributions of Zn‐ion‐catalyzed water reduction to charge storage in ZIHCs. Water molecules in the hydration shell of Zn^2^⁺ are polarized due to its Lewis acidity, facilitating their dissociation and donation of protons, in a manner similar to the “cation effect” known in electrocatalysis.^[^ ^22^, ^23^, ^24^, ^25^ ^]^ Protons are reduced on the carbon surface to form adsorbed hydrogen atoms (H_ad_) and hydroxide ions (OH^−^). This process is analogous to the Volmer step (H_2_O + e^−^ → H_ad_ + OH^−^) in hydrogen evolution reactions.

## 2 Results and Discussion

**Figure 1:**
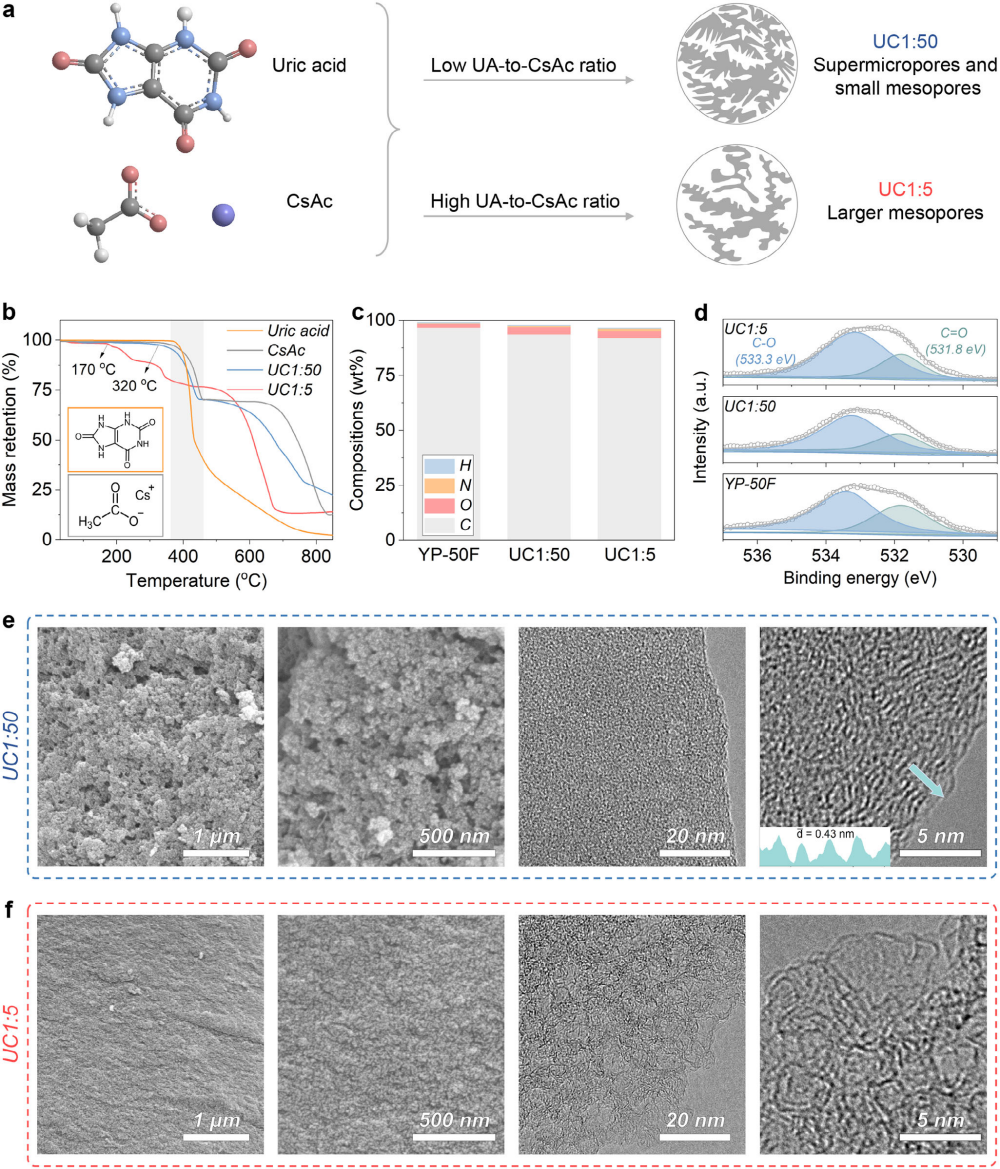
Synthesis and characterizations of porous carbons. a) Illustration of the role of uric acid to cesium acetate (UA‐to‐CsAc) ratio on pore structure. b) Thermogravimetric analysis (TGA) of UA, CsAc, and their mixtures in 1:5 and 1:50 mass ratio. c) Elemental compositions. d) High‐resolution O1s X‐ray photoelectron spectra (XPS). e,f) Scanning electron microscopy (SEM) and transmission electron microscopy (TEM) images of e) UC1:50 and f) UC1:5.

We previously developed a simple method to synthesize porous carbons with high SSAs of ca. 3000 m^2^ g^−1^ through a single‐step thermal treatment of cesium salts of carboxylic acids, such as cesium acetate (CsAc) or cesium maleate, without using harsh reagents or complex synthesis steps.^[^ ^26^, ^27^ ^]^ These porous carbons feature mostly micropores. In another set of experiments, it was found that uric acid (UA) serves as a unique precursor to produce mesoporous carbons.^[^ ^28^ ^]^ Building on these findings, we used UA as a modifier of the pore networks of CsAc‐derived porous carbons, greatly increasing mesopore contribution (**Figure** **1**a).

### 2.1 Synthesis and Characterization of Porous Carbons

The carbon materials UC1:50 and UC1:5 were prepared via direct condensation of UA and CsAc mixtures (mass ratios of 1:50 or 1:5) at 800 °C in a nitrogen atmosphere, followed by washings to remove residual Cs species. The Cs content in the resulting samples was below 0.2 wt%, as confirmed by inductively coupled plasma optical emission spectrometry. UA and CsAc have melting points of 300 and 194 °C, respectively, with their condensation occurring between 400 and 450 °C, as indicated by TGA coupled with mass spectrometry (Figures 1b; S1, Supporting Information). Notably, compared with UA or CsAc alone, their mixtures condense at even lower temperatures, specifically at 320 and 170 °C for the 1:50 and 1:5 ratios. The 1:5 mixture reaches a stable mass plateau at ≈700 °C, while other systems continue to condense/decompose even at 800 °C. The condensation at lower temperatures is likely due to weakened intermolecular interactions within UA and reduced ionic bonding within CsAc in the mixtures, favoring their melting and condensation to carbonaceous materials. It is worth noting that the carbon structures form within a homogeneous molten salt phase, as CsAc melts below 200 °C, ensuring structural homogeneity in the final products.

Elemental combustion analysis (Figure 1c) reveals that UC1:50 and UC1:5 are chemically very similar, containing ≈92 wt% of carbon, 3 wt% of oxygen, and 1 wt% of nitrogen (**Table** **1**). Their infrared spectra (Figure S2, Supporting Information) are typical for carbonaceous structures, with broad, weak absorption bands between 1200 and 1000 cm^−1^, corresponding to highly conjugated oxygen and nitrogen functionalities. XPS further supports their similar surface compositions (Figure S3, Supporting Information). In both samples, the deconvolution of C1s spectra (Figure S4, Supporting Information) identifies components centered at 288.8, 286.4, and 284.6 eV, corresponding to C═O, C─O, and sp^2^─C, bonds, respectively. The O1s peak deconvolution (Figure 1d) shows components at 533.3 and 531.8 eV, linked with C─O and C═O bonds. For comparison, commercial activated carbon YP‐50F was also included in this study. The content and type of oxygen and nitrogen functionalities stay similar across all samples, including the reference YP‐50F (Figure S5, Supporting Information), minimizing the effects of surface chemistry as a factor in ZIHCs. Additionally, X‐ray diffraction patterns (Figure S6, Supporting Information) of the samples exhibit high‐intensity sloping at angles below 10°, attributed to increased scattering from high surface area carbons.^[^ ^29^ ^]^

**Table 1:** Elemental compositions and porous structures of porous carbons.

Sample	C^a)^ [wt%]	O^a)^ [wt%]	N^a)^ [wt%]	H^a)^ [wt%]	Cs^b)^ [wt%]	V_0−0.7 nm_ ^c)^ [cm^3^ g^−1^]	V_0.7−2 nm_ ^c)^ [m^3^ g^−1^]	SSA^d)^ [m^2^ g^−1^]	V_2−50 nm_ ^e)^ [cm^3^ g^−1^]	CO_2_ uptake^f)^ [mmol g^−1^]	Max H_2_O uptake^g)^ [g g^−1^]
UC1:5	91.9	3.2	1.2	0.5	0.2	0	0.68	3200	2.90	5.0	2.2
UC1:50	92.9	3.3	0.5	0.5	0.2	0.09	1.17	3245	1.11	8.3	1.1
YP‐50F	96.6	1.7	0.4	0.6	0	0.17	0.49	1783	0.17	4.9	0.5

^a)^ Carbon, oxygen, hydrogen, and nitrogen (C/O/H/N) content were determined through elemental combustion analysis.

^b)^ Cs content was measured by ICP‐OES. Since the content of C, O, H, N, and Cs was measured using different methods, the mass ratio sum of all the above elements may not always equal 100%. Besides, incomplete combustion of carbon materials during elemental combustion analysis may have left some residue in the quartz tube.

^c)^ Micropore volumes were calculated from Ar sorption isotherms at 87 K, using the Quenched solid density functional theory (QSDFT) analysis for Ar adsorbed on carbon with slit pores.

^d)^ SSAs were evaluated at *P/P_0_* (0.05–0.30) of Ar sorption isotherms at 87 K based on the Brunauer‐Emmett‐Teller (BET) equation.

^e)^ Pore size distributions and the related volume cuts were calculated from N_2_ sorption isotherms at 77 K using QSDFT for N_2_ adsorbed on carbon with slit/cylindrical/sphere pore shape.

^f)^ CO_2_ uptake was obtained at 273 K and 100 kPa.

^g)^ The maximum water uptake capacity was obtained at 291 K and a *P/P_0_* of 0.9.

While UC1:50 and UC1:5 are chemically almost identical, they have different morphologies.SEM images show that UC1:50 forms colloidal particles merging into aerogel‐like 3D frameworks with visible voids between 50 to 200 nm (Figure 1e). This morphology is common for porous carbons obtained by salt melt synthesis, where precursors dissolve, condense into carbonaceous matter, and precipitate as spherical carbon colloids from the liquid ionic melt.^[^ ^30^ ^]^ TEM images of UC1:50 display a uniform carbon film with a combination of disordered and locally ordered structures, featuring short graphitic stacks with interlayer spacing of 0.38 to 0.45 nm. In contrast, SEM images of UC1:5 reveal a denser, void‐free microstructure (Figure 1f), as a result of intensive condensation of carbon precursors at lower temperatures than UC1:50. TEM images show a visible network of mesopores surrounded by curved and partially aligned few‐layer carbon walls. For comparison, the reference YP‐50F shows an overall disordered carbon network, yet exhibits distinct curved and partially aligned fringes of larger size than synthesized carbons,^[^ ^31^ ^]^ suggesting a higher degree of local graphitization. These observations are further supported by Raman spectra (Figure S7, Supporting Information), where UC1:5 and UC1:50 show broader D and G bands, confirming their higher structural disorder.

**Figure 2:**
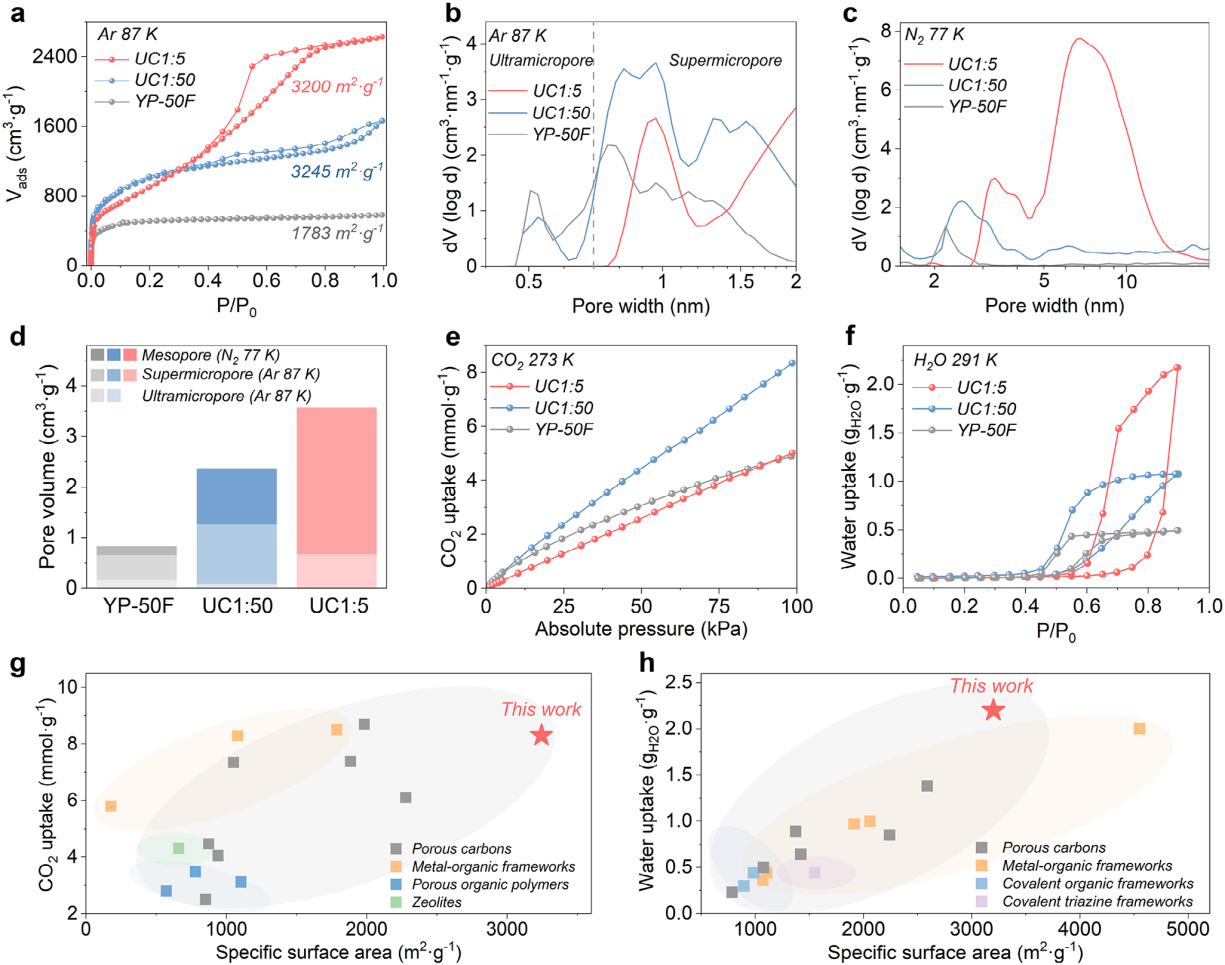
Physisorption analyses of UC1:5, UC1:50, and YP‐50F. a) Ar sorption isotherms at 87 K with high resolution at low relative pressures *P/P_0_* of 10^−6^. b,c) Pore size distributions derived from Ar and N_2_ sorption isotherms. d) Comparative overview of pore volumes. e) CO_2_ uptake isotherms at 273 K. f) H_2_O sorption isotherms at 291 K. g,h) SSAs and CO_2_/H_2_O uptake comparison with reported adsorbents. See Tables S1,S2 (Supporting Information) for additional references.

Gas sorption analysis determined the pore size distribution in the materials. The micropore size distribution was investigated by Ar and CO_2_ sorption, while the mesopores were mostly revealed by N_2_ sorption. The use of multiple techniques for pore analysis increases the reliability of determined pore size distribution. Ar and N_2_ sorption isotherms of UC1:50 (**Figure** **2**a; S8, Supporting Information) exhibit a combination of Type I(b) and Type II isotherms, with steep uptake at low relative pressure (*P/P_0_* < 0.1) typical for micropores, and a gradual increase without a clear saturation plateau near *P/P_0_* of 0.9, characteristic for macropores.^[^ ^32^, ^33^ ^]^ UC1:5 displays a Type IV(a) isotherm with a continuous increase in sorption above *P/P_0_* = 0.2 and a Type H4 hysteresis loop in the *P/P_0_* of 0.4−0.9, indicative of a large mesopores volume with a wide size distribution. The BET SSA calculated from Ar isotherm for UC1:50 (3245 m^2^ g^−1^) is practically the same as UC1:5 (3200 m^2^ g^−1^). In comparison, the reference material YP‐50F exhibits a Type I(b) isotherm, characteristic of microporous materials, with 80% of pores below 2 nm, a micropore volume of 0.66 cm^3^ g^−1^, and an SSA of 1783 m^2^ g^−1^, significantly lower than UC1:50 and UC1:5. This chemical and textural information is summarized in Table 1.

The pore size distribution was calculated using QSDFT analysis of Ar or N_2_ sorption isotherms. It reveals that UC1:50 contains micro‐ and small mesopores (0.7–4 nm), while UC1:5 predominantly features mesopores (3–13 nm, comprising 80% of total pore volume) (Figures 2b,c). These findings are consistent with TEM pictures. Ar isotherms show a higher micropore volume of UC1:50 (1.26 cm^3^ g^−1^) than UC1:5 (0.68 cm^3^ g^−1^), whereas UC1:5 has a higher mesopore volume (2.90 cm^3^ g^−1^) than UC1:50 (1.11 cm^3^ g^−1^) based on N_2_ isotherms (Figure 2d). The CO_2_ sorption analysis stays in agreement, where the highest CO_2_ uptake by UC1:50 (8.3 mmol g^−1^ at 100 kPa) supports the largest micropore volume (Figure 2e).^[^ ^34^ ^]^ Further, H_2_O sorption isotherms of all samples present Type V isotherms, with negligible uptakes at relative pressures below *P/P_0_* = 0.3 (Figure 2f), indicating the rather hydrophobic character of the carbon pore walls. The hysteresis loop is related to water condensation in mesopores. UC1:5 shows the maximum water uptake of 2.2 g_H2O_ g^−1^, consistent with its higher mesoporous volume than UC1:50 and YP‐50F. Notably, the CO_2_ uptake of UC1:50 and water uptake of UC1:5 are among the highest reported for porous carbons, metal‐organic frameworks, or polymers (Figure 2g,h; Tables S1,S2, Supporting Information), highlighting their potential as CO_2_/water adsorbents, as well as for related energy storage and conversion applications.^[^ ^26^, ^28^ ^]^ Overall, UC1:5 and UC1:50, with similar elemental compositions, SSAs, and degree of structural order but distinct pore size distributions, can be considered ideal for investigating pore size effects in ZIHCs.

### 2.2 Electrochemical Properties of Zn‐Ion Hybrid Capacitors

**Figure 3:**
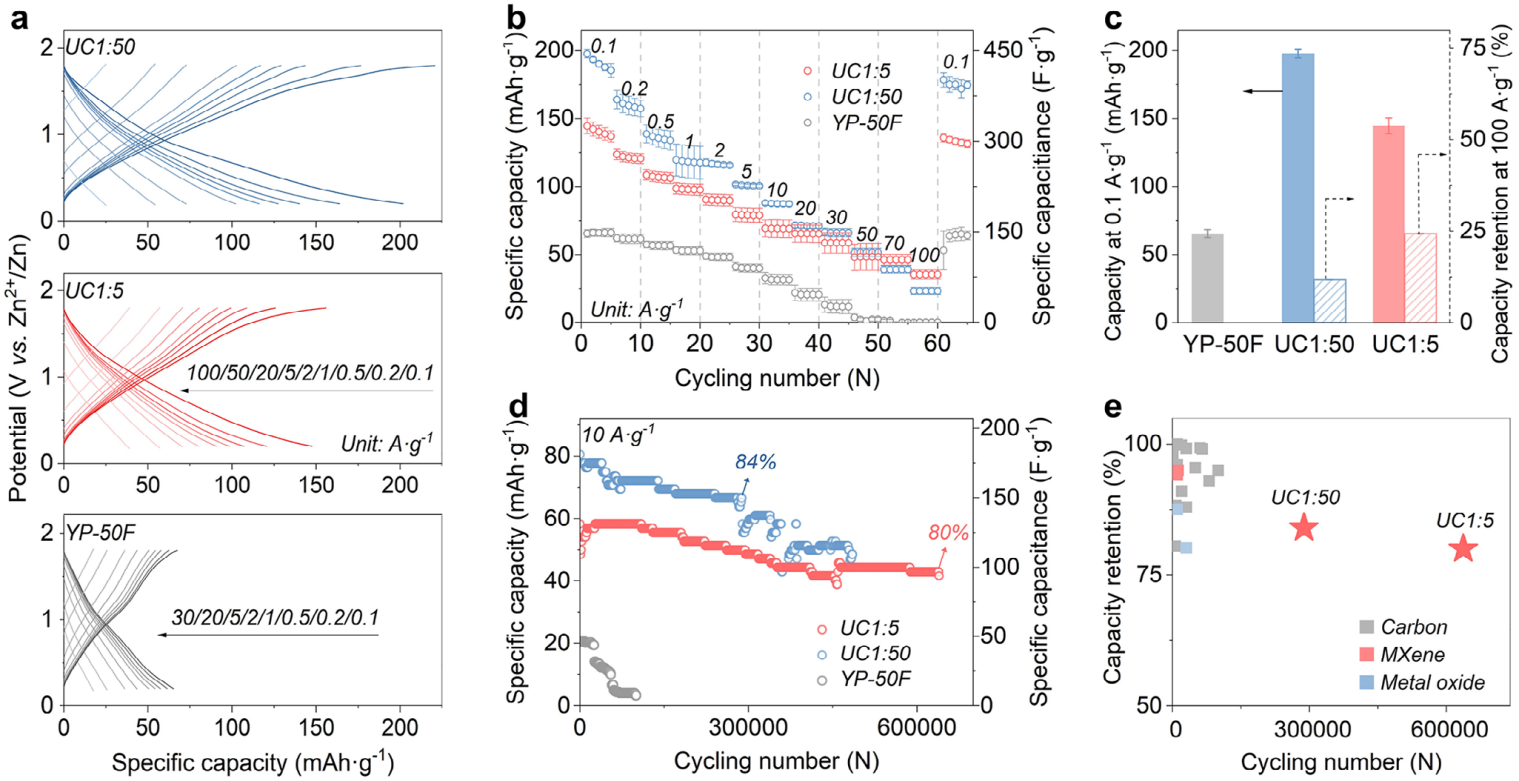
Electrochemical properties of ZIHCs using UC1:50, UC1:5, or YP‐50F as cathodes. a) Discharge–charge curves at 0.1−100 A g^−1^. b) Specific capacity (left axis) and capacitance (right axis) at 0.1–100 A g^−1^. c) Comparison of capacity at 0.1 A g^−1^ and capacity retention at 100 A g^−1^. d) Cycle performance at 10 A g^−1^. e) Capacity retention versus cycling number, comparing UC1:50, UC1:5, and reported devices. See Tables S4 and S5 (Supporting Information) for additional references.

The impact of pore size on charge storage was first examined by comparing the electrochemical properties of ZIHCs with UC1:5, UC1:50, or YP‐50F as porous cathodes. Each setup comprises a free‐standing carbon film as the cathode, Zn foil as an anode, and glass fiber separators immersed in 2 M ZnSO_4_ aqueous electrolyte (pH = 3.9) within CR2032 coin cells. The pore structures of electrode films were analyzed by Ar and N_2_ sorption (Figure S9 and Table S3, Supporting Information), confirming that there is no significant pore blockage by conductive carbon or binder. Galvanostatic charge/discharge measurements indicate that UC1:50 demonstrates discharge capacities of 198, 164, 139, and 119 mAh g^−1^ at increasing current densities of 0.1, 0.2, 0.5, and 1.0 A g^−1^, corresponding to capacitances of 446, 369, 313, and 268 F g^−1^ within a voltage window of 1.6 V (**Figure** **3**a). At higher current densities of 20, 50, and 100 A g^−1^, UC1:50 maintains capacities of 71, 48, and 23 mAh g^−1^ (160, 108, and 52 F g^−1^). In comparison, UC1:5 delivers lower capacities of 145, 124, 109, and 99 mAh g^−1^ (326, 279, 245, and 223 F g^−1^) at 0.1−1.0 A g^−1^ (Figure 3b). However, UC1:5 overtakes UC1:50 at 50 A g^−1^ and maintains a higher capacity at 100 A g^−1^ (Figure 3c). Finally, UC1:50 reveals a higher specific energy of 161.3 Wh kg^−1^ and a lower specific power of 19.2 kW kg^−1^ when compared to UC1:5 (117.2 Wh kg^−1^ and 26.2 kW kg^−1^) (Figure S10, Supporting Information). Both UC1:5 and UC1:50 outperform the reference material YP‐50F by far, which cannot deliver capacity above 30 A g^−1^ and exhibits the lowest specific energy and power of this series, being 51.7 Wh kg^−1^ and 6.2 kW kg^−1^. To reduce the impact of SSA variations between our samples and YP‐50F, normalized performances by SSAs are also provided (Figure S11, Supporting Information). Notably, YP‐50F exhibits comparable normalized capacitance to UC1:5 up to 10 A g^−1^, significantly dropping at higher currents. As a complement to gravimetric analysis, volumetric capacities/capacitances were calculated based on the total volumes of electrodes. UC1:50 and YP‐50F exhibit higher volumetric capacity/capacitance than UC1:5 at current densities below 10 mA g^−1^, as well as higher volumetric energy/power densities (Figures S12,S13, Supporting Information).

The impact of pore size on the cycling stability of the cathode was evaluated at 10 A g^−1^ (Figure 3d). UC1:50 and UC1:5 exhibit initial capacities of 81 and 56 mAh g^−1^, respectively. After 0.29 million cycles, UC1:50 still maintains 84% capacity before dropping to 55 mAh g^−1^ (68% retention). UC1:5 shows 80% capacity retention over 0.6 million cycles (≈1 year). To the best of our knowledge, this marks a durability record for porous cathodes in ZIHCs, surpassing all other porous carbons,^[^ ^6^, ^9^, ^10^, ^21^, ^35^, ^36^, ^37^ ^]^ MXenes,^[^ ^38^, ^39^, ^40^ ^]^ and metal oxides,^[^ ^41^, ^42^ ^]^ which typically demonstrate lifetimes below 100 000 cycles (Figure 3e; Table S4, Supporting Information). Just for illustration, the capacity of the reference YP‐50F declines after 20 000 cycles and ultimately fails after 100 000 cycles.

Overall, these results highlight that the carbon pore size plays a significant role in the electrochemical properties of ZIHCs. Micropores favor high capacitance, while mesopores improve rate capability and cycling stability, consistent with previous reports.^[^ ^12^, ^16^, ^43^, ^44^, ^45^ ^]^ However, excessively large mesopores can negatively impact volumetric performance. In addition, self‐discharge properties were evaluated, with UC1:5 and UC1:50 maintaining over 67% potential retention after charging to 1.8 V and resting for 500 h, while YP‐50F shows 71% retention (Figure S14, Supporting Information). This indicates the minimal impact of pore size on self‐discharge. Notably, this anti‐self‐discharge performance aligns with other reported ZIHCs and outperforms symmetrical carbon‐based supercapacitors (Figure S15 and Tables S4 and S5, Supporting Information).

### 2.3 Three‐Electrode Electrochemical Measurements

**Figure 4:**
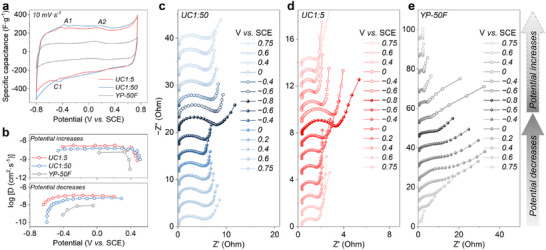
Three‐electrode electrochemical measurements for ion dynamic analysis. a) CV curves at 10 mV s^−1^. b) Ion diffusion coefficients (*D_ion_*) calculated from Galvanostatic intermittent titration technique (GITT). Electrochemical impedance spectra (EIS) Nyquist plots at different potentials of c) UC1:50, d) UC1:5, and e) YP‐50F.

To investigate the effect of pore size on ion dynamics, we used a 3‐electrode Swagelok cell setup with a carbon film as the working electrode, Zn foil as the counter electrode, and a saturated calomel electrode (SCE) as the reference electrode, all in 2 M ZnSO_4_ aqueous electrolyte. The setup ensures stable reference potential and precise monitoring of cathode potential changes.^[^ ^46^, ^47^, ^48^ ^]^ The cathode potential was measured ranging between −0.8 and 0.75 V versus SCE when operating in a ZIHC with a voltage range of 0.2 to 1.8 V versus Zn^2+^/Zn (Figure S16, Supporting Information). Cyclic voltammetry (CV) tests were conducted in the same potential range at scan rates from 5 to 200 mV s^−1^ (Figure S17, Supporting Information). At 10 mV s^−1^, both UC1:5 and UC1:50 exhibit similar quasi‐rectangular CV shapes with cathodic peaks at ≈−0.45 V (C1) and anodic peaks near −0.4 and 0.2 V versus SCE (A1, A2) (**Figure** **4**a), confirming very similar surface areas and surface chemistry. YP‐50F exhibits a smaller CV area with a visible anodic peak at 0.1 V versus SCE. These peaks are likely associated with the redox processes of surface functional groups, such as the protonation and deprotonation of carbonyl groups, accompanied by charge transfer.^[^ ^6^, ^49^ ^]^ Additionally, the anodic peaks can be related to the oxidation of adsorbed hydrogen atoms (H_ad_) from the Volmer step of water reduction, to be discussed further in the next section.

The ion dynamics related to these peaks was assessed using the relationship between peak current (*i*) and scan rate (*v*), following the equation *i = av^b^*, where *a* and *b* are adjustable parameters.^[^ ^50^ ^]^ By plotting log *i_peak_* for cathodic/anodic peaks versus log *v* from CV curves (5–40 mV s^−1^), the b‐values were determined to be close to 1 (Figure S18, Supporting Information), indicating dominant surface‐controlled processes across all samples. The diffusion rate of charge carriers within porous carbons was evaluated by the GITT.^[^ ^51^, ^52^ ^]^ UC1:5 demonstrates larger diffusion coefficients (*D_ion_*, 2 × 10^−10^−1.5 × 10^−7^ cm^2^ s^−1^) than UC1:50 (1 × 10^−10^−6 × 10^−8^ cm^2^ s^−1^) (Figure 4b and S19, Supporting Information), confirming that mesopores enable faster ion diffusion. Both synthesized samples have significantly larger diffusion coefficients than only microporous YP‐50F (2 × 10^−11^−4 × 10^−8^ cm^2^ s^−1^).

The in situ electrochemical impedance spectroscopy probed the electrochemical interfaces with the change of potential. The obtained Nyquist plots exhibit a high‐frequency loop associated with charge transfer resistance (*R_ct_*) at the electrode/electrolyte interface, and a low‐frequency slope representing Warburg impedance (*Z_w_*) from ionic diffusion within the pore networks (Figure 4c−e).^[^ ^46^, ^53^ ^]^ For UC1:50, the plots remain constant as the potential decreases from 0.75 to −0.4 V, but show enlarged loops and reduced slopes from −0.4 to −0.8 V (Figure 4c), indicating increased electronic and ionic resistance.^[^ ^53^, ^54^ ^]^ These changes are fully reversible as the potential goes back to −0.4 V and then to 0.75 V.

UC1:5 shows similar trends but with lower resistance values (Figures 4d; S20, Supporting Information), likely due to its larger mesopores, which maintain efficient mass and charge transfer while preventing pore blockage by precipitates. YP‐50F, in contrast, exhibits higher resistances between −0.4 and −0.8 V (Figure 4e), suggesting that its solely microporous architecture is severely blocked, limiting ion access. The increased electronic and ionic resistances between −0.4 and −0.8 V are associated with ZHS precipitation, confirmed by X‐ray diffraction and infrared spectroscopy (Figures S21 and S22, Supporting Information). ZHS deposits on the carbon surface as micrometer‐sized hexagonal platelets (Figure S23, Supporting Information), which block ion access to carbon pores and hamper ion transport and charge transfer. It cannot be excluded that ZHS may also form poorly crystalline, nanometer size species tightly sticking to the carbon surface. ZHS formation is also supported by Energy‐dispersive X‐ray spectroscopy (Figure S24, Supporting Information). Upon charging, ZHS dissolution at higher potentials frees these pores and surfaces, decreasing *R_ct_* and *Z_w_*.

These experiments confirm the enhanced ion dynamics associated with mesopores, a behavior commonly observed in supercapacitors.^[^ ^44^, ^45^ ^]^ However, in the case of ZIHCs, additional ZHS precipitation significantly obstructs micropores, while mesopores provide more space for its accommodation. The formation of ZHS indicates a local pH increase near the carbon electrode and thus the proton/water reduction process, contributing to the charge stored. This will be investigated further in the next section.

### 2.4 Charge Storage Mechanism Analysis

**Figure 5:**
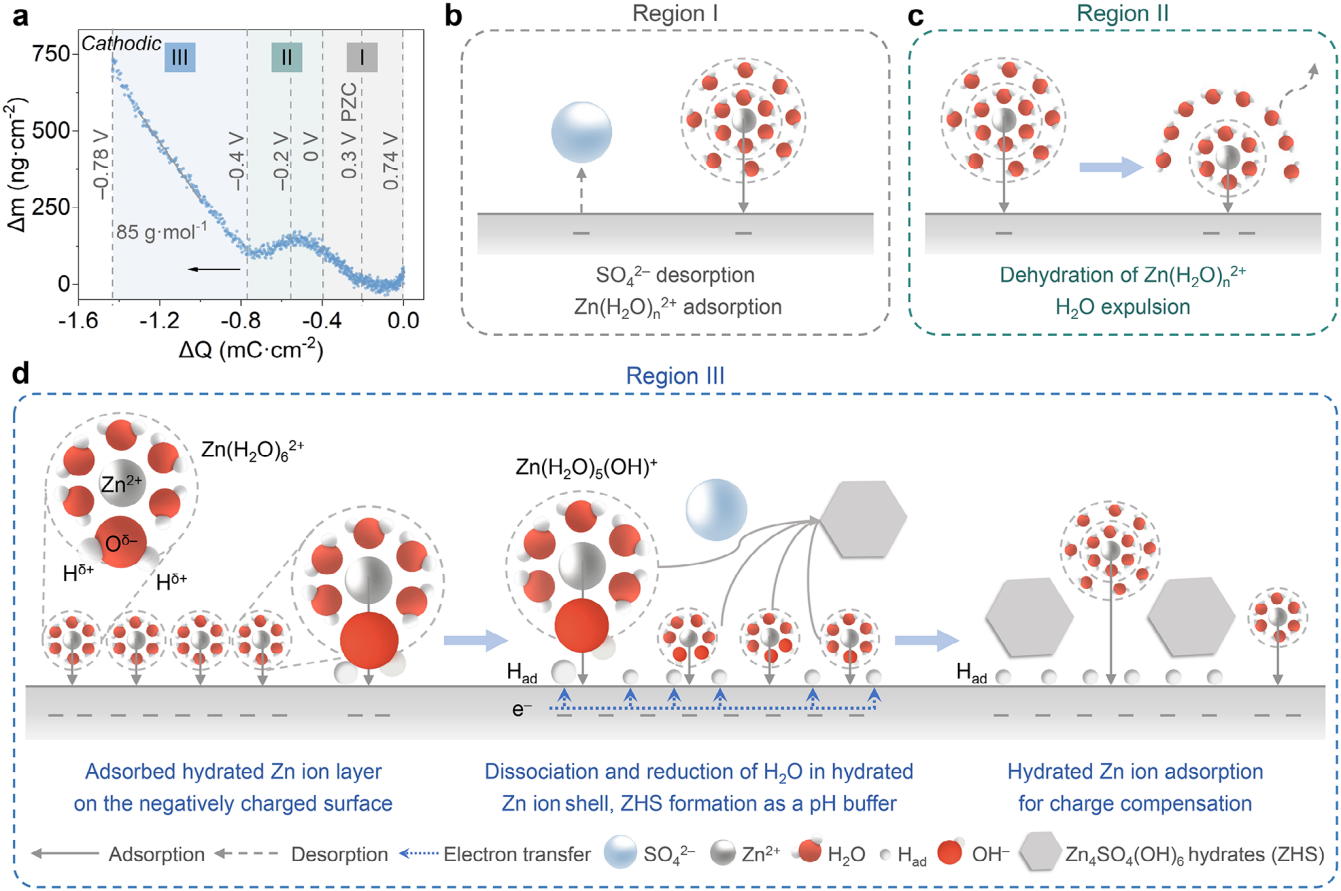
Multi‐stage charge storage on the porous carbon. a) EQCM measurements of UC1:5 coated on the gold‐quartz as a working electrode in 2 m ZnSO_4_ aqueous electrolyte, with Zn foil as counter electrode and saturated calomel electrode (SCE) as a reference electrode. Electrode mass change (*Δm*) versus accumulated charge change (*ΔQ*) during the cathodic scan of cyclic voltammetry, with the arrow representing the direction of polarization and three distinct regions shaded in different colors. Vertical grey dashed lines mark the cut‐off potentials for each region. b–d) Illustration of charge storage process in different potential regions (Region I, II, and III).

The charge storage mechanism was further investigated by the electrochemical quartz crystal microbalance (EQCM) technique. In comparison to the uncoated gold quartz electrode (Figure S25, Supporting Information), the CV area increases significantly when UC1:5 was coated on the gold‐quartz resonator (Figure S26, Supporting Information), indicating the contribution of porous carbon to charge storage. To analyze the cathodic charging mechanism, the frequency change of UC1:5‐coated gold quartz crystal (*Δf*) during the cathodic scan was converted into the mass change on the working electrode (*Δm*) using Sauerbrey's equation.^[^ ^55^ ^]^ *∆m* was plotted against the accumulated charge (*∆Q*), with 0.74 V versus SCE as the origin of the plot (*ΔQ = 0*), where the current response is zero in the CV curve (**Figure** **5**a). This helps in determining the nature of adsorbed/desorbed species on the carbon.

The potential of zero charge (PZC) for UC1:5 is ≈0.3 V versus SCE (Figure S27, Supporting Information), defining the onset of dominant Zn^2+^ adsorption during cathodic polarizations. In the potential range of 0.74–0 V vs. SCE (Region I), the CV shows typical capacitive behavior with a limited mass change on the electrode. Most likely, the charge storage in this range is a combination of anion desorption and cation adsorption.^[^ ^56^, ^57^ ^]^ During negative polarizations, SO_4_^2−^ desorption initially decreases mass above PZC, followed by adsorption of hydrated Zn^2+^ ions around PZC, which increases the corresponding mass, especially below PZC where “all” SO_4_^2−^ are desorbed (Figure 5b). Between 0 and −0.4 V (Region II), the mass peaks at −0.2 V and decreases with continued charge accumulation. This behavior is primarily linked to the partial dehydration of the outer‐sphere hydration shell of solvated Zn^2+^ ions. As charge increases, hydrated Zn^2+^ ions are brought closer to the carbon surface, allowing for tighter ion packing and more efficient charge storage (Figure 5c). During this desolvation process, free water molecules are released and exit the pores, leading to the observed mass decrease. This process aligns well with recent findings,^[^ ^58^ ^]^ where Zn^2^⁺ desolvation under cathodic polarization enhances capacitance due to strong electrostatic interactions between Zn^2^⁺ ions and carbon. The electrochemical interaction between hydrated Zn^2+^ ions and carbon surface is further supported by in situ Raman spectroscopy, showing a reversible increase in the D and G band intensities at low potentials (Figure S28, Supporting Information). This behavior aligns with previous reports attributing such changes to negative doping and metal ion‐graphene interactions,^[^ ^21^ ^]^ supporting the electrochemical adsorption of hydrated Zn ions on the carbon surface.

In the high cathodic polarization range (−0.4 to −0.8 V, Region III), the broad CV peak primarily results from the faradaic proton‐electron combination. Given the low H^+^ concentration at this pH, the process stems from water reduction and generation of OH^−^. The resultant pH increase causes ZHS precipitation on the carbon surface, as confirmed by X‐ray diffraction, infrared spectroscopy, and SEM/EDS in the previous section. The estimated average molar weight per charge in this Region is 85 g∙mol^−1^, suggesting approximately 1 proton reduction and 0.16 Zn_4_SO_4_(OH)_6_·4H_2_O formation (M_w_ = 532 g∙mol^−1^) on the carbon surface. This matches the deprotonation of 6 water molecules and the generation of 6 OH^−^ locked in ZHS (per unit of ZHS). At such low potentials, hydrated Zn^2+^ ions likely already pack tightly onto the carbon surface. The water molecules in their solvation shells are “activated” by interaction with Zn^2+^ (Lewis acid), facilitating the dissociation of this water molecules and the reduction of resulting protons onto the negatively charged carbon surface (Figure 5d). The effect of Zn^2+^ can be matched to the “cation effect”, commonly studied in electrocatalysis.^[^ ^22^, ^23^, ^24^, ^25^ ^]^ Water dissociation is concomitant with its reduction, with protons involved in a faradaic transition, combining with electrons from the carbon surface to form surface‐bound hydrogen species (H_ad_). This process corresponds to the Volmer step in the hydrogen evolution reaction.^[^ ^59^ ^]^

As protons transfer from water to the carbon surface, the resultant OH^−^ anions are liberated and bind to Zn^2^⁺ in the electric double layer, finally forming ZHS after six proton transfers per four Zn^2+^, which increases the mass on the carbon electrode (Figure 5d). This means that the system becomes both thermodynamically and kinetically stabilized. This chemical “lock‐in” process, driven by nanophase precipitation, enhances charge storage by inhibiting drastic pH fluctuations near the carbon surface, with ZHS acting as a pH buffer. However, ZHS partially covers the carbon surface, limiting further Zn^2+^ adsorption and slowing ion dynamics.

Overall, the process in Region III can be considered as an acid exchange between Zn^2+^ (Lewis acid) and H_ad_ on the carbon surface. The generated OH^−^ binds with adsorbed Zn^2+^, which reduces the positive charge and, consequently, the energy stored at the electrode/electrolyte interface. Nevertheless, the relationship between capacitive and faradaic charge storage, as well as their possible interchange, remains unclear and requires further investigation. It is worth mentioning that, as discussed by Fleischmann et al., there is a continuous transition from capacitive to faradaic charge storage mechanisms in confined electrolytes, where partial charge transfer occurs.^[^ ^60^ ^]^ Therefore, the notion of capacitive and faradaic storage might be in this case purely theoretical. In addition, we cannot exclude the process that hydrated Zn ions near the electrode surface may migrate to the electric double layer for partial charge compensation of ZHS formation at this stage (see analysis in Supporting Information, Section 2). All described cathodic processes are reversible during the anodic scan (Figure S29, Supporting Information). Similar EQCM results for UC1:50 suggest that pore size does not significantly affect the overall charge storage mechanism, but the extent to which it occurs (Figure S30, Supporting Information).

Here, it is important to note that EQCM has the limitation of only measuring changes in electrode mass, which may result from the movement of ions, solvents, or precipitates into or out of the electrode.^[^ ^61^, ^62^ ^]^ This makes it challenging to precisely identify and quantify adsorbed/desorbed species. Nevertheless, we can still summarize the overall charge storage mechanism as follows. At high potentials, desorption/adsorption of hydrated SO_4_^2−^/Zn^2+^ ions dominates, contributing to electric double‐layer capacitance. Partial dehydration of hydrated Zn ions at mid‐range potentials further increases capacitance. At lower potentials, charge storage occurs through processes involving the Volmer step (reduction of water molecule generating surface‐bound H_ad_ and OH^−^ in the electrolyte) and ZHS formation. This sequential progression, starting from Zn^2^⁺ ion adsorption, followed by partial dehydration, the Volmer reaction, and finally ZHS formation, defines the multi‐stage charge storage process. All these processes reverse during the anodic charging process.

Overall, these mechanisms give a high stored electric charge by contributing capacitive Zn^2+^ and faradaic H_ad_ parts. As these processes are all mostly local effects, they can occur at high rates and reversibly, all summing up to advantageous overall performance. Compared with previous models, which mainly focus on capacitive ion adsorption/desorption,^[^ ^5^, ^6^, ^7^, ^8^, ^9^, ^10^, ^11^ ^]^ our study confirms Zn^2^⁺ dehydration as a critical step enabling efficient ion packing and subsequent faradaic reactions. Importantly, although proton‐involved reactions were previously suggested,^[^ ^5^, ^6^, ^7^, ^8^, ^9^ ^]^ this underlying mechanism shows the role of water as the proton source in mildly acidic electrolytes.

### 2.5 Impacts of Pore Size on Charge Storage

The above mechanistic analysis shows that charge storage during the cathodic process is dominated by the adsorption of hydrated Zn ions and Zn^2+^‐catalyzed water reduction alongside simultaneous ZHS formation. In aqueous electrolytes, Zn ions are highly hydrated due to their high charge density and solvation energy, leading to an ionic size of 0.86 nm for Zn(H_2_O)_6_^2+^ in comparison to 0.15 nm for bare Zn^2^⁺.^[^ ^63^, ^64^ ^]^ The presence of an outer hydration shell, such as in Zn(H_2_O)_12.7_^2+^, further increases the size.^[^ ^58^ ^]^ Consequently, the performance variations across different pore sizes are primarily determined by the packing and dynamics of hydrated Zn ions within and across nanopores. Additionally, the accumulation of solid ZHS in different pore sizes further influences ion dynamics, which in turn impacts both rate capability and cycle stability. This process is absent in conventional supercapacitors. Based on this model, we can summarize the pore size effects as follows.

**Figure 6:**
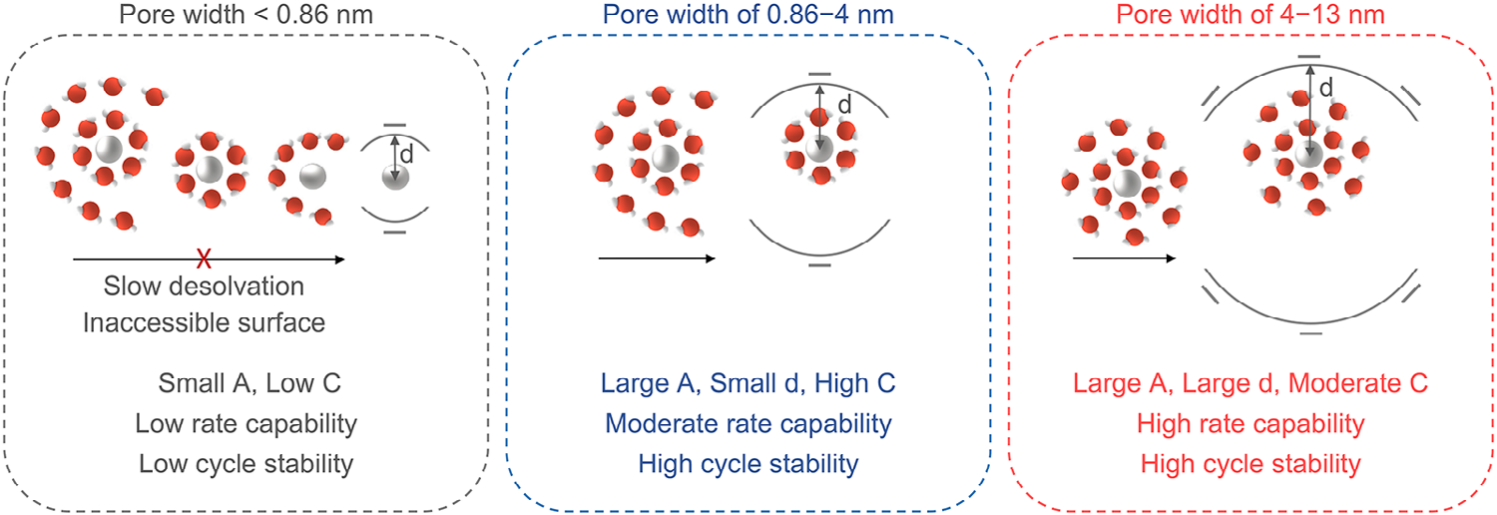
Illustration of hydrated Zn ions entering and packing in pores of increasing sizes and impacts of pore size on electrochemical properties. The capacitance C is determined by *C = εA/d*, where *A* is the accessible surface area for charge carriers, *d* is the average distance from the ionic charge center to the electrode surface, and *ε* is the electrolyte permittivity. Pore size influences electrochemical properties by affecting *A* or *d*.

At low charging rates, where mass transfer limitations are minimal, hydrated Zn ions, primarily Zn(H_2_O)_6_^2+^, can easily diffuse into and pack in the nanopores of UC1:50 (0.86–4 nm). This effective packing, aided by partial dehydration, maximizes surface utilization and brings adsorbed ionic species closer to the carbon surface, efficiently balancing the electrode charge (**Figure** **6**), and leading to higher capacitance (Figure 3b).^[^ ^58^ ^]^ In comparison, UC1:5, with larger mesopores (4–13 nm), allows entry of Zn ions with secondary hydration shells. This increases the distance between the ion charge center and carbon surface, even under partial dehydration at more negative potentials, resulting in less efficient charge screening and lower capacitance. For YP‐50F, some micropores are too small (< 0.86 nm) to accommodate Zn(H_2_O)_6_^2+^. Accessing these pores requires slow desolvation of primary hydration shells, which reduces the surface accessibility. However, Zn(H_2_O)_6_^2+^ can still enter larger micropores (0.86–2 nm), where shorter ion‐carbon distances partially compensate for the reduced surface area, leading to comparable normalized capacitances to UC1:5 (Figure S8, Supporting Information).

At higher charging rates, insufficient ion supply in small pores hinders effective charge screening at the electrolyte‐electrode interface, particularly in YP‐50F. This issue is exacerbated by the deposition of dense ZHS, which blocks ion transport and decreases the accessible surface for charge storage. Unlike conventional supercapacitors, where ion adsorption/desorption dominates, the formation of ZHS in ZIHCs introduces additional transport limitations. Mesopores (2–13 nm) thus become essential to ensure ion accessibility and charge transfer, as demonstrated by the better rate performance of UC1:5 and UC1:50 under these conditions.

The robust cycle stability of UC1:5 and UC1:50, compared with YP‐50F, is due to their larger pore sizes and higher pore volumes, which improve ion diffusion and prevent ion depletion within pores. Particularly, the well‐developed larger mesopores in UC1:5 serve as efficient ion highways, balancing mass and electron transfer, while also providing buffer space to mitigate structural stress from ion adsorption and ZHS precipitation. This prevents excessive ZHS accumulation in confined nanopores, maintains open ion pathways, and helps stabilize the system for at least 600000 cycles. In contrast, microporous YP‐50F experiences rapid capacitance decline upon cycling, likely due to pore blockage or collapse from high‐rate ion shuttling and repeated ZHS precipitation/dissolution.^[^ ^65^, ^66^, ^67^ ^]^ These issues hinder ion diffusion, reduce surface availability, and increase structural stress, ultimately compromising long‐term stability.^[^ ^65^, ^66^, ^67^ ^]^ Finally, it is worth noting that structural disorder may also influence performance.^[^ ^57^, ^68^ ^]^ For example, the higher disorder in UC1:50 may contribute to its higher normalized capacitance compared with YP‐50F. Although it is difficult to unequivocally decouple the effects of pore size and carbon defect, the pore size still seems to play the dominant role.

## 3 Conclusion

In summary, we presented a facile synthetic strategy for porous carbons, which mesopore volume can be adjusted by adding uric acid acting as pore modifier. The pore sizes are modulated while maintaining comparable large SSAs (>3000 m^2^ g^−1^) and functionalities without additional activation. The obtained carbons, categorized by their micro‐ or mesoporous character, served as model materials to study the mechanisms of charge storage in ZIHC cathodes and its pore‐size dependence. Notably, an optimized nanopore structure within the 0.86–4 nm range ensures a high capacity of 198 mAh g^−1^, while mesopores (4–13 nm) make the material resilient for up to 0.6 million cycles.

Electrochemical studies including EQCM measurements shed light on the underlying charge storage mechanisms and highlight the important contribution of electrochemical hydrogen storage facilitated by Zn^2+^ cations. Charge storage begins with the adsorption and desolvation of hydrated Zn^2+^ ions. As the proton concentration in 2 M ZnSO_4_ is too low to effectively contribute to the stored charge, water reduction, and H_ad_ generation occur, catalyzed by Zn^2+^ Lewis acid, triggering OH^−^ formation, locally increasing pH, and causing the precipitation of ZHS. The binding of OH^−^ with Zn^2+^ reduces its charge and, therefore, the energy stored. Further investigation is needed to understand how the capacitive and faradaic charge storage in this case are mutually associated. From a broader perspective, our findings underscore the significance of the cation effect known from electrocatalysis in energy storage, which can be useful in designing future sustainable energy storage devices. ZHS formation in the final state chemically locks OH^−^ and stabilizes the system at ultimate cathodic polarizations, associated with the increased capacitance of ZIHCs compared to traditional supercapacitors. On the other hand, ZHS precipitation blocks the surface and causes structural tensions in the material. Therefore, materials with highly developed surface areas and mesopores are necessary to accommodate the precipitating salt, providing higher stability.

## Conflict of Interest

The authors declare no conflict of interest.

## Supporting information



Supporting Information

## Data Availability

The data that support the findings of this study are available from the corresponding author upon reasonable request.

## References

[1] J. Yin , W. Zhang , N. A. Alhebshi , N. Salah , H. N. Alshareef , Adv. Energy Mater. 2021, 11, 2100201.

[2] L. Dong , W. Yang , W. Yang , Y. Li , W. Wu , G. Wang , J. Mater. Chem. A 2019, 7, 13810.

[3] D. Zhang , L. Li , Y. Gao , Y. Wu , J. Deng , ChemElectroChem 2021, 8, 1541.

[4] D. Sui , M. Wu , K. Shi , C. Li , J. Lang , Y. Yang , X. Zhang , X. Yan , Y. Chen , Carbon 2021, 185, 126.

[5] X. Li , C. Cai , P. Hu , B. Zhang , P. Wu , H. Fan , Z. Chen , L. Zhou , L. Mai , H. J. Fan , Adv. Mater. 2024, 36, 2400184. 10.1002/adma.202400184 38348892

[6] J. Yin , W. Zhang , W. Wang , N. A. Alhebshi , N. Salah , H. N. Alshareef , Adv. Energy Mater. 2020, 10, 2001705.

[7] H. Xu , W. He , Z. Li , J. Chi , J. Jiang , K. Huang , S. Li , G. Sun , H. Dou , X. Zhang , Adv. Funct. Mater. 2022, 32, 2111131.

[8] C. Zhao , Y. Lin , Q. Lin , Q. Liu , Y. Liu , Z. Liu , A. Ying , Energy Storage Mater. 2023, 58, 332.

[9] L. Wang , M. Peng , J. Chen , T. Hu , K. Yuan , Y. Chen , Adv. Mater. 2022, 34, 2203744. 10.1002/adma.202203744 35951671

[10] L. Wang , M. Peng , J. Chen , X. Tang , L. Li , T. Hu , K. Yuan , Y. Chen , ACS Nano 2022, 16, 2877. 35129326 10.1021/acsnano.1c09936

[11] X. Li , Y. Li , X. Zhao , F. Kang , L. Dong , Energy Storage Mater. 2022, 53, 505.

[12] F. Kang , Y. Li , Z. Zheng , X. Peng , J. Rong , L. Dong , J. Colloid Interface Sci. 2024, 669, 766. 38744154 10.1016/j.jcis.2024.05.048

[13] H. Wang , M. Wang , Y. Tang , Energy Storage Mater. 2018, 13, 1.

[14] Z. Huang , T. Wang , H. Song , X. Li , G. Liang , D. Wang , Q. Yang , Z. Chen , L. Ma , Z. Liu , B. Gao , J. Fan , C. Zhi , Angew. Chem., Int. Ed. 2021, 60, 1024. 10.1002/anie.202012202 32965789

[15] Y. Dai , R. Lu , C. Zhang , J. Li , Y. Yuan , Y. Mao , C. Ye , Z. Cai , J. Zhu , J. Li , R. Yu , L. Cui , S. Zhao , Q. An , G. He , G. I. N. Waterhouse , P. R. Shearing , Y. Ren , J. Lu , K. Amine , Z. Wang , L. Mai , Nat. Catal. 2024, 7, 776.

[16] J. Chmiola , G. Yushin , Y. Gogotsi , C. Portet , P. Simon , P. L. Taberna , Science 2006, 313, 1760. 16917025 10.1126/science.1132195

[17] J. Chmiola , C. Largeot , P. L. Taberna , P. Simon , Y. Gogotsi , Angew. Chem., Int. Ed. 2008, 47, 3392. 10.1002/anie.200704894 18366034

[18] J. A. Fernández , S. Tennison , O. Kozynchenko , F. Rubiera , F. Stoeckli , T. A. Centeno , Carbon 2009, 47, 1598.

[19] C. Largeot , C. Portet , J. Chmiola , P. L. Taberna , Y. Gogotsi , P. Simon , J. Am. Chem. Soc. 2008, 130, 2730. 18257568 10.1021/ja7106178

[20] K. Ge , H. Shao , P. L. Taberna , P. Simon , ACS Energy Lett. 2023, 8, 2738.

[21] H. Ma , H. Chen , M. Wu , F. Chi , F. Liu , J. Bai , H. Cheng , C. Li , L. Qu , Angew. Chem., Int. Ed. 2020, 59, 14541. 10.1002/anie.202005270 32506611

[22] J. T. Bender , A. S. Petersen , F. C. Østergaard , M. A. Wood , S. M. J. Heffernan , D. J. Milliron , J. Rossmeisl , J. Resasco , ACS Energy Lett. 2023, 8, 657.

[23] M. C. O. Monteiro , F. Dattila , N. López , M. T. M. Koper , J. Am. Chem. Soc. 2022, 144, 1589. 34962791 10.1021/jacs.1c10171 PMC8815072

[24] H. Khani , A. R. Puente Santiago , T. He , Angew. Chem., Int. Ed. 2023, 62, 202306103. 10.1002/anie.202306103 37490318

[25] Y. Xu , Z. Xia , W. Gao , H. Xiao , B. Xu , Nat. Catal. 2024, 7, 1120.

[26] J. Li , J. Kossmann , K. Zeng , K. Zhang , B. Wang , M. Antonietti , M. Odziomek , N. López‐Salas , Angew. Chem., Int. Ed. 2023, 62, 202217808. 10.1002/anie.202217808 37024432

[27] J. Li , Y. Xu , P. Li , A. Völkel , I. Saldaña , M. Antonietti , N. López‐Salas , M. Odziomek , Adv. Mater. 2024, 36, 2311655. 10.1002/adma.202311655 38240357

[28] J. Kossmann , R. Rothe , T. Heil , M. Antonietti , N. López‐Salas , J. Colloid Interface Sci. 2021, 602, 880. 34186464 10.1016/j.jcis.2021.06.012

[29] Y. Zhu , S. Murali , M. D. Stoller , K. J. Ganesh , W. Cai , P. J. Ferreira , A. Pirkle , R. M. Wallace , K. A. Cychosz , M. Thommes , D. Su , E. A. Stach , R. S. Ruoff , Science 2011, 332, 1537. 21566159 10.1126/science.1200770

[30] E. Lepre , S. Rat , C. Cavedon , P. H. Seeberger , B. Pieber , M. Antonietti , N. López‐Salas , Angew. Chem., Int. Ed. 2023, 62, 202211663. 10.1002/anie.202211663 PMC10107103 36303469

[31] S. Mörseburg , T. Boenke , K. Henze , K. Schutjajew , J. Kunigkeit , S. L. Benz , S. Cangaz , J. Sann , F. Hippauf , S. Dörfler , T. Abendroth , H. Althues , M. Oschatz , E. Brunner , J. Janek , S. Kaskel , Carbon 2025, 232, 119821.

[32] M. Thommes , K. Kaneko , A. V. Neimark , J. P. Olivier , F. Rodriguez‐Reinoso , J. Rouquerol , K. S. W. Sing , Pure Appl. Chem. 2015, 87, 1051.

[33] K. A. Cychosz , R. Guillet‐Nicolas , J. García‐Martínez , M. Thommes , Chem. Soc. Rev. 2017, 46, 389. 27757469 10.1039/c6cs00391e

[34] P. I. Ravikovitch , A. Vishnyakov , R. Russo , A. V. Neimark , Langmuir 2000, 16, 2311.

[35] X. Shi , J. Xie , F. Yang , F. Wang , D. Zheng , X. Cao , Y. Yu , Q. Liu , X. Lu , Angew. Chem., Int. Ed. 2022, 61, 202214773. 10.1002/anie.202214773 36300583

[36] Y. Cao , X. Tang , M. Liu , Y. Zhang , T. Yang , Z. Yang , Y. Yu , Y. Li , J. Di , Q. Li , Chem. Eng. J. 2022, 431, 133241.

[37] W. Fan , J. Ding , J. Ding , Y. Zheng , W. Song , J. Lin , C. Xiao , C. Zhong , H. Wang , W. Hu , Nano‐Micro Lett. 2021, 13, 59. 10.1007/s40820-021-00588-5 PMC8187496 34138287

[38] P. A. Maughan , N. Tapia‐Ruiz , N. Bimbo , Electrochim. Acta 2020, 341, 136061.

[39] X. Li , M. Li , Q. Yang , D. Wang , L. Ma , G. Liang , Z. Huang , B. Dong , Q. Huang , C. Zhi , Adv. Energy Mater. 2020, 10, 2001394.

[40] J. Chen , H. Chen , M. Chen , W. Zhou , Q. Tian , C. P. Wong , Chem. Eng. J. 2022, 428, 131380.

[41] L. Dong , W. Yang , W. Yang , C. Wang , Y. Li , C. Xu , S. Wan , F. He , F. Kang , G. Wang , Nano‐Micro Lett. 2019, 11, 94. 10.1007/s40820-019-0328-3 PMC7770721 34138030

[42] S. He , Z. Mo , C. Shuai , W. Liu , R. Yue , G. Liu , H. Pei , Y. Chen , N. Liu , R. Guo , Appl. Surf. Sci. 2022, 577, 151904.

[43] C. Merlet , C. Péan , B. Rotenberg , P. A. Madden , B. Daffos , P. L. Taberna , P. Simon , M. Salanne , Nat. Commun. 2013, 4, 2701. 24165568 10.1038/ncomms3701

[44] C. Carbon , Y. Korenblit , M. Rose , E. Kockrick , L. Borchardt , A. Kvit , S. Kaskel , G. Yushin , ACS Nano 2010, 4, 1337. 20180559 10.1021/nn901825y

[45] H. J. Liu , J. Wang , C. X. Wang , Y. Y. Xia , Adv. Energy Mater. 2011, 1, 1101.

[46] B. Zhang , L. Wang , Y. Zhang , X. Wang , Y. Qiao , S. G. Sun , J. Chem. Phys. 2023, 158, 054202. 36754812 10.1063/5.0139347

[47] M. Gaberšček , Nat. Commun. 2021, 12, 6513. 34764267 10.1038/s41467-021-26894-5 PMC8586247

[48] S. Wang , J. Zhang , O. Gharbi , V. Vivier , M. Gao , M. E. Orazem , Nat. Rev. Methods Prim. 2021, 1, 41.

[49] C. Qiu , L. Jiang , Y. Gao , L. Sheng , Mater. Des. 2023, 230, 111952.

[50] T. Brezesinski , J. Wang , S. H. Tolbert , B. Dunn , Nat. Mater. 2010, 9, 146. 20062048 10.1038/nmat2612

[51] Y. Zhu , C. Wang , J. Phys. Chem. C 2010, 114, 2830.

[52] Z. Song , H. Li , W. Liu , H. Zhang , J. Yan , Y. Tang , J. Huang , H. Zhang , X. Li , Adv. Mater. 2020, 32, 2001001. 10.1002/adma.202001001 32309887

[53] W. Choi , H. C. Shin , J. M. Kim , J. Y. Choi , W. S. Yoon , J. Electrochem. Sci. Technol. 2020, 11, 1.

[54] N. Ogihara , S. Kawauchi , C. Okuda , Y. Itou , Y. Takeuchi , Y. Ukyo , J. Electrochem. Soc. 2012, 159, A1034.

[55] Y. Ji , Z. W. Yin , Z. Yang , Y. P. Deng , H. Chen , C. Lin , L. Yang , K. Yang , M. Zhang , Q. Xiao , J. T. Li , Z. Chen , S. G. Sun , F. Pan , Chem. Soc. Rev. 2021, 50, 10743. 34605826 10.1039/d1cs00629k

[56] Y. C. Wu , P. L. Taberna , P. Simon , Electrochem. Commun. 2018, 93, 119.

[57] H. Yin , H. Shao , B. Daffos , P. L. Taberna , P. Simon , Electrochem. Commun. 2022, 137, 107258.

[58] K. Ge , H. Shao , E. Raymundo‐piñero , P.‐L. Taberna , P. Simon , Nat. Commun. 2024, 15, 1935. 38431624 10.1038/s41467-024-46280-1 PMC10908864

[59] X. Tian , P. Zhao , W. Sheng , Adv. Mater. 2019, 31, 1808066. 10.1002/adma.201808066 30932265

[60] S. Fleischmann , Y. Zhang , X. Wang , P. T. Cummings , J. Wu , P. Simon , Y. Gogotsi , V. Presser , V. Augustyn , Nat. Energy 2022, 7, 222.

[61] A. C. Forse , C. Merlet , J. M. Griffin , C. P. Grey , J. Am. Chem. Soc. 2016, 138, 5731. 27031622 10.1021/jacs.6b02115 PMC4865825

[62] C. J. Balhatchet , J. W. Gittins , S. Shin , K. Ge , X. Liu , S. Sharma , T. Kress , P. Taberna , P. Simon , A. Walsh , A. C. Forse , J. Am. Chem. Soc. 2024, 146, 23171. 39133641 10.1021/jacs.4c05330 PMC11345813

[63] E. R. Nightingale , J. Phys. Chem. 1959, 63, 1381.

[64] Y. Marcus , J. Chem. Soc., Faraday Trans. 1993, 89, 713.

[65] E. Marcerou , B. Daffos , P. L. Taberna , P. Simon , Electrochim. Acta 2023, 472, 143399.

[66] E. Pameté , L. Köps , F. A. Kreth , S. Pohlmann , A. Varzi , T. Brousse , A. Balducci , V. Presser , Adv. Energy Mater. 2023, 13, 2301008.

[67] M. He , K. Fic , E. Frackowiak , P. Novák , E. J. Berg , Energy Environ. Sci. 2016, 9, 623.

[68] X. Liu , D. Lyu , C. Merlet , M. J. A. Leesmith , X. Hua , Z. Xu , C. P. Grey , A. C. Forse , Science 2024, 384, 321. 38635707 10.1126/science.adn6242

