# Supplementary material for: Understanding Multi‐Stage Charge Storage on Nanoporous Carbons in Zn‐Ion Hybrid Capacitors
Source: Adv Mater. 2025 May 6;37(24):2502422. doi: 10.1002/adma.202502422 (PMC12177857; doi:10.1002/adma.202502422)
Supplement: Supplementary file 1 — Supporting Information [file ADMA-37-2502422-s001.docx]

**Supporting Information**

**Understanding Multi-Stage Charge Storage on**

**Nanoporous Carbons in Zn-Ion Hybrid Capacitors**

*Jiaxin Li,^a^ Kangkang Ge,^b^ Anastatios Orestis Grammenos,^a^ Pierre-Louis Taberna,^b^*

*Patrice Simon,^b^ Markus Antonietti,^a^ and Mateusz Odziomek^a^**

^a^ Colloid Chemistry Department, Max Planck Institute of Colloids and Interfaces, Am Mühlenberg 1, Potsdam 14476, Germany

^b^ CIRIMAT, UMR CNRS 5085, Université Paul Sabatier Toulouse III 118 route de Narbonne, Toulouse 31062, France

* E-mail: mateusz.odziomek@mpikg.mpg.de

**Experimental Section**

***Reagents.*** Cesium acetate (CsAc, Thermo Scientific, 99.5%, for analysis), uric acid (UA, Thermo scientific, 99.5%, for synthesis), hydrochloric acid (Fluka, 37 vol%), carbon black (Alfa Aesar, Super P, conductive, 99+%, metal basis), polytetrafluoroethylene dispersion (Sigma-Aldrich, 60 wt% dispersion in H_2_O), and zinc sulfate heptahydrate (ZnSO_4_·7H_2_O, Sigma-Aldrich, ACS reagent, 99%) were used as received without additional purification. Zinc foil (Zn, Alfa Aesar, 0.2 mm in thickness) was polished with diamond and Al_2_O_3_ power, followed by washing with ethanol and acetone before application. Commercial activated carbon (YP-50F) was purchased from Kuraray.

***Synthesis of porous carbons.*** UA and CsAc at mass ratios of 1:5 (2 and 10 g) or 1:50 (0.2 and 10 g) were mixed and ground in a mortar. The blend was transferred to a nickel crucible, treated at 800 ^o^C (heating rate: 1 °C·min^−1^) for 2 h under an N_2_ atmosphere (flowing rate: 3 L·min^−1^), and naturally cooled to 30 ^o^C. Then, the products were washed twice using 1 M hydrochloric acid solution, thoroughly rinsed with deionized water, and dried at 60 ^o^C for 4 h. The products obtained from the 1:5 or 1:50 mass ratios of UA and CsAc were labeled as UC1:5 and UC1:50, respectively.

***Material characterizations.*** Thermogravimetric analysis (TGA) coupled with mass spectroscopy (MS) measurements was carried out by a Netzsch TG 209 F1 Libra coupled with a Thermostar Mass spectrometer (Pfeiffer Vacuum; Asslar/Germany) operating at an ionization energy of 75 eV, under helium at a ramping rate of 2.5 °C·min^−1^ from 30 to 900 ^o^C. Data were recorded and analyzed by the Proteus (6.1.0) and Quadstar (7.03, MID modus) software package. Morphological analysis was performed by scanning electron microscopy (LEO 1550-Gemini, 3 kV), Nova NanoSEM 230 coupled with a Bruker SDD-EDS detector, and transmission electron microscopy images (JEM-2100F, acceleration voltage of 200 kV). Structural analysis was carried out using X-ray diffraction (XRD, Smartlab Studio II powder X-ray diffractometer with Cu K-α radiation and a Bruker D4 X-ray diffractometer equipped with Cu K-α radiation, λ=0.154 nm), Raman spectroscopy (Renishaw inVia system with a 514 nm laser excitation source, operated at 5% laser power), Fourier transform infrared spectroscopy (FT-IR, Nicolet iS5 FT-IR spectrometer, Thermo Fischer Scientific), and X-ray photoelectron spectroscopy (XPS, Thermo Scientific K-Alpha with sp^2^ C 1s peak at 284.6 eV as a reference). Elemental compositions were determined through elemental combustion analyses (Vario Micro) and inductively coupled plasma optical emission spectrometry (PerkinElmer ICP-OES Optima 8000). Texture analyses were carried out by N_2_ sorption at 77 K (liquid nitrogen bath), Ar sorption at 87 K (heated liquid nitrogen bath), and CO_2_ sorption at 273 K (ice-water bath) by Quantachrome Quadrasorb SI apparatus. All the samples were degassed at 150 ^o^C for 20 hours under a vacuum before the physisorption tests. Specific surface areas (SSAs) were calculated from Ar adsorption isotherms in the *P/P_0_* from 0.05 to 0.25 *via* the Brunauer-Emmett-Teller (BET) equation. Total pore volumes were obtained at *P/P_0_* ≈ 0.90. Pore size distributions were determined by applying the NONLOCAL Density Functional Theory (NLDFT) model for Ar adsorbed on carbon with slit pores, and Quenched Solid Density Functional Theory (QSDFT) model for N_2_ adsorbed on carbon with slit/cylindrical/sphere pores. The pH of the electrolyte was measured by a pH meter (Thermo Scientific Orio Versa Star Pro Benchtop).

***Device assembly.*** Firstly, 40 mg of carbon powders were mixed with 5 mg of Super P conductive carbon black, and 5.5 μL of 60 wt% polytetrafluoroethylene solution in 2 mL of ethanol. The mass ratio used for this combination was 8:1:1 (active material: carbon black: binder). The mixture was sonicated for 2 h to obtain a homogeneous dispersion. The resulting ink was then put onto a glass plate, purged with air to accelerate ethanol evaporation, and mixed by two razor blades to obtain a rubber-like consistency. The consistency was dried in an oven at 60 ^o^C overnight, and subsequently rolled into a uniform thin sheet in a piece of aluminum foil by a roll miller, punched into free-standing disks (10 mm in diameter,1.6 ± 0.2 mg·cm^−2^ in mass loading of active materials), and vacuum-dried overnight at 60 ^o^C before use. For the 2-electrode Zn-ion hybrid capacitor, a piece of carbon film was stacked with 2 pieces of glass fiber separators (Whatman GF/A), incorporating 200 μL of 2 M ZnSO_4_ aqueous solution, and paired with a Zn foil in a stainless-steel coin cell (CR 2032). For the 3-electrode Swagelok cell, a piece of carbon film was used as the working electrode, a Zn foil as the counter electrode, a saturated calomel electrode (SCE) as the reference electrode, 2 M ZnSO_4_ aqueous solution as electrolyte (2 mL), and a piece of glass fiber as the separator.

***Electrochemical measurements.*** Cyclic voltammetry (CV), Galvanostatic charge-discharge, and electrochemical impedance spectroscopy were performed using a Biologic MPG-2 Galvanostatic/potentiostat at room temperature (25 ^o^C). In the measurement of 2-electrode coin cells, a working voltage window of 0.2−1.8 V *vs.* Zn^2+^/Zn was applied to prevent parasitic reactions such as water splitting. The specific capacities (Q in mAh·g ^−1^) and capacitances (C in F·g ^−1^) were determined from Galvanostatic charge-discharge curves by the following equations: Q=I×Δt/m and C=Q/U=I∫Vdt/2(m×ΔV), where I (A), Δt (s), ∫Vdt (V·s), m (g), and ΔV (V) are discharge current, discharge time, the integral area under charge/discharge curve, the mass of active material in each cathode, and discharge voltage change excluding the internal resistance drop, respectively. The specific energy (Wh·kg^−1^) and specific power (W·kg^−1^) of devices were calculated from the equations of E_Cell_=I∫Vdt/3.6m and P_Cell_=E_Cell_×3600/Δt.

CV and electrochemical impedance spectroscopy (EIS) were conducted based on three-electrode systems using carbon film as the working electrode, Zn foil as the counter electrode, and saturated calomel electrode (SCE) as the reference electrode. The potential range in these CV tests spanned from −0.8 to +0.75 V *vs.* SCE (KCl saturated solution). This potential range remains consistent with EQCM measurements. *In situ* electrochemical impedance spectroscopy tests were performed during discharge/charge at different potentials with an AC voltage of 10 mV amplitude within a frequency range of 200 kHz to 10 mHz.

***Electrochemical studies.*** The ion dynamics were studied *via* CV, utilizing the relationship between peak current density (*i*) and scan rate (*v*) following this equation: *i=a*·*v^b^,* where a and b are constants. For analytical purposes, it can be transformed into *log(i) = blog(v) + log(a)*, where *b* is the slope of *log(i)* *vs.* *log(v)* curve. A *b* value close to 0.5 suggests a dominant diffusion process, while *b* proximity to 1 indicates a rapid capacitive response. Peaks C1, A1, and A2 were chosen as the key indicators for kinetic analyses of the Zn-ion hybrid capacitor. The diffusion kinetics of ions within the carbonaceous materials were analyzed using the Galvanostatic intermittent titration technique (GITT). GITT measurement was carried out at a current density of 1 A·g^−1^ with a constant current pulse of 10 min and an open-circuit relaxation for 3 h. The diffusion coefficients can be calculated at each step according to the following equation:

$$\text{D}_{ion}\text{=}\frac{\text{4}}{\text{π}\text{t}}\left( \frac{\text{m}\text{B}\text{V}\text{m}}{\text{M}\text{B}\text{S}} \right)^{\text{2}}\left( \frac{{\text{∆}\text{E}}_{\text{s}}}{{\text{∆}\text{E}}_{\text{t}}} \right)^{\text{2}}$$

where *t* is the duration of current pulse (s), *m_B_* is the mass loading (mg·cm^−2^), *M_B_* is the molar mass, V_m_ is the molar volume, and *S* is the electrolyte-electrode contact area (cm^2^). *ΔE_s_* is the quasi-thermodynamic equilibrium potential difference before and after the current pulse (V), while *ΔE_t_* is the potential difference (V) during a charge/discharge current pulse after eliminating the internal resistance drop. L=($\frac{\text{m}\text{B}\text{V}\text{m}}{\text{M}\text{B}\text{S}}$) is the ion diffusion length (cm). For compact electrodes in this case, it can be considered as the thickness of the electrode measured by the spiral micrometer.

***Potential of zero charge (PZC) analysis.*** PZC was confirmed by measuring the minimum capacitance from CV of a three-electrode Swagelok cell using a freestanding carbon film as a working electrode, an over capacitive counter electrode of YP-50F carbon film (*ca.* 20 mg·cm^−2^) as a counter electrode, and a SCE as a reference electrode in 1 M ZnSO_4_ aqueous solution.

***Electrochemical quartz crystal microbalance measurements (EQCM).*** For sample preparation of EQCM, Au-coated quartz crystals (basic oscillating frequency of 9 MHz, AWSensors, Spain) were homogeneously spraying coated with a suspension containing 90 wt% of active material (UC1:5 or UC1:50) and 10 wt% of carboxymethyl cellulose binder in deionized water (1 mg·mL^−1^ in solid content). The mass loading of active material was 10−30 μg·cm^−2^ (within the linear change of frequency *vs.* mass change of quartz). After spraying, the coated Au-quartz was vacuum-dried at 80 ^o^C overnight. In a 3-electrode EQCM cell (polytetrafluoroethylene holder), the coated quartz crystal served as the working electrode, SCE as the reference electrode, and polished Zn foil as the counter electrode in 2 M ZnSO_4_ aqueous solution.

All EQCM measurements were carried out using a Maxtek RQCM system combined with a Biologic potentiostat for simultaneous EQCM and electrochemical measurements. Blank experiments were performed using the blank Au-coated quartz as the working electrode in the same setup. For the EQCM results, the electrode mass change was calculated based on the Sauerbrey equation (*∆m* = −*C_f_* × *∆f*), where *∆m* (g·cm^−2^) is the mass change of the coating, *C_f_* is the sensitivity factor of the crystal (ng·cm^−2^·Hz^−1^), and *∆f* is the quartz resonance frequency (Hz). In this experiment, *C_f_* was 6.98 ng·Hz^−1^ (5.43 ng·Hz^−1^·cm^−2^, the area of the Au-coated crystal electrode of 1.28 cm^2^). For consistent results, several cycles were run before starting EQCM measurements, to obtain stable reproducible electrochemical signals.

By plotting the electrode weight change *vs.* charge (Q) during negative/positive polarization, the molar weight of adsorbed/desorbed species can be calculated according to Faraday’s law in Equation, assuming a 100% faradic efficiency: $\frac{\Delta m}{\Delta Q}=\frac{M_{W}}{nF}$ , where *Δm* represents the change in mass of the coating, *ΔQ* is the accumulated charges by integrating current with time (CV curves), *n* is the ion valence number, *F* is the Faraday constant (96485 C·mol^−1^), and *M_w_* is the average molecular weight. *Δm/ΔQ* is the slope of *Δm-ΔQ* plot, and an n value of 1 is used here to calculate the normalized molecular weight per charge. In this plot, the potential at 0.74/−0.78 V (zero current) is taken as the origin of the charge (ΔQ=0) for the cathodic/anodic scan.

***In situ Raman Experiments.*** The *in situ* Raman measurements was performed using a commercial EL-Cell ECC-Opto-Std test cell. For cell assembly, a polished Zn foil (8 mm diameter) was placed on the metal piston, followed by a separator (10 mm diameter) positioned directly on top. Subsequently, 100 μL of 2 M ZnSO₄ electrolyte was added, and a free-standing carbon film was placed on the separator. A Cu mesh (EL-Cell) served as the current collector, and an optical borosilicate window was mounted above it. The cell was then securely sealed to ensure good contact among all components. Raman measurements were carried out using a Renishaw *inVia* confocal Raman microscope equipped with a 785 nm laser (50 mW, 1200 l/mm grating). During data collection, the laser power was set to 1%. The microscope was focused onto the carbon cathode surface using a white light source and a 50× objective lens; the white light was then switched to the laser beam for spectral acquisition. Raman spectra were collected during galvanostatic discharge/charge at 0.2 A·g⁻¹, with two accumulations and an exposure time of 5 s per scan. A 3 s interval was maintained between successive measurements. Data acquisition and spectral analysis were performed using Renishaw WiRE software.

**S1: Free Proton Contribution**

The maximum charge storage from protons generated by Zn ion hydrolysis (named as free H^+^) is estimated by assuming all free H⁺ contribute to charge storage.

1. Coin cell:

- Electrolyte: 200 μL of 2 M ZnSO_4_ (pH=3.9)
- Concentration of free H^+^: 10^−3.9^ M
- Mass loading of porous carbon: ~1.3 mg per electrode

1. Total charge of free H⁺:

- Moles of free H⁺ = 10^−3.9^ M×200×10^−6^ L = 2.5178×10^−8^ mol
- Total charge (Q) = 2.5178×10^−8^ mol×96485 C·mol^−1^ = 0.002427 C
- Specific capacity (C·g^−1^) = 0.002427 C/0.0013 g ≈ 1.87 C·g^−1^
- Specific capacity (mAh·g^−1^) = 1.87 C·g^−1^×1000 mA/3600 s ≈ 0.52 mAh·g^−1^

The calculated specific capacity of 0.52 mAh·g^−1^ is negligible compared to the capacities of 150−200 mAh·g^−1^ obtained for UC1:5 and UC1:50, indicating that the contribution of free H^+^ to charge storage is minimal.

Assuming that free H^+^ adsorption is charge balanced by the removal of an equivalent amount of OH^−^ from the system, which subsequently reacts to form Zn(OH)_2_ or Zn_4_SO_4_(OH)_6_·4H_2_O (ZHS), the maximum amount of Zn(OH)_2_ or (ZHS) generated from free H^+^ can be calculated as follows.

- Moles of OH^−^ = Moles of H⁺ = 2.5178×10^−8^ mol
- Moles of Zn(OH)_2_ = Moles of OH^−^/2 = 1.2589×10^−8^ mol
- Mass of Zn(OH)_2_ = 7.9245×10^−9^ mol × 99.4 g·mol^−1^ = 1.25 μg
- Moles of ZHS = Moles of OH^−^/6 = 4.1963×10^−9^ mol
- Mass of ZHS = 4.1963×10^−9^ mol×532 g·mol^−1^ = 2.23 μg

Overall, the maximum amount of Zn(OH)_2_ or ZHS from free H^+^ can only bring minor mass changes (0.1−0.2 wt%) to the carbon electrode, insufficient to generate distinct X-ray diffraction peaks. In our experiments, the carbon electrodes show a mass increase exceeding 50% after discharging to 0.2 V *vs.* Zn^2+^/Zn (after washing 3 times with deionized water to remove adsorbed ion species and drying at 60 ^o^C in vacuum), far surpassing than this estimation. This indicates that Zn-ion-catalyzed water reduction process plays a crucial role in the discharge process.

**Section 2: Charge Storage Analysis from EQCM & Discharge Curve**

To understand the electrochemical and chemical processes on the carbon surface at a microscopic level, we calculate the ion areal densities based on key assumptions and experimental data.

1. **Initial Assumptions**
2. During the cathodic polarizations of carbon electrodes (cathodic scan in 3-electrode CV measurements or discharge in 2-electrode set-ups, where negative charge accumulates on the carbon surfaces), Zn(H_2_O)_6_^2+^ ions act as the primary charge carriers at high and middle potentials, forming a homogeneously arranged single layer on the micropore surface. For simplicity, the contribution of SO_4_^2−^ desorption to charge storage at high potentials is ignored.
3. As the carbon surface becomes saturated with Zn(H_2_O)_6_^2+^ during negative charge accumulation, water molecules in the solvation shells of Zn(H_2_O)_6_^2+^ dissociate, releasing protons that combine with electrons on the carbon walls. Simultaneously, the formed OH^−^ ions bind to Zn^2+^ and SO_4_^2−^, resulting in Zn_4_(OH)_6_SO_4_ hydrates (ZHS) precipitation.
4. During the transition from capacitive Zn-ion adsorption to Faradaic proton adsorption, we do not quantify the energy change and focus only on charge storage. When 6 H_ad_ (6 positive charges) replaces 4 Zn(H₂O)₆²⁺ (8 positive charges) on the carbon surface to form ZHS, there is a net loss of 2 charges. To maintain charge balance, at least 1 additional Zn(H₂O)₆²⁺ ion (2 positive charges) must migrate from the electrolyte reservoir (namely ions in the near-surface or bulk electrolyte that do not participate in the charge storage) to the double layer. Further, to achieve net charge accumulation from water reduction (deprotonation), 5 Zn(H₂O)₆²⁺ ions must migrate from the electrolyte reservoir for charge compensation on the carbon electrode. In other words, for net charge accumulation from proton storage, Zn(H₂O)₆²⁺ ions equivalent to 5/6 of the H_ad_ amount must migrate from the electrolyte reservoir to the carbon surface for charge compensation in ZHS formation.
5. **Key Parameters**

Several parameters are presented based on our experiment results or literature.

1. BET SSA of UC1:5 from Ar sorption: 3200 m^2^·g^−1^;

Micropore SSA of UC1:5: 1062 m^2^·g^−1^;

Oxygen content of UC1:5: ~3 wt%

Note: The micropore surface area measured by Ar sorption does not accurately represent the surface area accessible to electrolyte ions during electrochemical processes. The actual accessible area is much smaller due to factors such as the size difference between Ar molecule (0.34 nm) and electrolyte ions (*e.g.,* 0.86 nm for Zn(H_2_O)_6_^2+^), incomplete wetting of carbon surface due to its hydrophobicity, and electrolyte depletion within pores at high discharge/charge rates. As a result, using BET SSAs may underestimate ion density on the carbon surface. Nevertheless, for simplicity and an approximate chemical representation, BET SSA is used for ion density calculations in the following analysis.

1. Based on EQCM analysis, in the potential range of 0.74 to −0.4 V *vs.* SCE (Region I and II), Zn(H_2_O)_6_^2+^ primarily contributes to charge storage, with a total charge accumulation of 0.765 mC·cm^−2^. From −0.4 to −0.8 V *vs.* SCE (Region III), protons primarily contribute to charge storage, with 0.653 mC·cm^−2^ of charge accumulated. This is primarily associated with UC1:5 carbon on the quartz (mass loading: ~15 μg·cm^−2^).
2. According to Galvanostatic discharge curve at 0.1 A·g^−1^_Carbon_ for UC1:5 (as shown below), a specific capacity of 85 mAh·g^−1^ from 1.8 to 0.6 V *vs.* Zn^2+^/Zn is linked to Zn(H_2_O)_6_^2+^ adsorption, while a specific capacity of 60 mAh·g^−1^ from 0.6 to 0.2 V *vs.* Zn^2+^/Zn is attributed to proton storage.


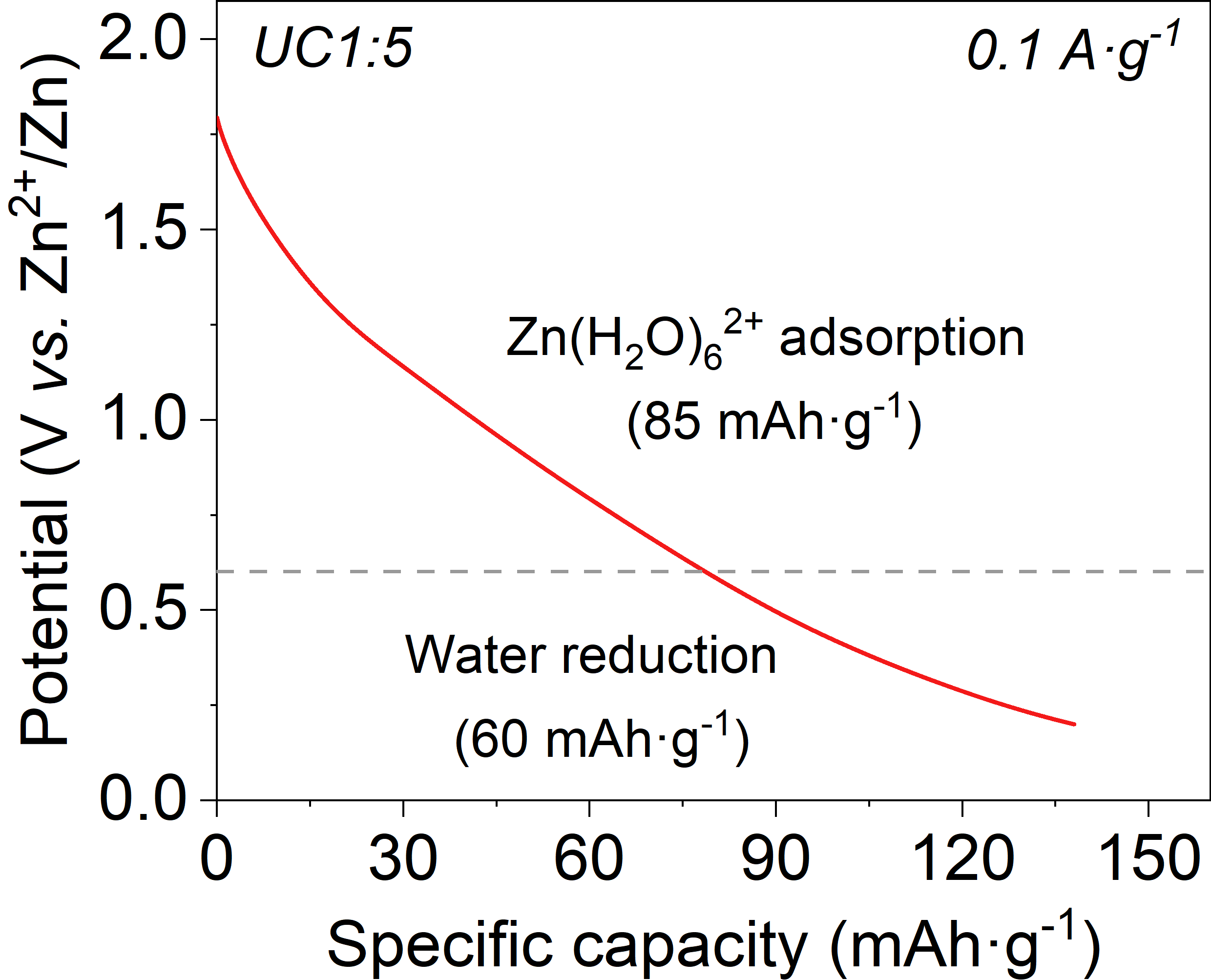


1. **Estimations based on EQCM Analysis**

To convert the accumulated charge to the number of Zn(H₂O)₆²⁺ and H_ad_ and then calculate their areal densities:

1. Moles of Zn(H₂O)₆²⁺ = 0.765 mC×10^−3^/(2×96485 C/mol) ≈ 3.97×10^−9^ mol
2. Number of Zn(H₂O)₆²⁺ = n×*N_A_*​ =3.97×10^−9^ mol×6.022×10^23^ ions/mol = 2.39×10^15^ ions
3. Moles of H_ad_ = 0.653 mC×10^−3^/(96485 C/mol) ≈ 6.77×10^−9^ mol
4. Number of H_ad_ = n×*N_A_*​ = 6.77×10^−9^ mol×6.022×10^23^ ions/mol = 4.08×10^15^ ions

In Region III, 6.77×10^−9^ mol of H_ad_ requires 5.64×10^−9^ mol of Zn(H₂O)₆²⁺ from the electrolyte reservoir to migrate to the carbon surface for ZHS formation and charge balance.

To sum up, if we consider that, besides Zn(H₂O)₆²⁺ ions in Region I and II (3.97 ×10^−9^ mol), the additional Zn(H₂O)₆²⁺ ions migrating from the electrolyte reservoir to the surface (for ZHS formation and charge storage) also contribute to charge storage in Region III (5.64×10^−9^ mol), we can roughly estimate the contribution ratio between Zn(H₂O)₆²⁺ and H_ad_ to charge storage across the whole cathodic scan as follows.

1. Charge contribution from Zn(H₂O)₆²⁺ = (3.97+5.64)×10^−9^×2×96485 C/mol=1.854 mC
2. Charge contribution from H_ad_ = 0.653 mC
3. Contribution ratio between Zn(H₂O)₆²⁺ and H_ad_ = 2.839:1 ≈ 74%: 26%

This calculation indicates that Zn(H₂O)₆²⁺ ions contribute approximately 74% of the total charge storage, while H_ad_ contributes around 26%.

Further, we could estimate the ion areal density on the carbon surface as follows:

1. Micropore surface area of UC1:5 = 1062 m^2^/g×15×10^−6^ g×10^18^ nm^2^/m^2^ = 1.593×10^16^ nm^2^
2. Areal density of Zn(H₂O)₆²⁺ = Number of Zn(H₂O)₆²⁺​/Micropore surface area ≈ 0.15 ion/nm^2^
3. Areal density of H_ad_ = Number of H_ad_ /Micropore surface area ≈ 0.25 ion/nm^2^

Given that 6 protons are required to form 1 Zn hydroxide sulfate (ZHS), the moles of ZHS formed can be calculated as:

1. Moles of ZHS = Moles of H_ad_​/6 = 6.77×10^−9^/6 mol = 1.13×10^−9^ mol
2. Mass of ZHS = 1.13×10^−9^×532 g ≈ 6 ×10^−7^ g
3. Mass of ZHS/Mass of carbon = 6×10^−7^/(15×10^−6^) ​×100% ≈ 4%

This indicates that ZHS formation brings a 4% mass increase on the carbon electrode.

The results suggest that Zn(H₂O)₆²⁺ and H_ad_ are well-spaced, with substantial space between ions to prevent overcrowding, and that ZHS formation only marginally increases the electrode mass. However, during EQCM measurements at a high scan rate of 50 mV·s^−1^ (31 s per scan), micropores may not be fully accessible to electrolyte ions, resulting in a significantly lower effective surface area than the BET SSA and an underestimation of local ion areal densities. To address this, ion areal densities were also recalculated based on charge storage from the Galvanostatic discharge curve at 0.1 A/g (5220 s per scan) in the following section. At this lower discharge rate, micropores are more accessible to electrolyte ions, making these calculations more representative of actual conditions.

1. **Estimations based on Galvanostatic Discharge Curve**

For 1 g of UC1:5 in the potential range of 1.8 to 0.6 V *vs.* Zn^2+^/Zn, the discharge capacity (85 mAh) can be converted into charge, and then the number of Zn(H₂O)₆²⁺ ions:

1. Charge = 85 mAh×3.6 C/mAh = 306 C
2. Moles of Zn(H₂O)₆²⁺ = 306 C/(2×96485 C/mol) ≈ 0.00159 mol
3. Number of Zn(H₂O)₆²⁺ = n×*N_A_* ​= 0.00159 mol×6.022×10^23^ ions/mol = 9.57×10^20^ ions
4. Micropore surface area of UC1:5 = 1062 m^2^ = 1.062×10^21^ nm^2^
5. Areal density of Zn(H₂O)₆²⁺ on UC1:5 ≈ 9.57×10²⁰/1.062×10²¹ nm² = 0.9 ion/nm^2^

This indicates that the micropore surface of UC1:5 is densely populated by Zn(H_2_O)_6_^2+^ ions (0.86 nm in diameter) after discharging to 0.6 V *vs.* Zn^2+^/Zn.

At lower potentials (0.6 to 0.2 V *vs.* Zn^2+^/Zn), H^+^ becomes the dominant charge carrier, and the discharge capacity (60 mAh) is converted to charge, and the number of H_ad_. The areal density of H_ad_ is determined as follows:

1. Charge = 60 mAh×3.6 C/mAh = 216 C
2. Moles of H_ad_ = 216 C/(96485 C/mol) ≈ 0.00224 mol
3. Number of H_ad_ = n×*N_A_*​ = 0.00224 mol×6.022×10^23^ ions/mol = 1.35×10^21^ ions
4. Areal density of H_ad_ = Number of H_ad_​/Micropore surface area ≈ 1.27 ions/nm^2^

This means that each H_ad_ occupies an area of 0.76 nm², which is reasonable given the small size of H⁺ (0.06 nm).

At low potentials, 0.00224 mol of H_ad_ would require 0.00187 mol of Zn(H₂O)₆²⁺ from the electrolyte reservoir to migrate to the carbon surface for ZHS formation and charge balance.

1. Charge storage from Zn(H₂O)₆²⁺ = (0.00159+0.00187 mol)×2×96485 C/mol = 667.67 C
2. Charge storage from H_ad_ = 216 C
3. Contribution ratio between Zn(H₂O)₆²⁺ and H_ad_ ≈ 76%: 24%

Since 6 protons are required to form 1 ZHS, the moles of ZHS formed can be calculated as follows:

1. Moles of ZHS formed = Moles of H_ad_​/6 = 0.00074667 mol
2. Mass of ZHS = 0.00074667×532 g ≈ 0.397 g
3. Mass of ZHS/Mass of carbon = 0.397/1 ​×100% ≈ **39.7%**

This indicates that ZHS formation results in a 39.4% mass increase in the carbon electrode, aligning closely with our experimental observations.

Additionally, 1 g UC1:5 contains approximately 0.03 g of oxygen (3 wt%).

Moles of oxygen = 0.03 g/16 g/mol = 0.001875 mol

Assuming that UC1:5 carbon contains only carbonyl groups, 1 g UC1:5 has 0.001875 mol of carbonyl groups, much lower than the total amount of Zn(H₂O)₆²⁺ and H_ad_ of 0.00383 mol. Therefore, we speculate that Zn(H₂O)₆²⁺ and H_ad_ interact with the carbon surface by becoming chemically or electro-statically adsorbing on the carbon atoms or by binding with surface functional groups.


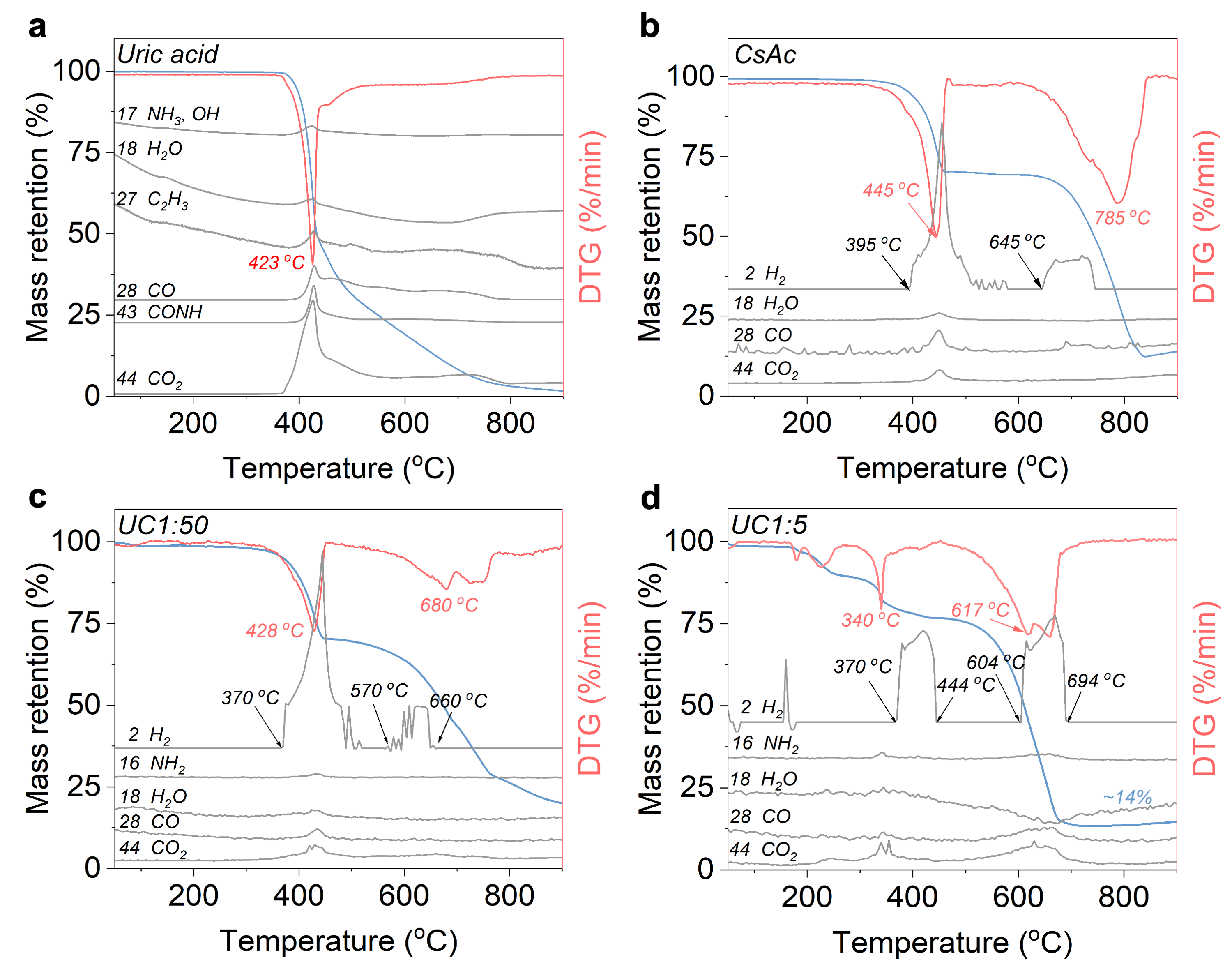


**Figure S1.** Thermogravimetric analysis coupled with mass spectra of (a) UA, (b) CsAc, (c) 1:50, and (d) 1:5 mass ratio mixtures of UA and CsAc.


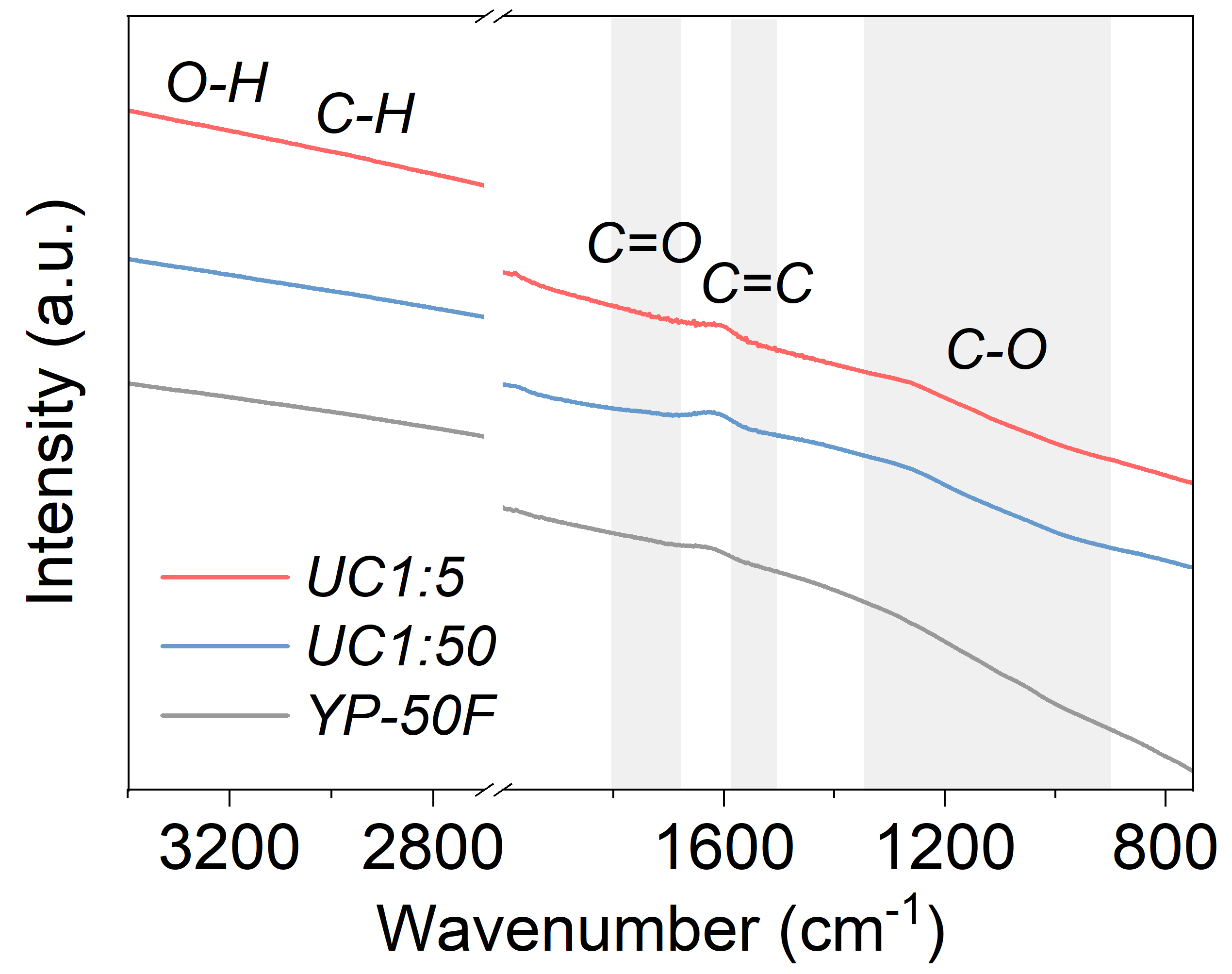


***Figure S2.*** Fourier transform-infrared spectra of UC1:5, UC1:50, and YP-50F.

The invisible band at 1600−1500 cm^−1^ originating from C=C stretching indicates the presence of large, conjugated domains. The low-intensity broad adsorption bands at 1800−1600 cm^−1^ and 1200−1000 cm^−1^ are characteristic of highly conjugated oxygen and nitrogen functionalities in the carbon networks.


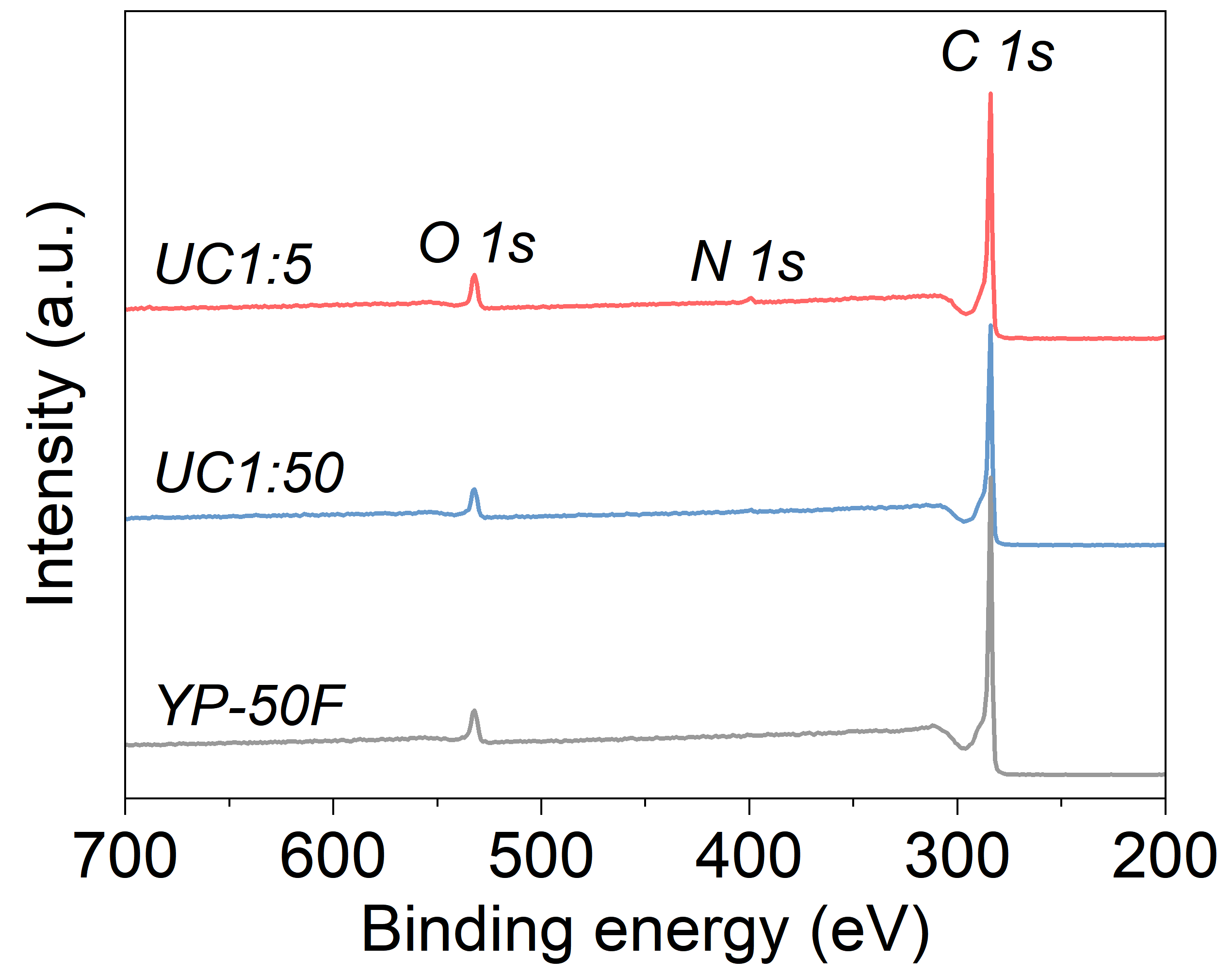


***Figure S3.*** X-ray photoelectron survey spectra of UC1:5, UC1:50, and YP-50F.


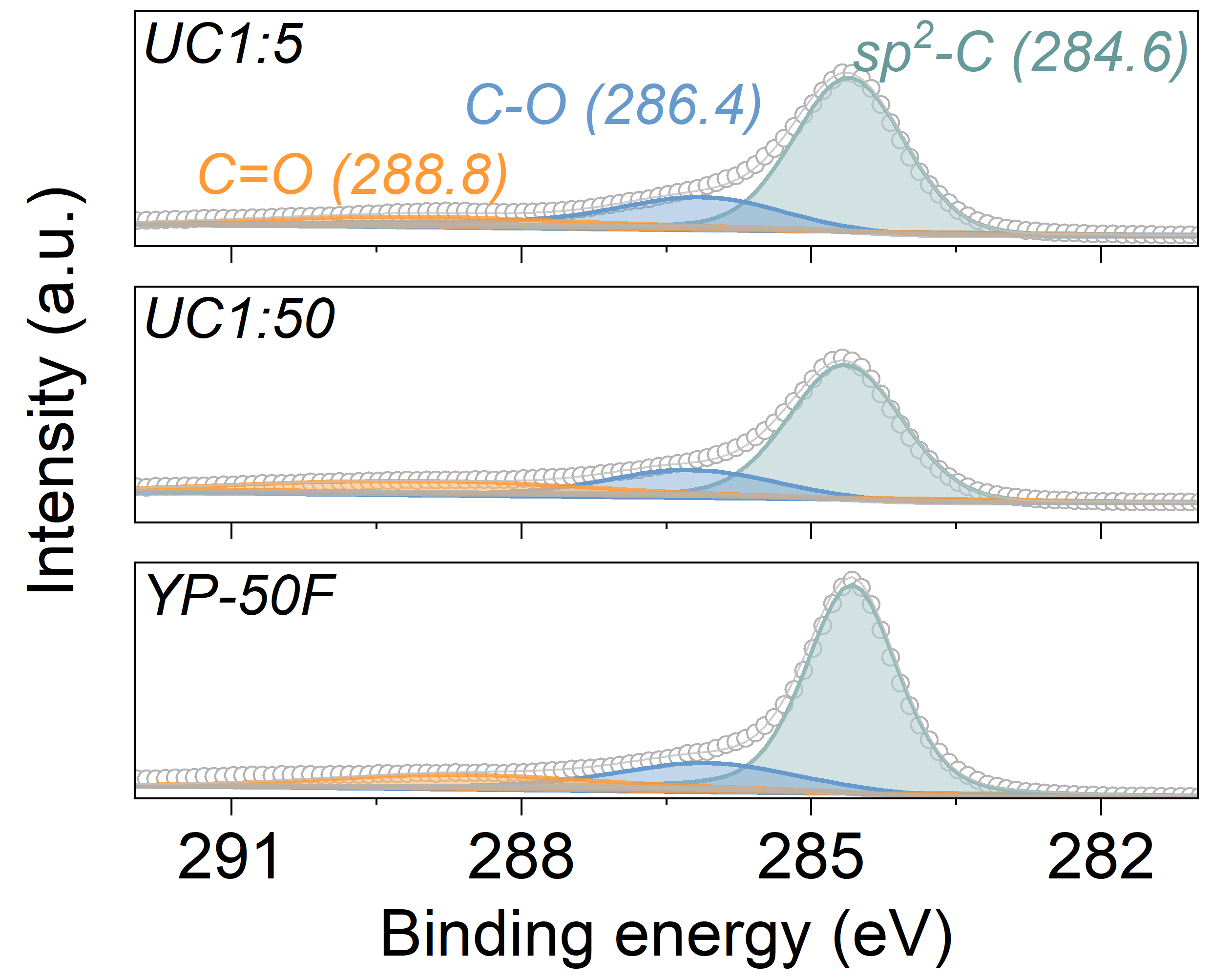


***Figure S4.*** High-resolution C1s X-ray photoelectron spectra of UC1:5, UC1:50, and YP-50F.

Deconvolution of C1s peak gives three main components centered at 284.6, 286.4, and 288.8 eV, corresponding to sp^2^-C, C-O, and C=O bonds (that can come from both the material itself and adventitious carbon), respectively.


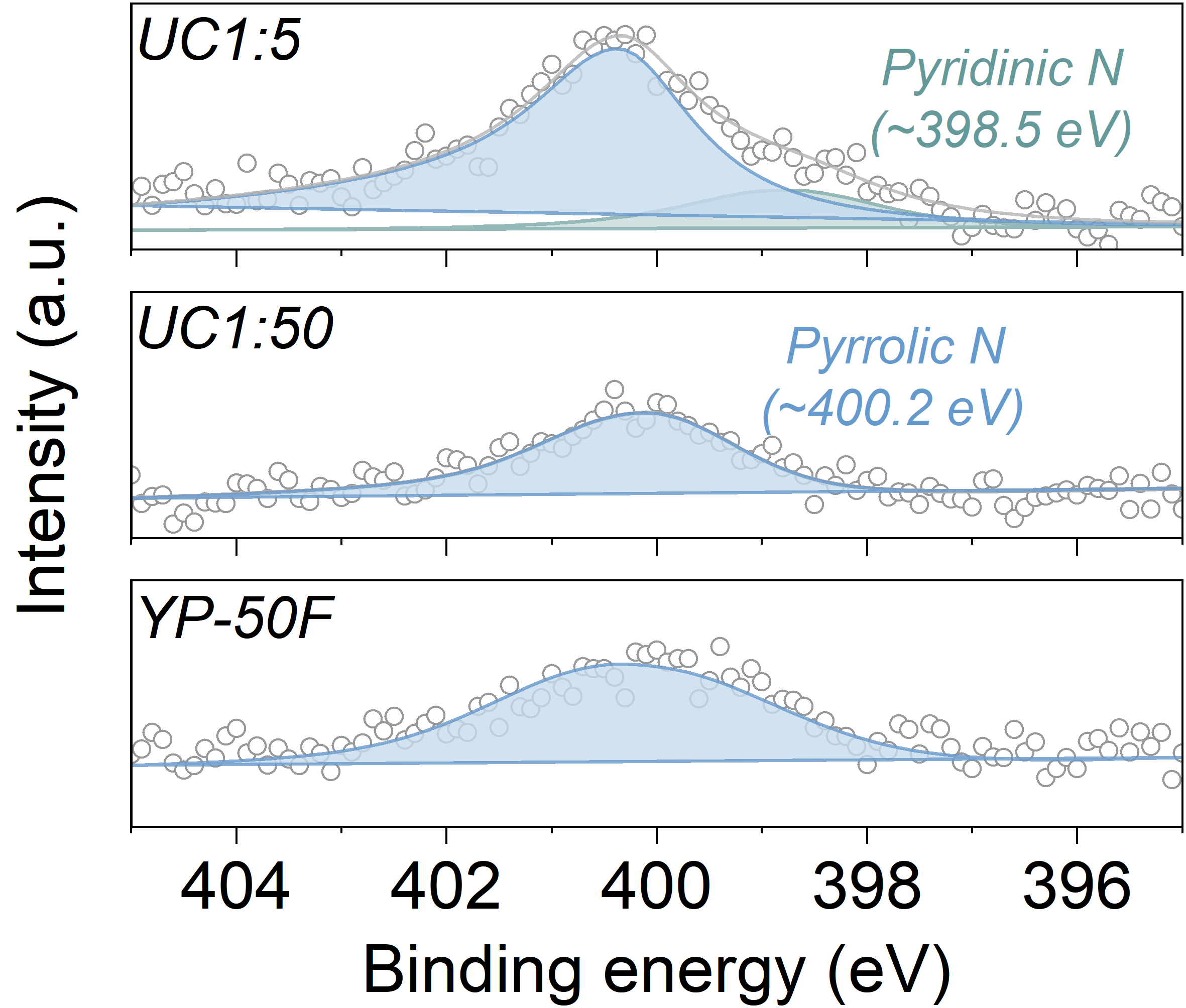


***Figure S5.*** High-resolution N1s X-ray photoelectron spectra of UC1:5, UC1:50, and YP-50F.

**
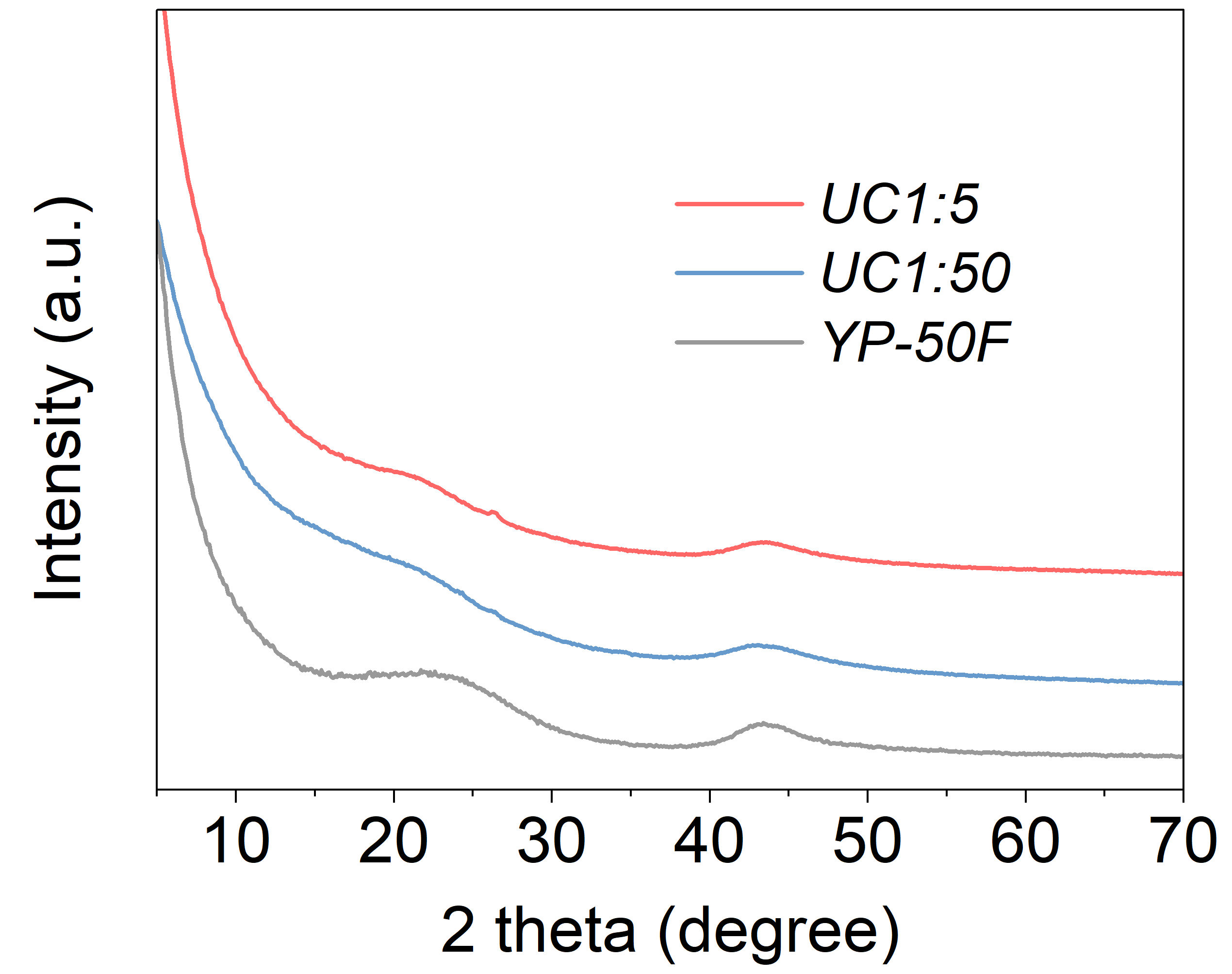
**

**Figure S6.** X-ray diffraction patterns of UC1:5, UC1:50, and YP-50F.

UC1:5 and UC1:50 display strong sloping backgrounds at low angles (<10°), which is likely due to their high porosity and amorphous nature, and may partially obscure the true position and width of their (002) peaks. In contrast, YP-50F shows a more pronounced (002) peak centered at a higher 2θ value (~24°), suggesting a relatively higher stacking order and reduced interlayer spacing compared to UC1:5 and UC1:50.


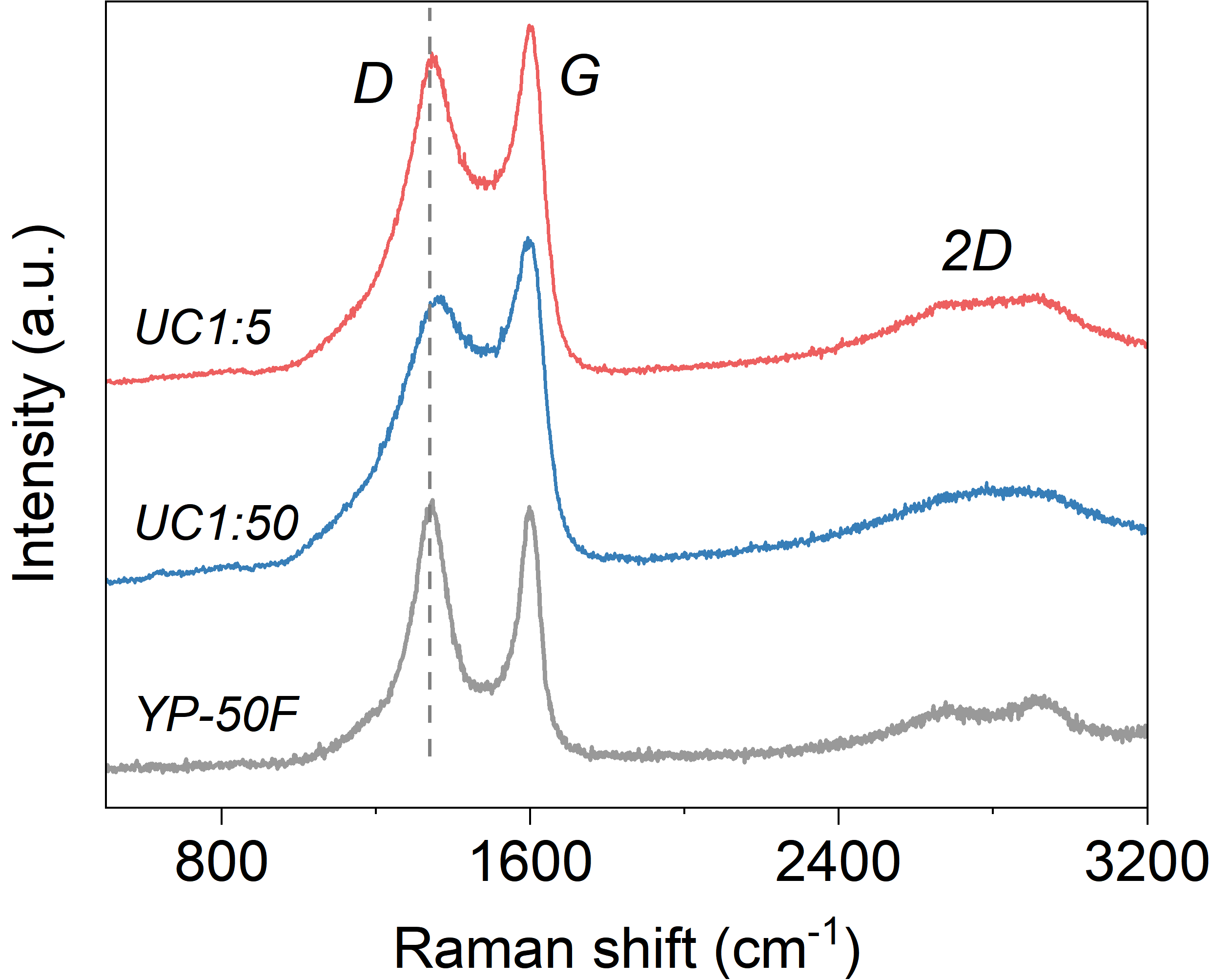


**Figure S7.** Raman spectra of UC1:5, UC1:50, and YP-50F.

Raman spectra of UC1:5 and UC1:50 show broader and more asymmetric D and G bands compared to YP-50F, indicating a higher degree of structural disorder and more varied sp² environments. The 2D band (~2700 cm⁻¹) in YP-50F is sharper, suggesting more ordered graphitic stacking. In contrast, the broader and less defined 2D bands in UC1:5 and UC1:50 indicate turbostratic structures with disordered or few-layer carbon stacking. Additionally, the D band of UC1:50 shifts to a higher wavenumber than those of UC1:5 and YP-50F, reflecting its slightly increased structural disorder in its carbon framework.^[1]^


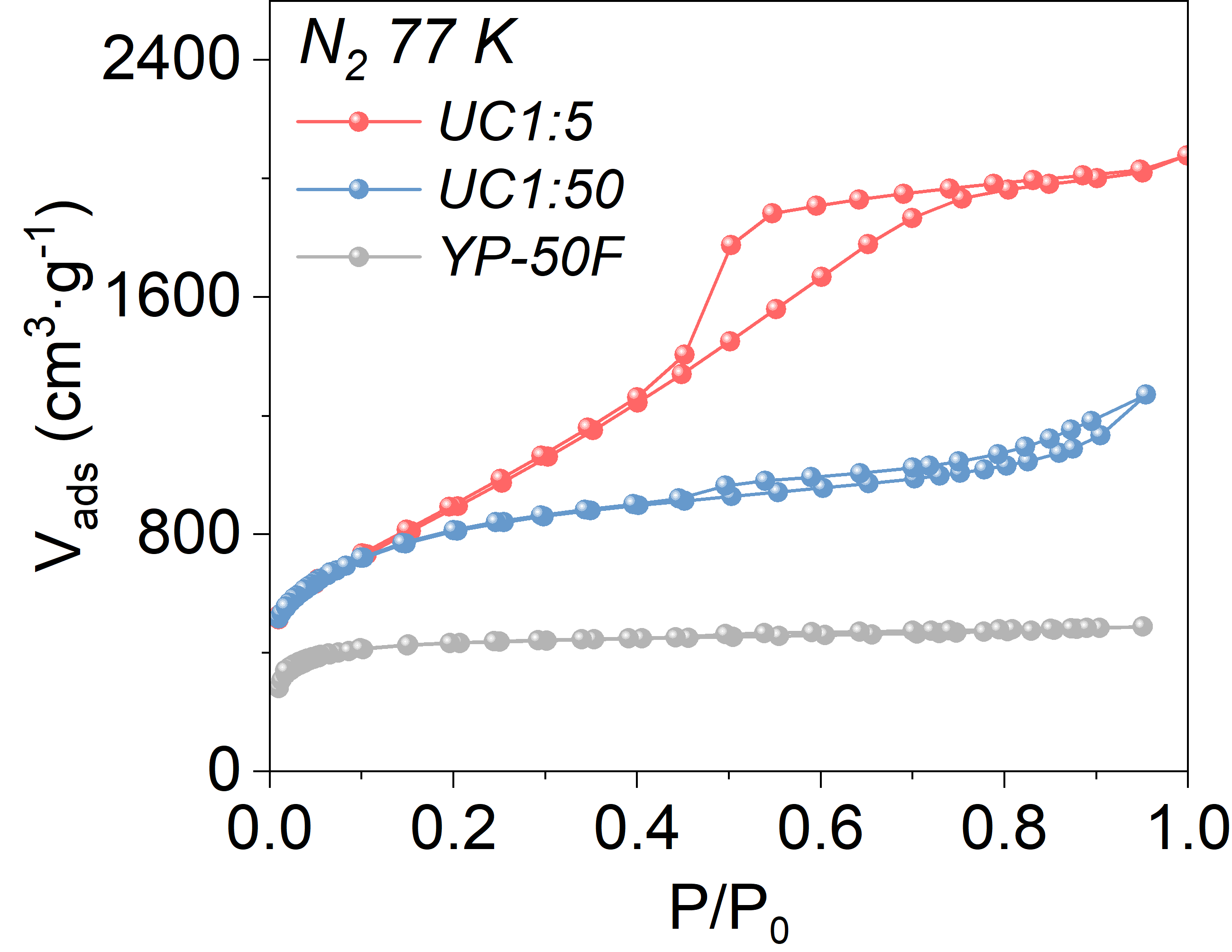


***Figure S8.*** N_2_ sorption isotherms at 77 K of UC1:5, UC1:50, and YP-50F powder.


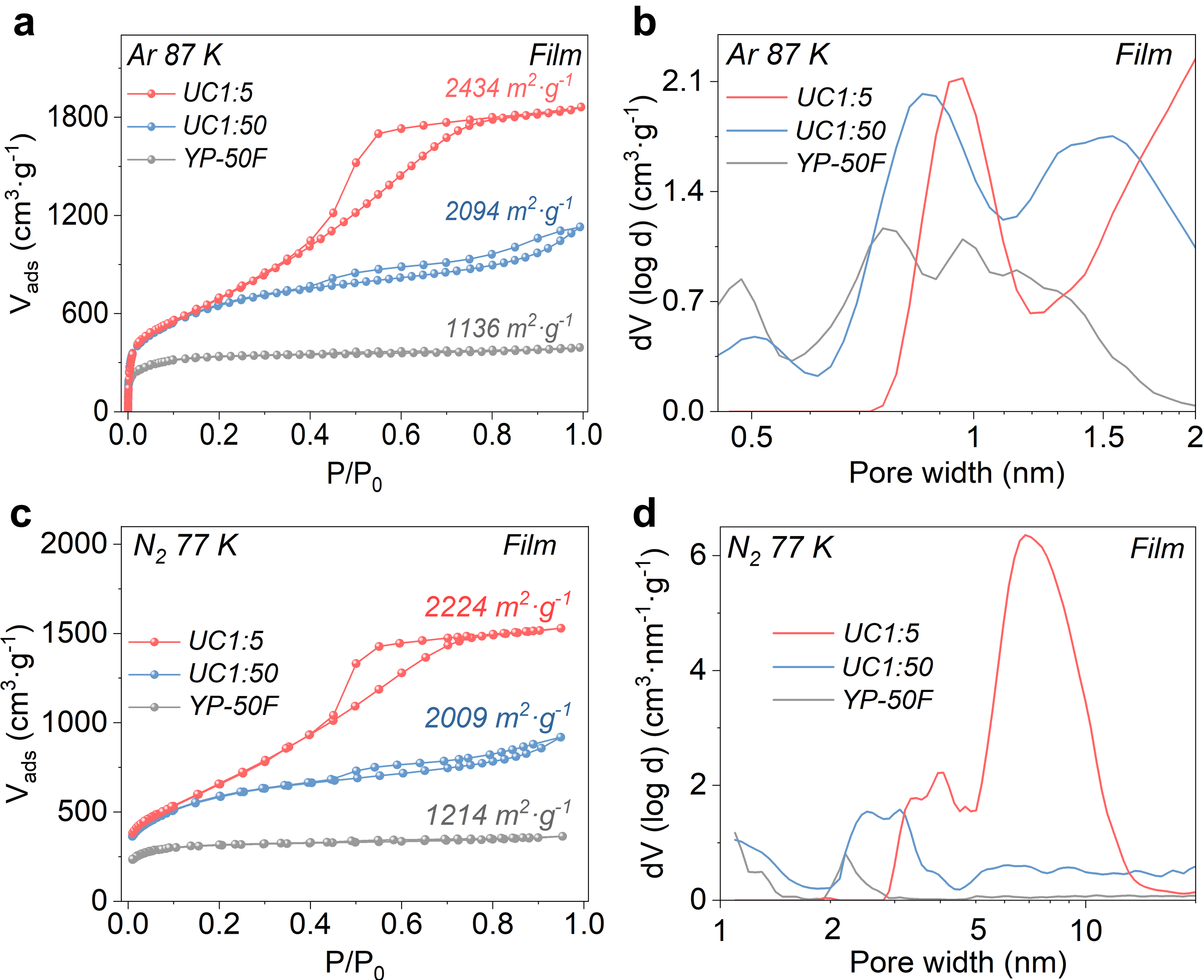


***Figure S9.*** Pore structure characterizations of UC1:5, UC1:50, and YP-50F electrode films, consisting of 80 wt% porous carbon, 10 wt% Super P carbon black, and 10 wt% PTFE binder. (a) Ar sorption isotherms at 87 K and (b) corresponding pore size distributions. (c) N_2_ sorption isotherms and (d) corresponding pore size distributions of electrode films.


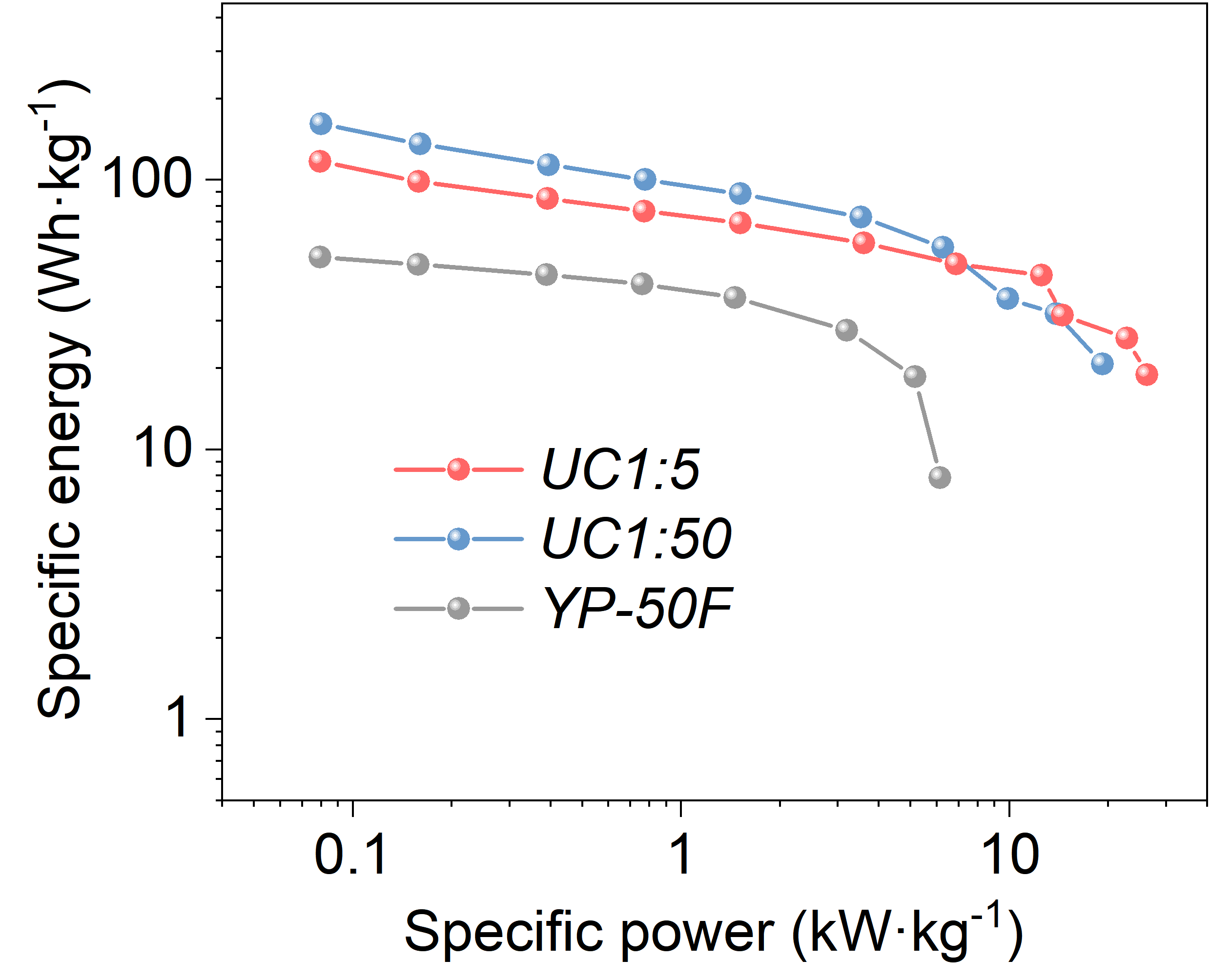


**Figure S10.** Ragone plots of UC1:5, UC1:50, and YP-50F in Zn-ion hybrid capacitors. The specific power and energy are calculated based on the mass of porous carbons in the electrodes.


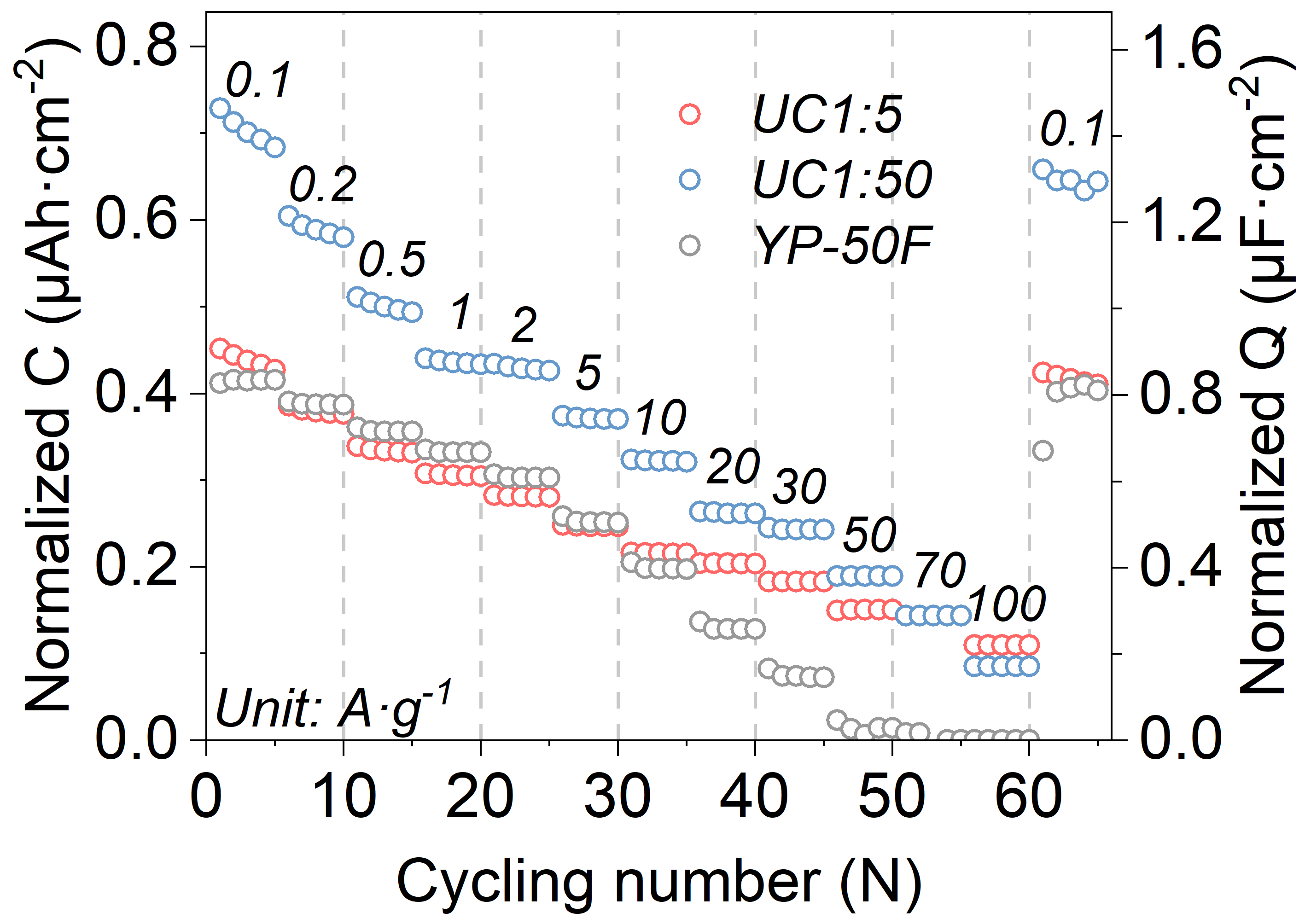


**Figure S11.** Normalized specific capacity (C, left axis) and capacitance (Q, right axis) by SSAs of UC1:5, UC1:50, and YP-50F in Zn-ion hybrid capacitors.

YP-50F exhibits normalized capacities/capacitances comparable to UC1:5 at current densities up to 10 A·g^−1^, but significantly drops at higher currents. This behavior is attributed to the presence of ultramicropores (< 0.86 nm) in YP-50F, which are too small to accommodate Zn(H₂O)₆²⁺ ions without the slow desolvation of their primary hydration shells, thereby limiting the accessible surface area. Nevertheless, Zn(H_2_O)_6_^2+^ can still enter larger micropores (0.86−2 nm), where the smaller ion-carbon distance partially compensates for the reduced surface area. As a result, YP-50F achieves capacitance comparable to UC1:5 at low current densities. This highlights the contribution of micropores to increase capacitance, and the role of mesopores to ensure rate capability.


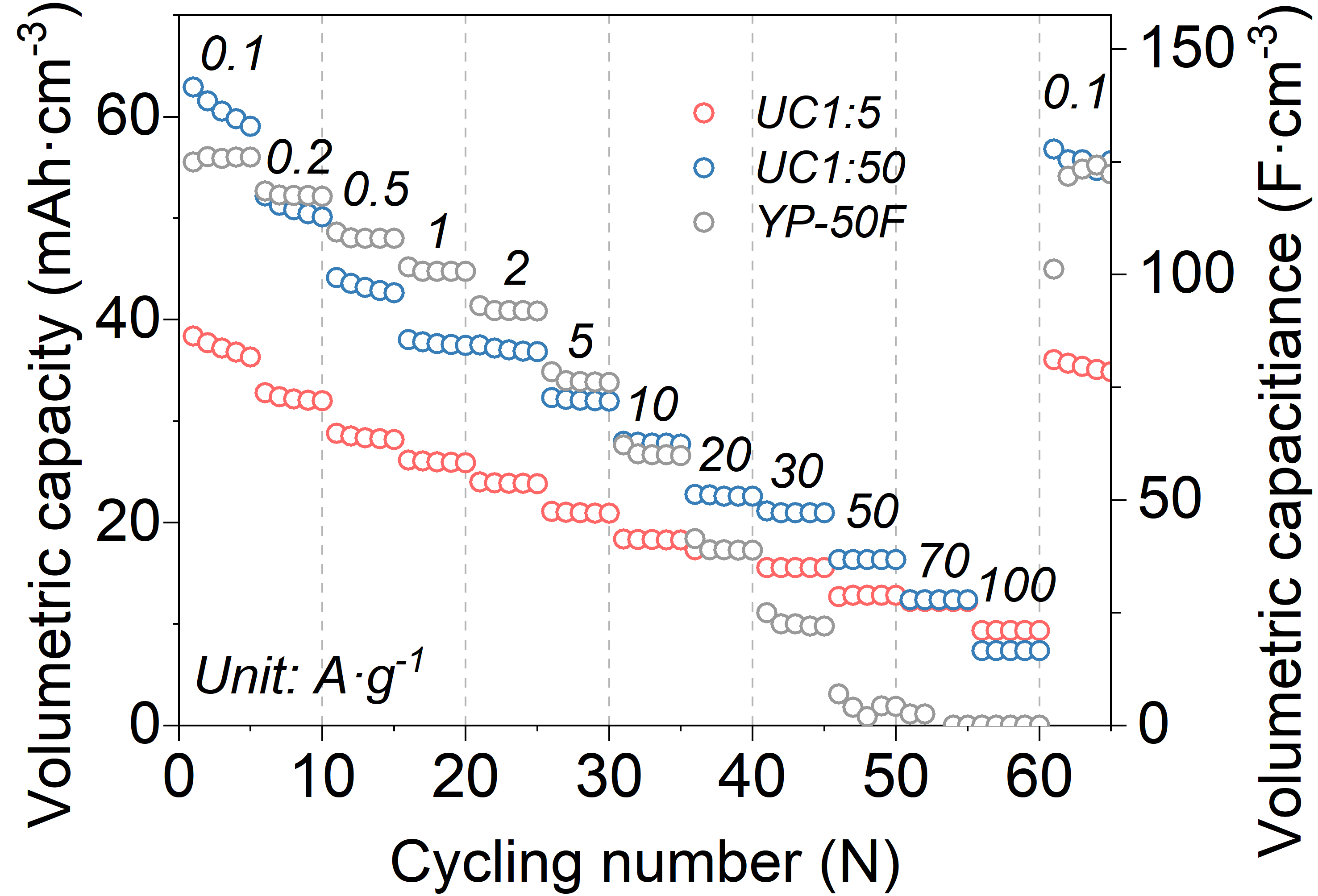


***Figure S12.*** Volumetric capacity (left axis) and capacitance (right axis) of UC1:5, UC1:50, and YP-50F cathodes in Zn-ion hybrid capacitors, measured over a current density of 0.1−100 A·g^−1^. The volumetric values are calculated based on the volumes of the total electrodes.


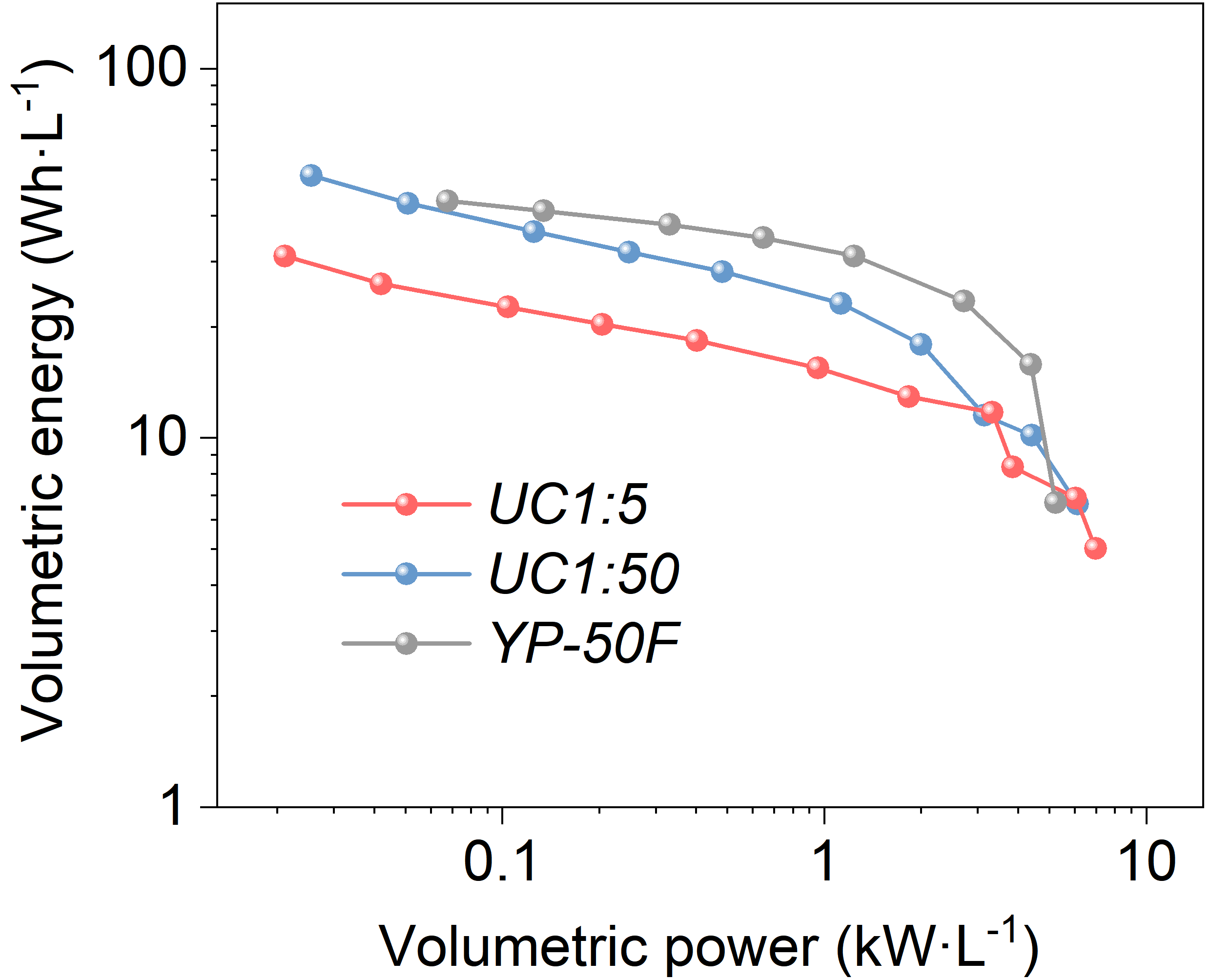


***Figure S13.*** Volumetric energy and power of Zn-ion hybrid capacitors based on UC1:5, UC1:50, and YP-50F cathodes. The volumetric values are calculated based on the volumes of the total electrodes.


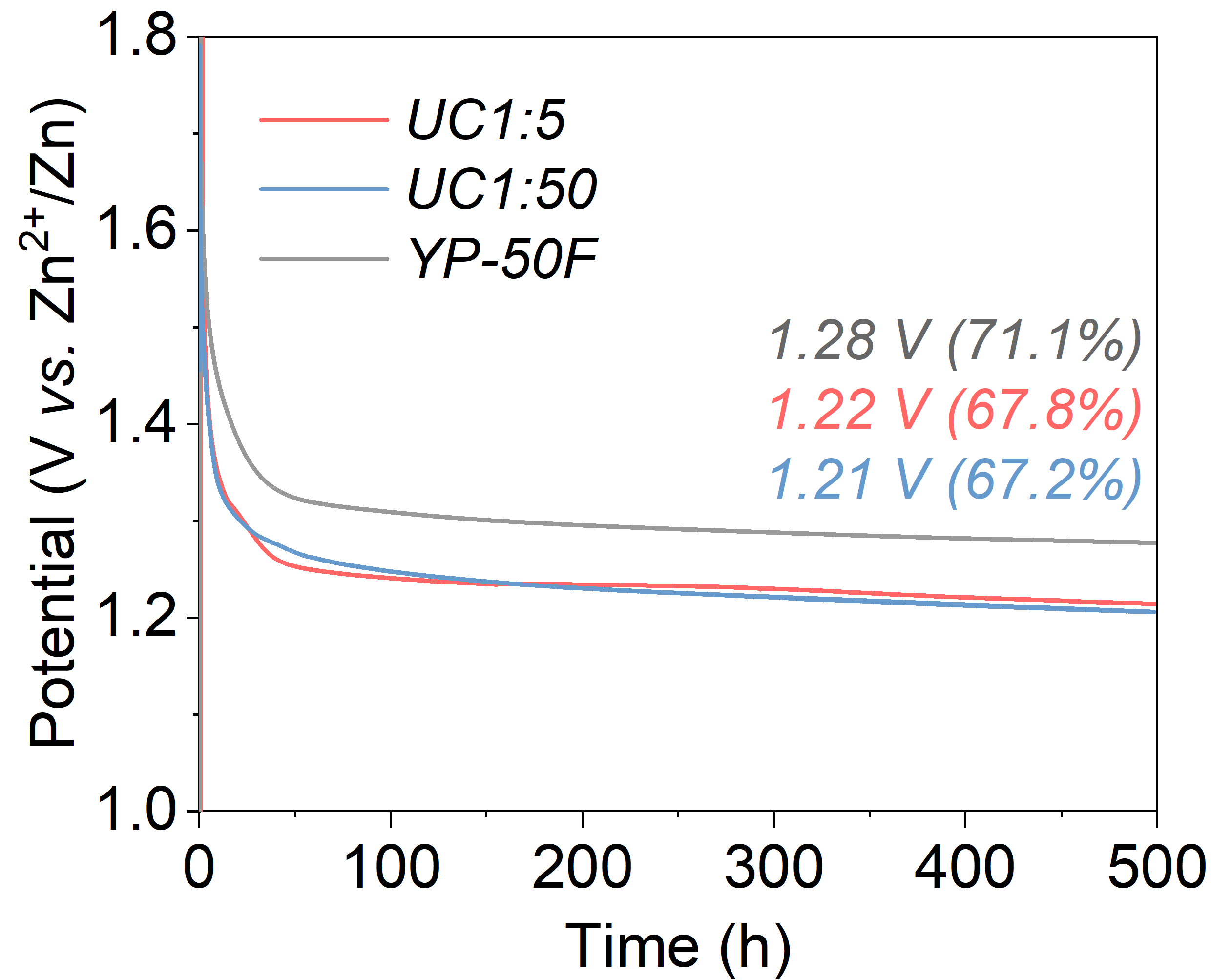


**Figure S14.** Self-discharge curves of UC1:5, UC1:50, and YP-50F as cathodes in Zn-ion hybrid capacitors after charging to 1.8 V vs. Zn^2+/^Zn over 500 hours.

**
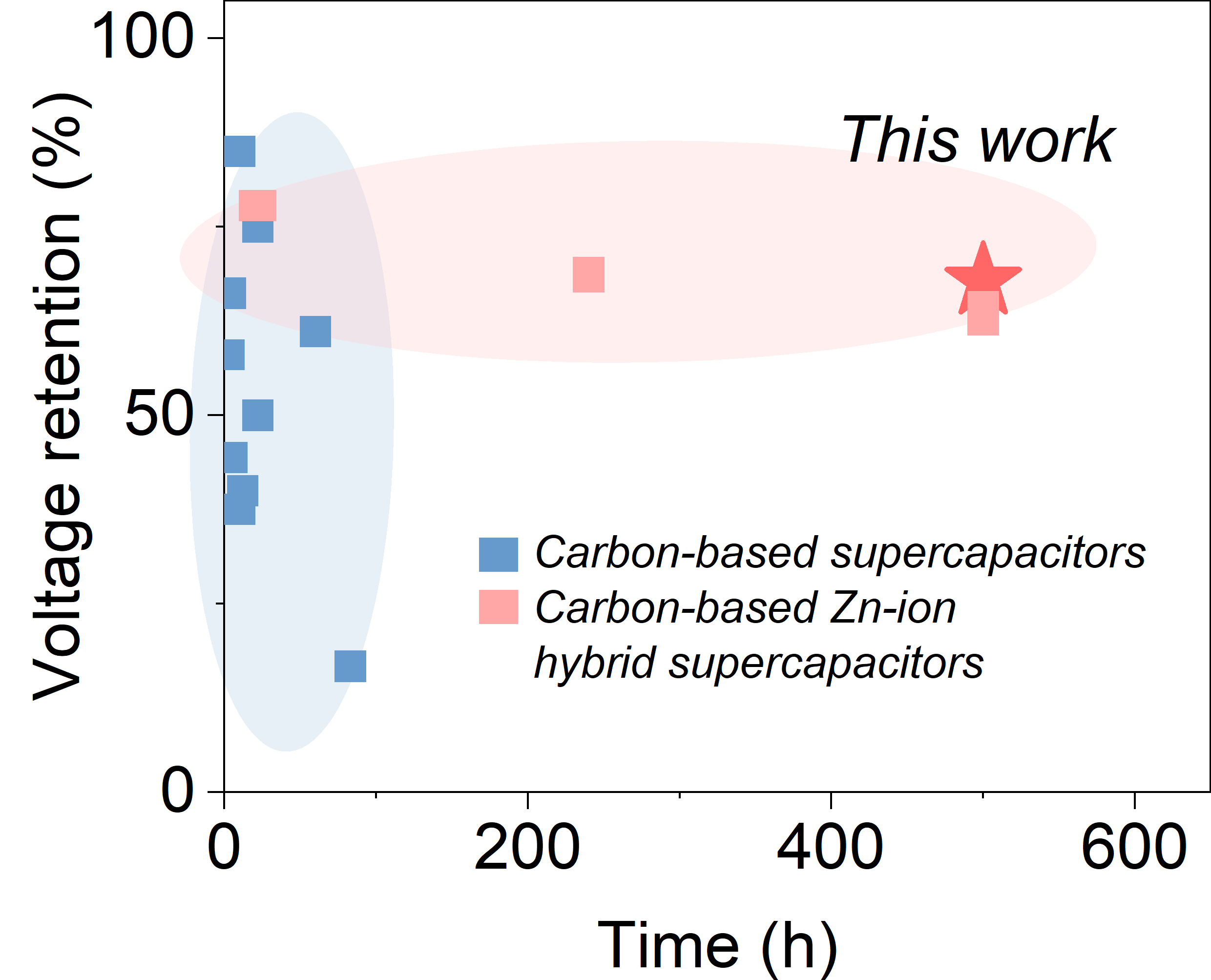
**

**Figure S15.** Voltage retention vs. time on self-discharge, comparing with Zn-ion hybrid capacitors and symmetric supercapacitors using carbon electrodes, with reference to **Tables S4** and **S5** for additional data.

The self-discharge is much more pronounced in symmetrical supercapacitors with carbon electrodes, which typically experience rapid voltage drops within just a few hours. In contrast, Zn-ion hybrid capacitors demonstrate enhanced resistance to self-discharge (up to 500 hours), likely due to the presence of Zn anode, which provides stable electrode potential to mitigating voltage decay. Additionally, surface functional groups on the carbon electrodes contribute to reducing charge leakage by binding ions more effectively.


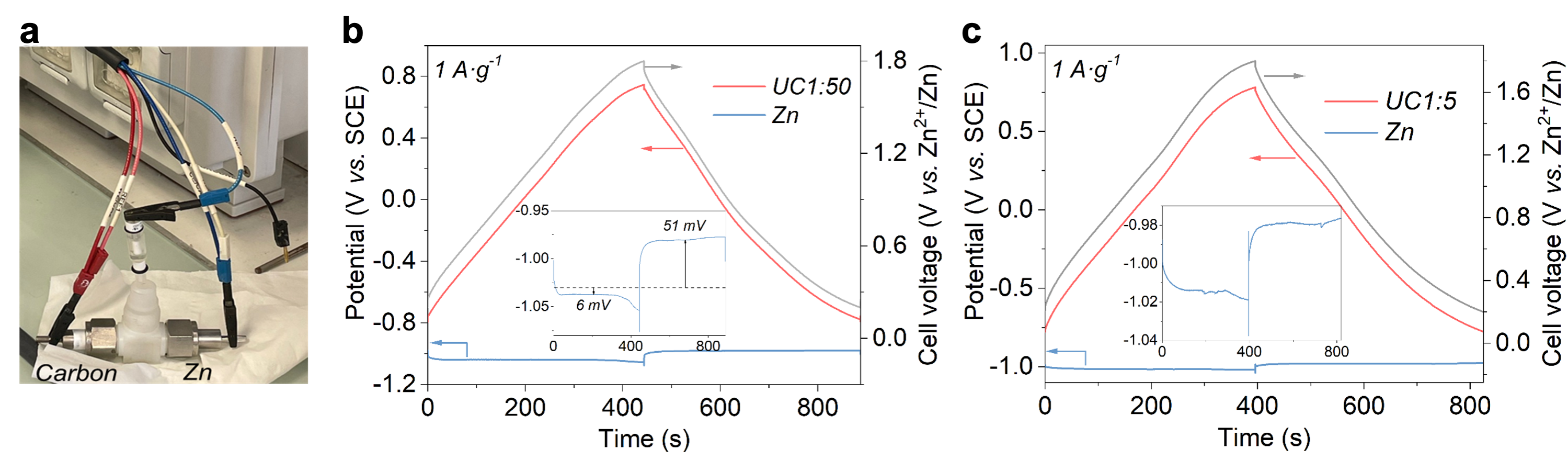


**Figure S16.** (a) Photo of a 3-electrode Swagelok cell set-up including a freestanding carbon film as working electrode, a Zn as counter electrode, and a saturated calomel electrode (SCE) serving as the reference electrode.

The set-up is configured to measure the potentials of Zn and carbon electrodes vs. SCE, and cell voltages between carbon (cathode) and Zn (anode). (b, c) Evolution of potentials of UC1:50/UC1:5 and Zn and cell voltages at a current density of 1 A·g^−1^_Carbon_.


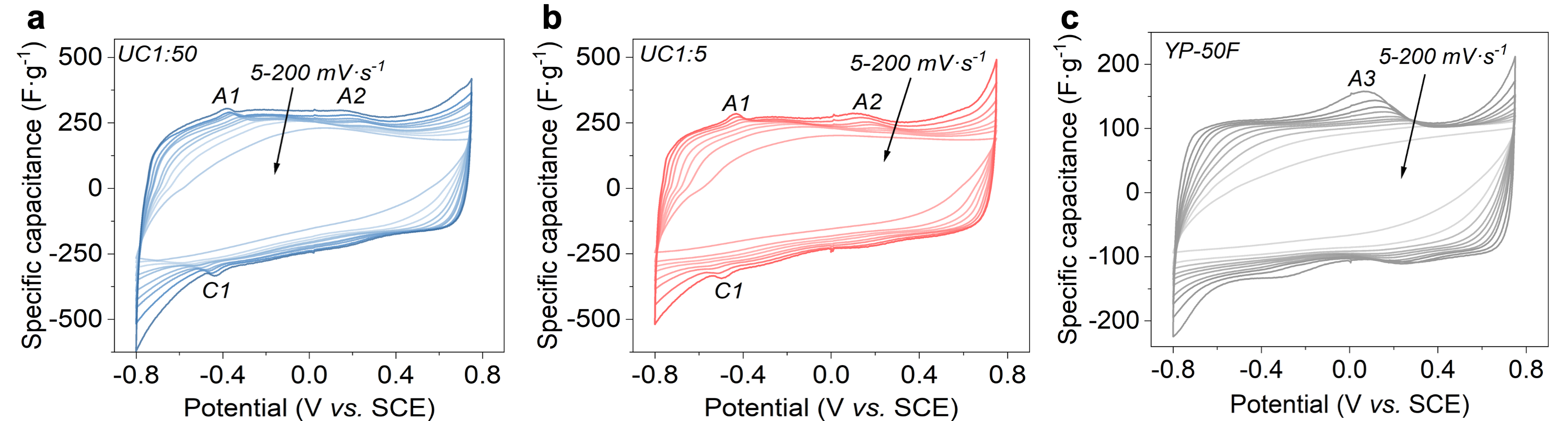


***Figure S17.*** CV analysis of (a) UC1:50, (b) UC1:5, and (c) YP-50F at 5−200 mV·s^−1^ in 3-electrode Swagelok cells.


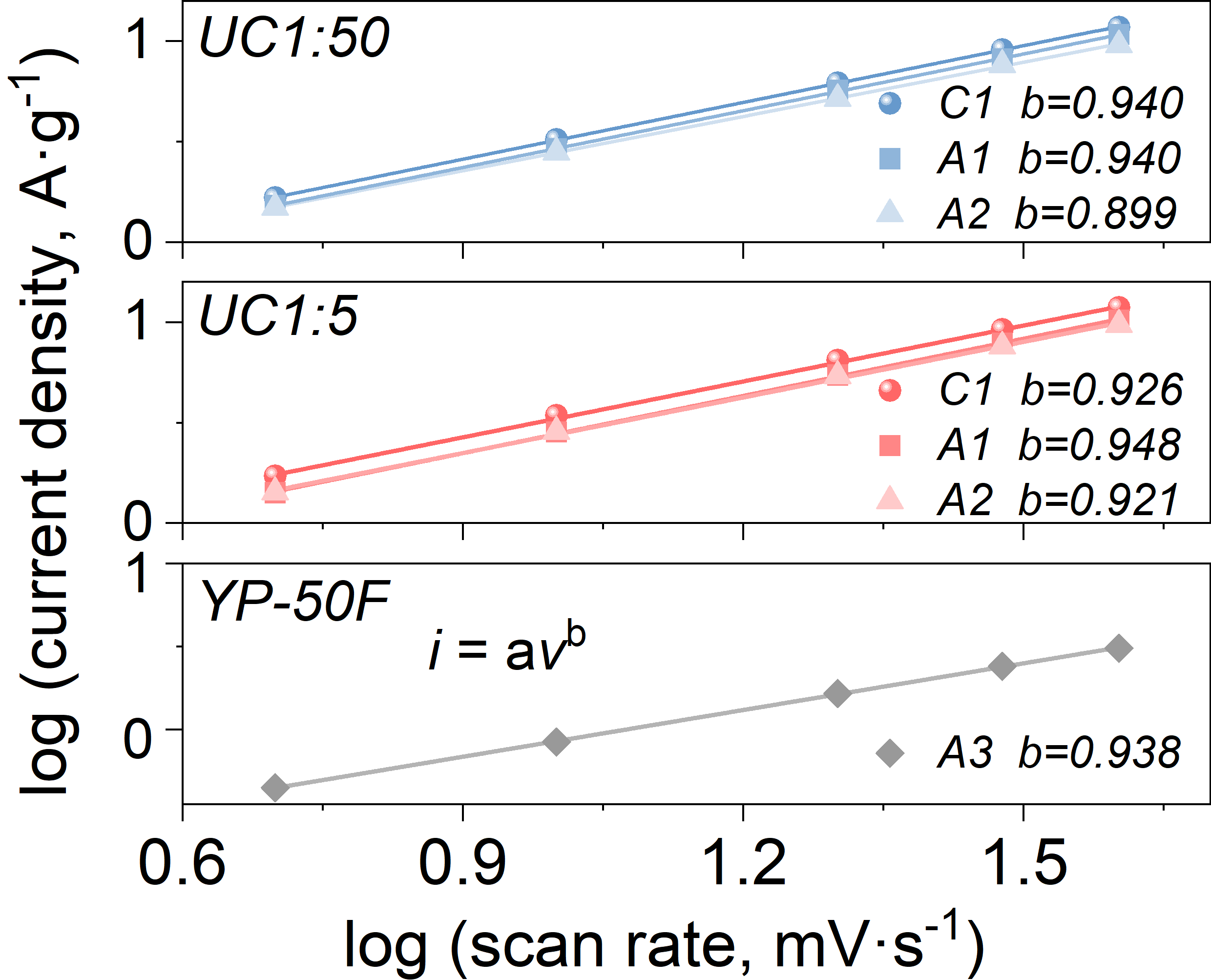


***Figure S18.*** Plots of log(current density) *vs.* log(scan rate) of current response at cathodic/anodic peaks and calculated b values based on CV analysis of UC1:50, UC1:5, and YP-50F at 5−40 mV·s^−1^.

The ion dynamics were further assessed utilizing the relationship between peak current (*i*) and scan rate (*v*) through the equation *i=av^b^*, where *a* and *b* are constants. A *b* value of 0.5 signifies a diffusion-controlled redox process, while *b* value of 1 denotes a fast surface-controlled capacitive process. The calculated *b*-values for cathodic/anodic peaks (5−40 mV·s^−1^) are close to 1, indicating a dominant capacitive process for all samples.


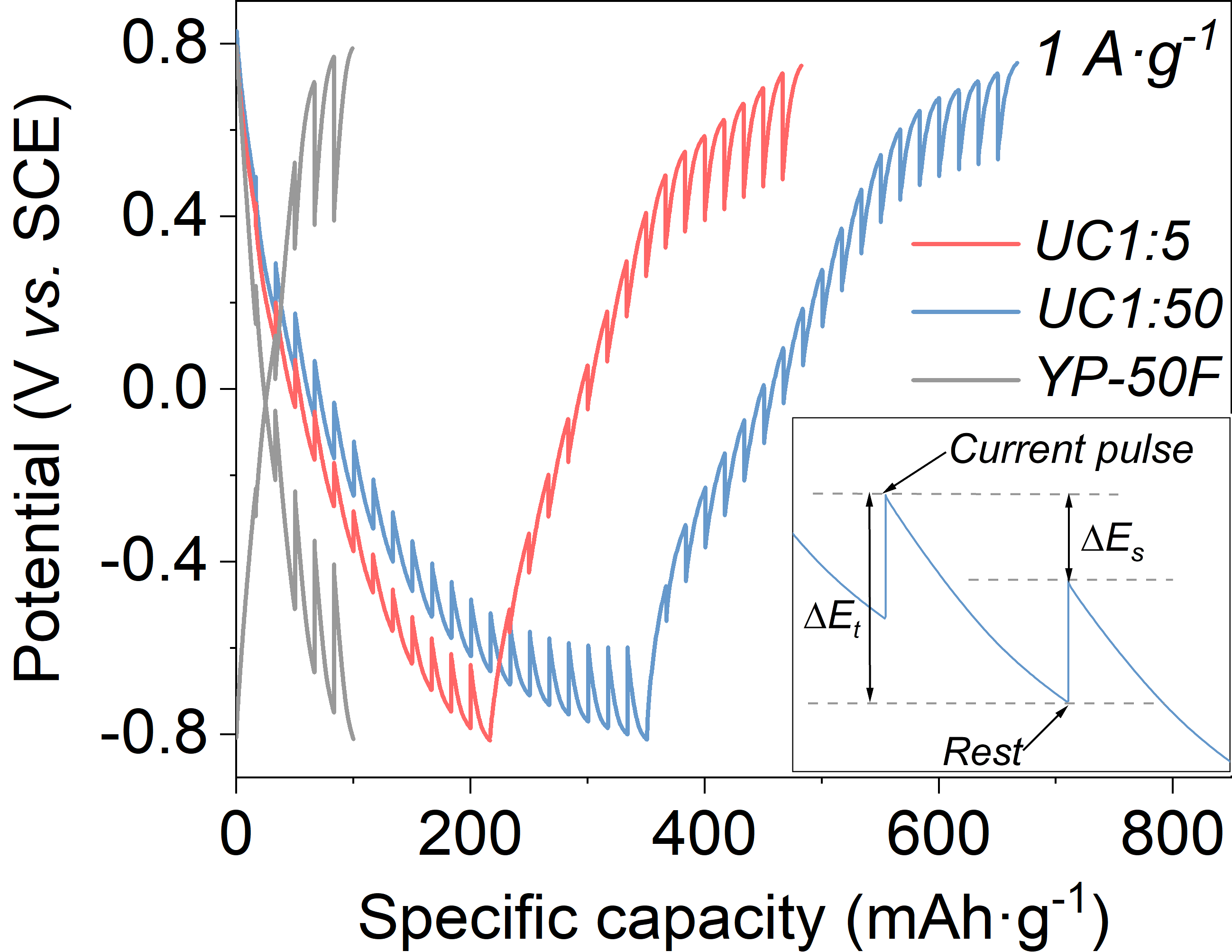


***Figure S19.*** Galvanostatic intermittent titration technique profiles of UC1:5, UC1:50, and YP-50F. Inset, the enlarged potential versus capacity profile of UC1:50.


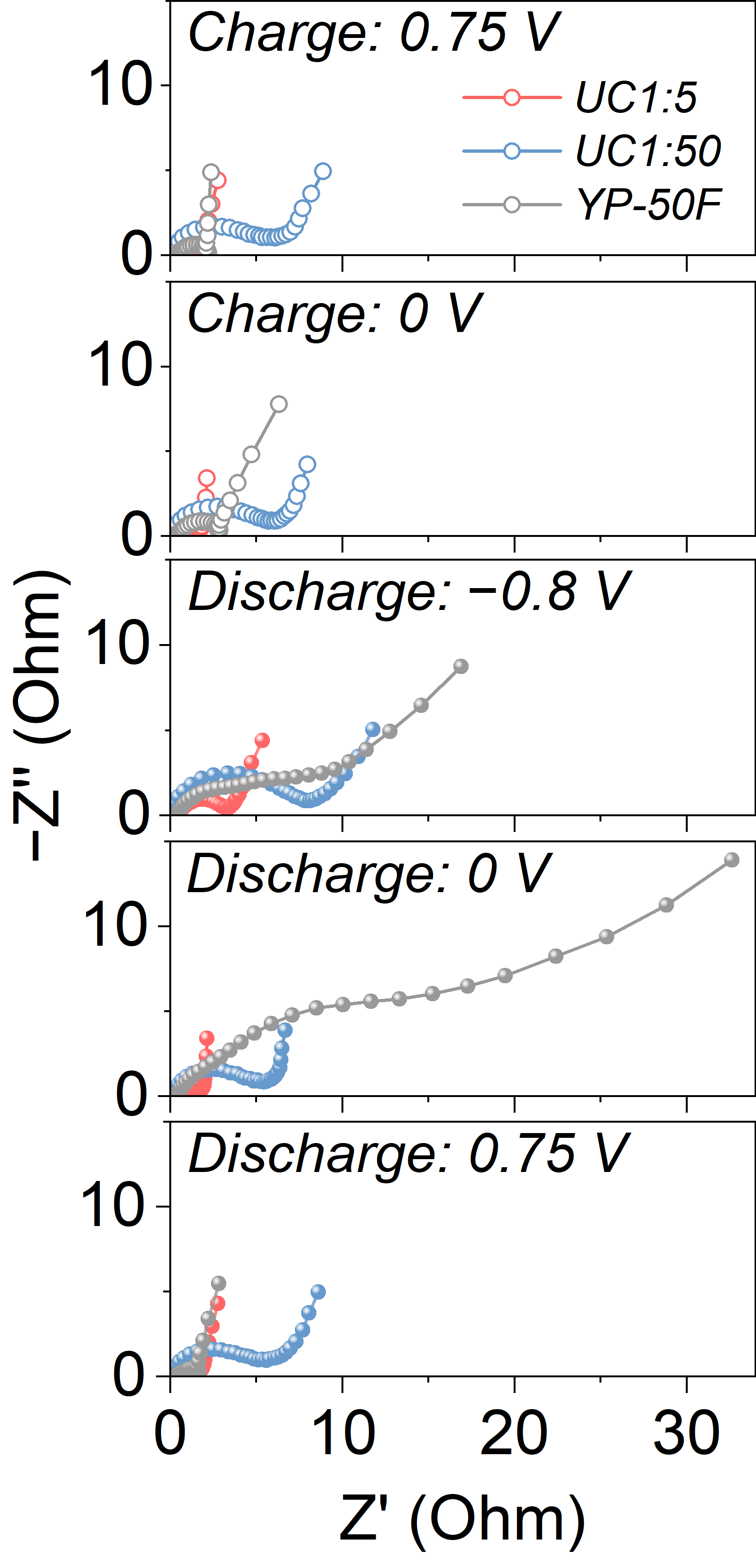


***Figure S20.*** Comparison of Nyquist plots of UC1:5, UC1:50, and YP-50F at different potentials *vs.* SCE*.*


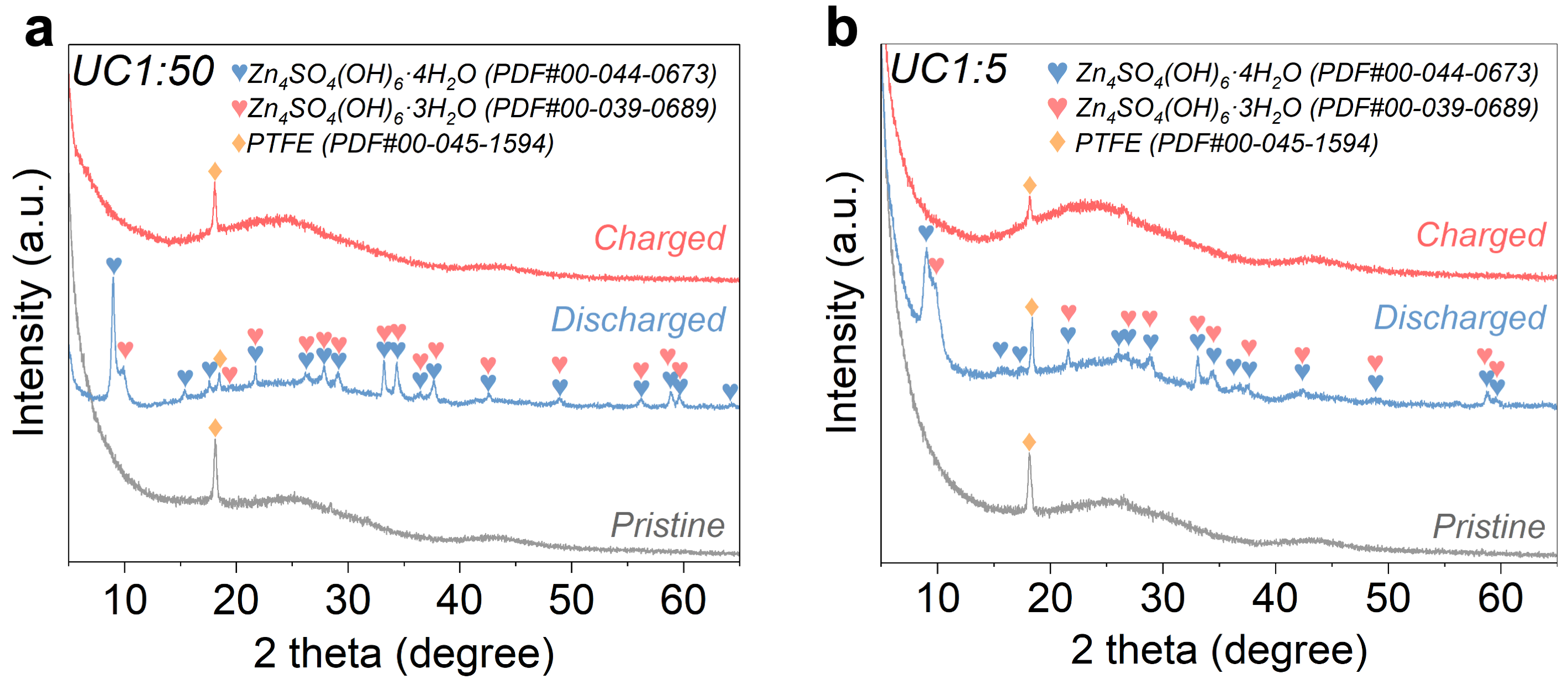


***Figure S21.*** X-ray diffraction patterns of UC1:50 and UC1:5 in the pristine state, after discharging to 0.2 V *vs.* Zn^2+^/Zn (~ −0.8 V *vs.* SCE), and recharging to 1.8 V *vs.* Zn^2+^/Zn (~0.75 V *vs.* SCE).

After discharge, both electrodes exhibit sharp diffraction peaks that match Zn₄SO₄(OH)₆·4H₂O and Zn₄SO₄(OH)₆·3H₂O, confirming the formation of crystalline zinc hydroxysulfate (ZHS). These diffraction peaks disappear after recharging, indicating reversible ZHS decomposition. These results support a reversible ZHS precipitation/dissolution mechanism during cycling.


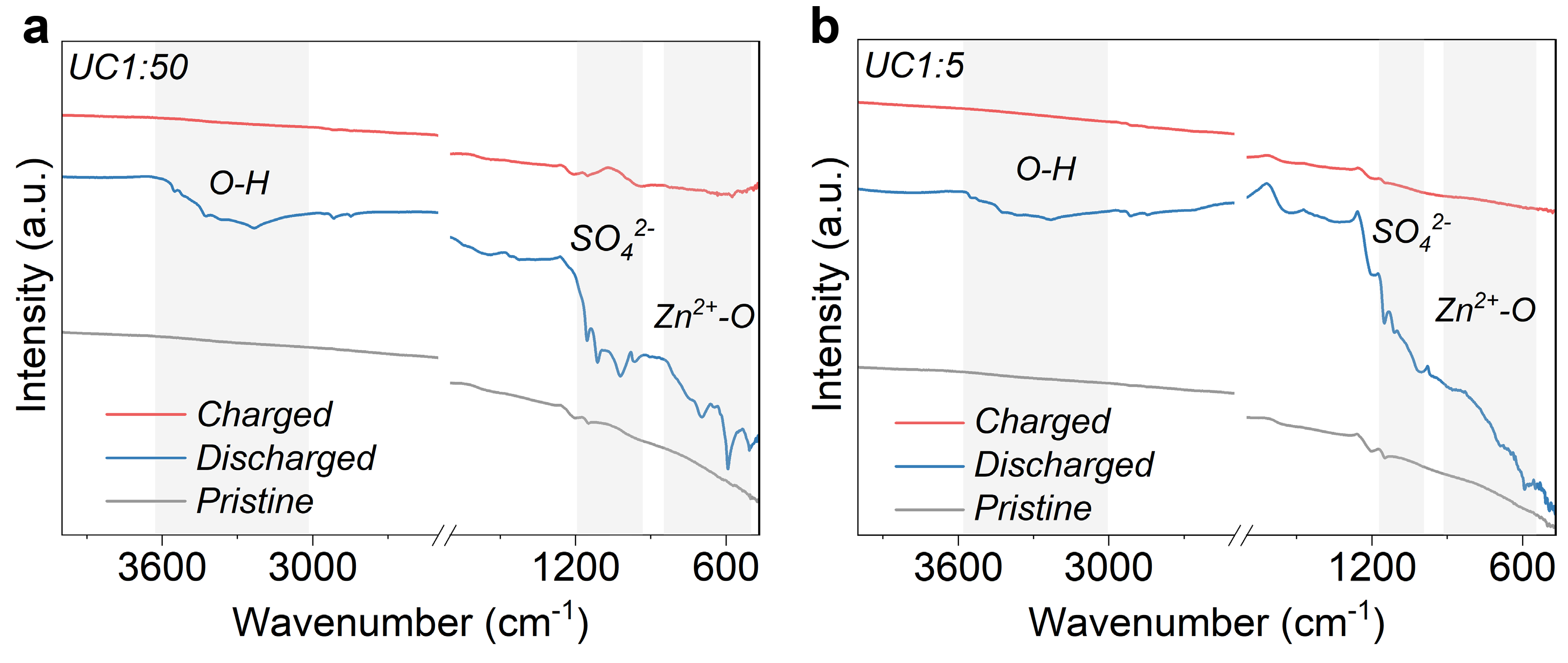


***Figure S22.*** Fourier transform-infrared spectra of UC1:50 and UC1:5 in the pristine form, after discharging to 0.2 V *vs.* Zn^2+^/Zn (~−0.8 V *vs.* SCE), and recharging to 1.8 V *vs.* Zn^2+^/Zn (~0.75 V *vs.* SCE).

Upon discharging, characteristic absorption bands emerge corresponding to O–H stretching (~3600–3200 cm⁻¹), SO₄²⁻ vibrations (~1200–1000 cm⁻¹), and Zn²⁺–O vibrations (~800–500 cm⁻¹),^[2]^ consistent with the formation of ZHS on the carbon surface. After recharging, these peaks disappear, indicating the dissolution of ZHS and recovery of the carbon surface. This reversible evolution of surface functional groups supports ZHS-based charge storage mechanism.


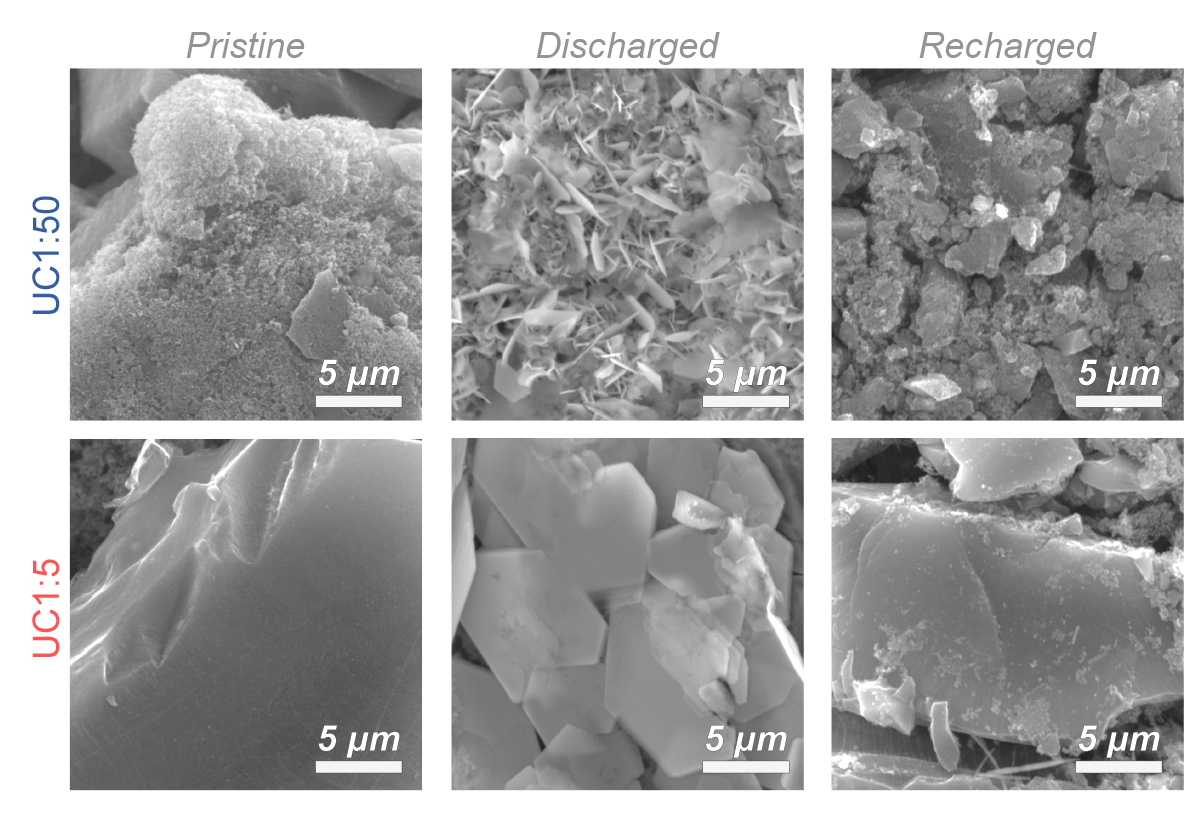


***Figure S23.*** Scanning microscopic images of UC1:50 and UC1:5-based cathodes in the pristine state, after discharging to 0.2 V *vs.* Zn^2+^/Zn (~ −0.8 V *vs.* SCE), and recharging to 1.8 V *vs.* Zn^2+^/Zn (~0.75 V *vs.* SCE).

Upon discharging, micrometer-sized hexagonal plate-like structures form on both cathodes, corresponding to zinc hydroxysulfate hydrates (ZHS). UC1:50 shows finer, more fragmented deposits, while UC1:5 exhibits larger platelets. This difference is likely attributed to the distinct surface morphologies of carbon materials. UC1:50, with a rougher surface, provides more nucleation sites for the growth of smaller ZHS crystallites. In contrast, the relatively smooth surface of UC1:5 favors the formation of larger ZHS platelets. Upon recharging, ZHS deposits dissolve, and the carbon surfaces are re-exposed.


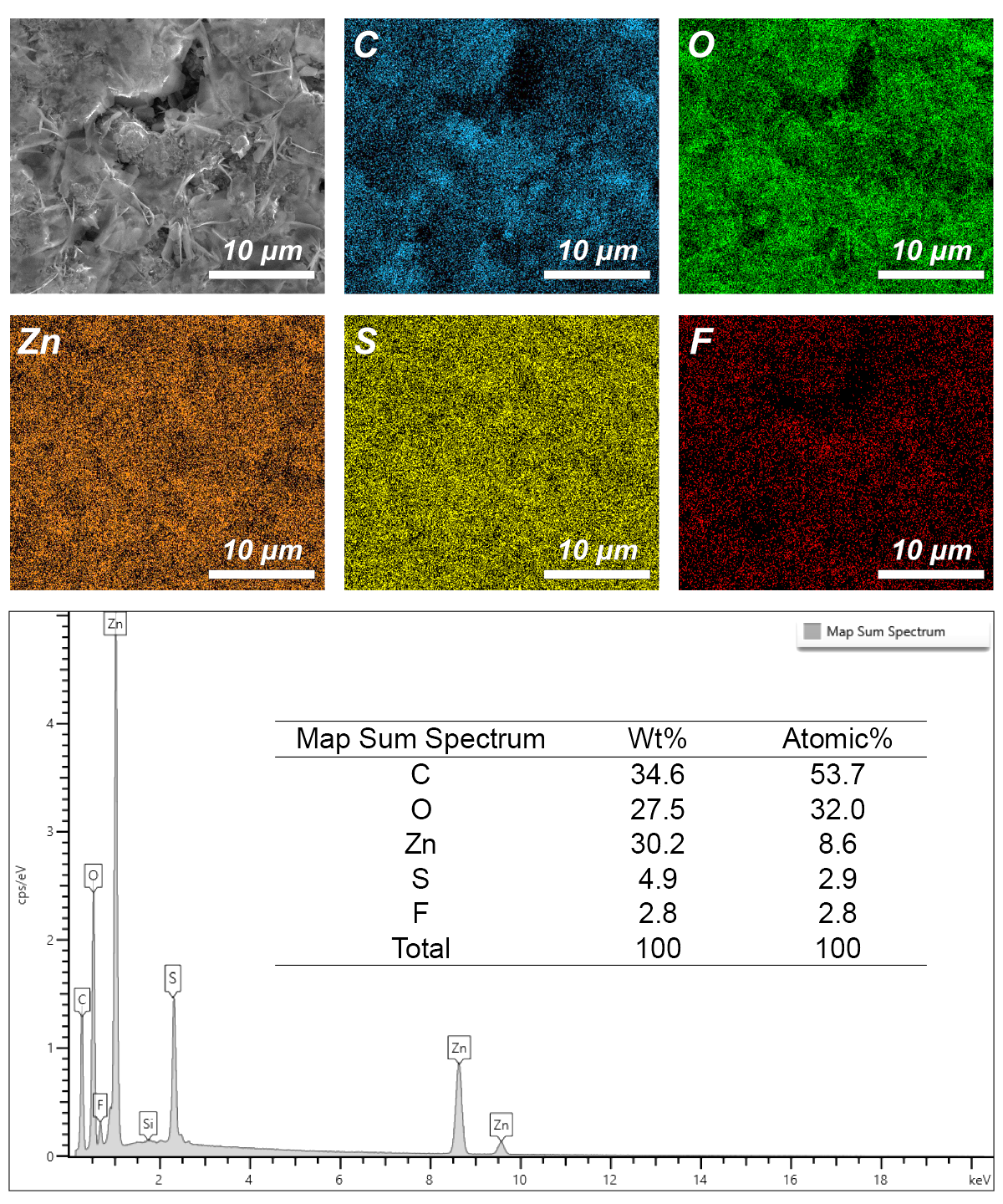


***Figure S24.*** Scanning microscopic images coupled with energy-dispersive X-ray spectroscopy of UC1:50 cathode after discharging to 0.2 V *vs.* Zn^2+^/Zn in a Zn-ion hybrid capacitor.

This EDX spectrum reveals evenly distributed signals of C, O, Zn, S, and F, indicating the formation of zinc sulfate species on the carbon surface. The presence of these elements is consistent with the precipitation of ZHS during discharge.


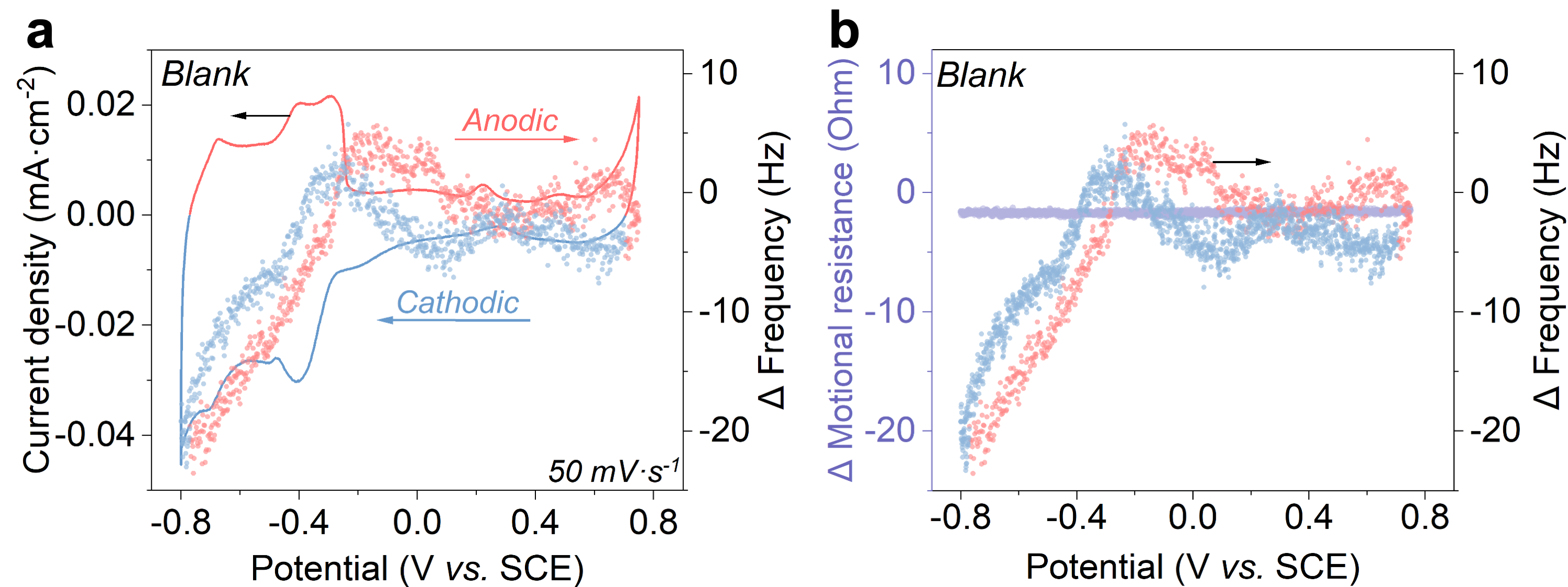


**Figure S25.** EQCM results of a blank Au-quartz electrode in 2 M ZnSO_4_ aqueous electrolyte. (a) CV at a scan rate of 50 mV⋅s^−1^ and accompanied EQCM frequency response; (b) Motional resistance and frequency response during CV scan.

The CV curves were subdivided into two sections based on electrode polarization: a cathodic region with a negative current response and an anodic region with a positive current response. In blank EQCM experiments, multiple redox peaks in the CV curve and notable frequency changes (∆f) between −0.2 and −0.8 V vs. SCE are observed, attributed to proton interactions with gold and the deposition/decomposition of ZHS on the gold electrode. Meantime, the motional resistance (∆R) remains largely unchanged, suggesting that the ZHS deposition has negligible effect on the viscoelastic properties of quartz crystal. This is likely because i) the rigid ZHS does not add additional resistance to the oscillating system, and ii) the formed ZHS has weak mechanical coupling to the crystal, exerting little effect on motional resistance.

**
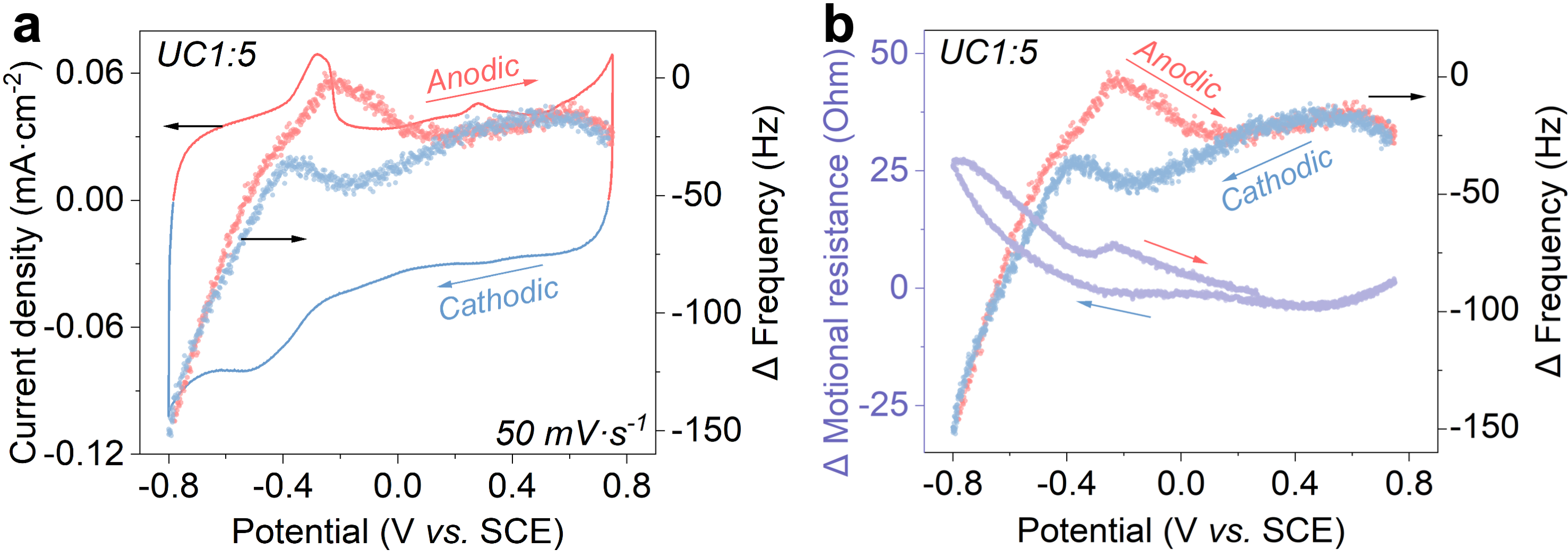
**

***Figure S26.*** EQCM results of a UC1:5 coated Au-quartz electrode in 2 M ZnSO_4_. (a) CV curve and EQCM frequency response; (b) Motional resistance and frequency change during polarization of UC1:5-coated Au-quartz at 50 mV⋅s^−1^.

In ***Figure S26a***, a significant increase in CV area indicates the contribution of porous carbon to charge storage compared to Au background. The frequency changes at low potentials are attributed to proton interactions with carbon and the deposition/decomposition of ZHS on the carbon. The starting and ending points of frequency curves overlap, indicating no irreversible mass change occurs after the CV scan.

In ***Figure S26b***, unlike the blank Au-quartz electrode, the motional resistance (*∆R*) changes at low potentials, suggesting that the formation of ZHS precipitates within carbon nanopores influence the viscoelastic properties of quartz crystal. This likely results from a strong mechanical coupling of carbon to the Au-quartz crystal through the binder, allowing the formed ZHS to add motional resistance to the oscillating system.^[3]^ Nevertheless, the validity of the gravimetric mode of EQCM measurements was confirmed by tracking *∆R* during the experiments. The ratio *∆R/∆f* was found below 15%, validating the gravimetric mode and justifying the use of Sauerbrey’s equation to calculate electrode mass change (*∆m*) from the frequency change (*∆f*) in this potential range.

*
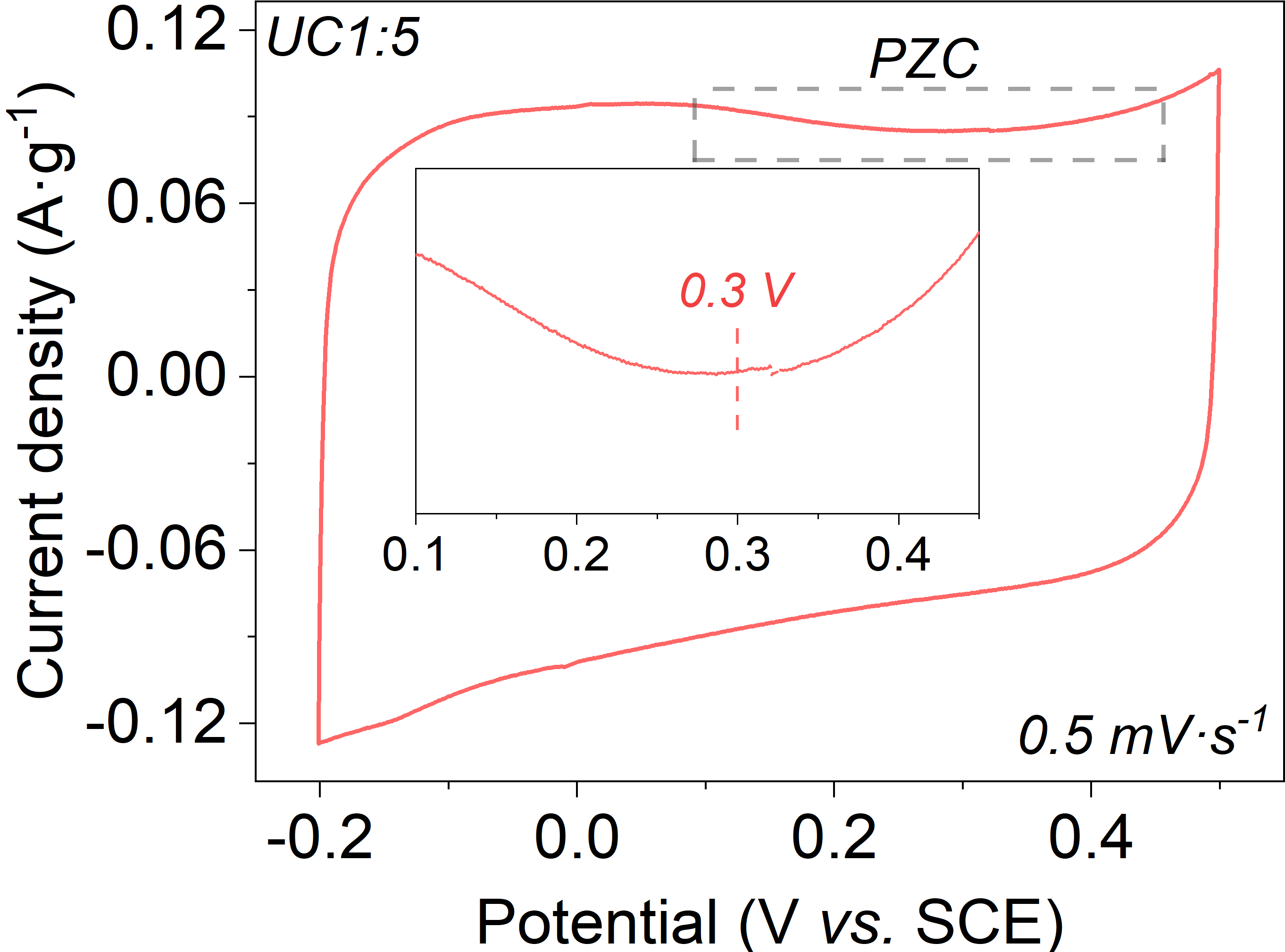
*

***Figure S27.*** CV curves of UC1:5 at a scan rate of 0.5 mV·s^−1^ with an inset highlighting enlarged profiles to reveal the potential of zero charge (PZC).

PZC is the potential where the overall charge on the carbon surface is neutral, including both electronic and ionic charges. It defines the border between regions where the surface electronic charge, negative or positive, is balanced with cations or anions at more cathodic or anodic potentials than PZC.^[4]^ PZC is measured at around 0.3 V *vs.* SCE, meaning a positive polarization (positive surface electronic charge) is required to balance the negative ionic charges on the carbon surface, achieving the electrode to zero charge. This suggests that SO_4_^2−^ ions are preferentially adsorbed on the carbon surface at open circuit potential (*ca.* 0.2 V *vs.* SCE).


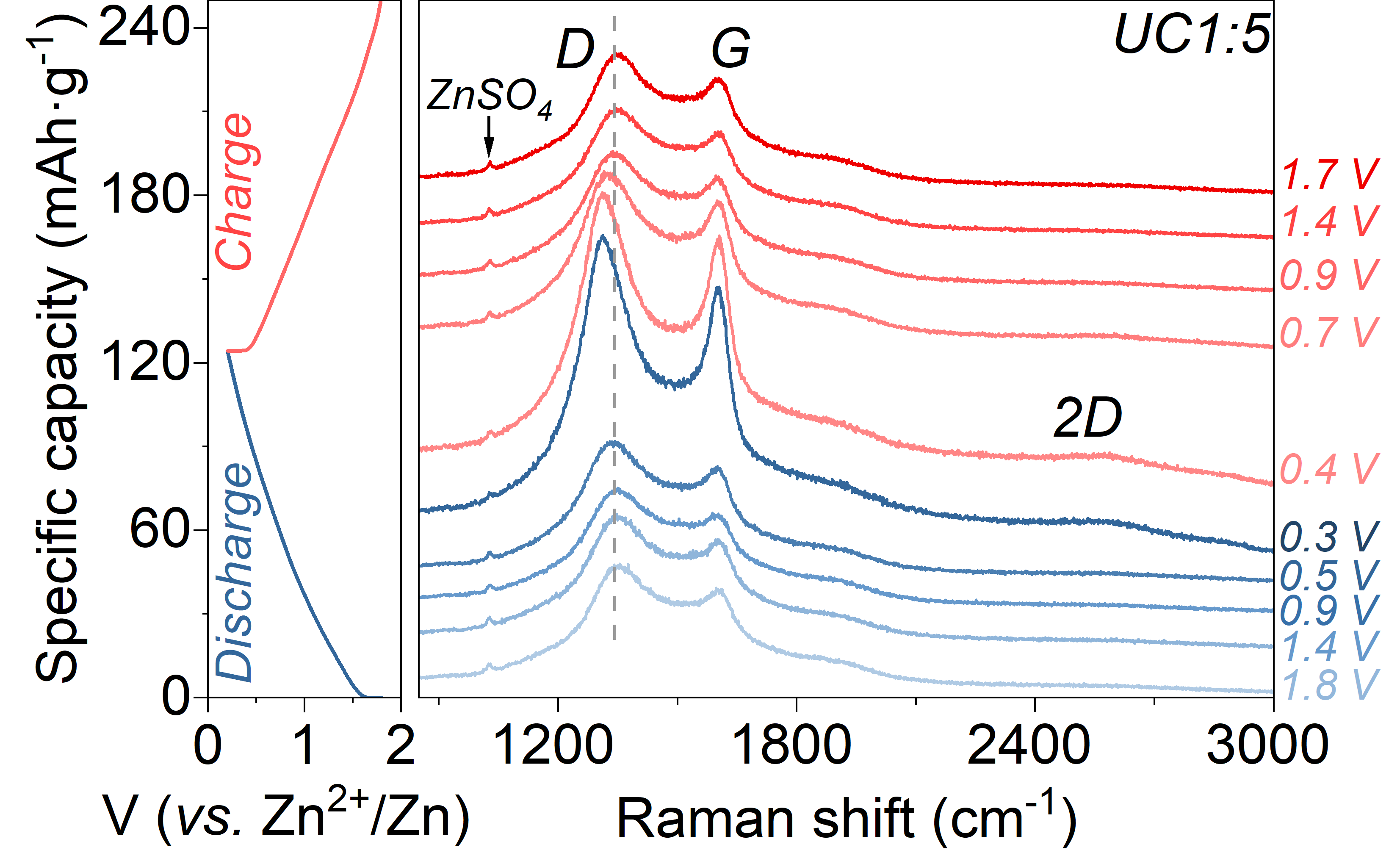


***Figure S28.*** *In situ* Raman spectroscopy of UC1:5 cathode in a Zn-ion hybrid capacitor during discharge/charge with a potential window of 0.2 to 1.8 V *vs.* Zn^2+^/Zn.

The D and G bands show reversible intensity changes with potential, indicating electrochemical negative doping of carbon material and its interaction with hydrated Zn²⁺ ions.^[5,6]^


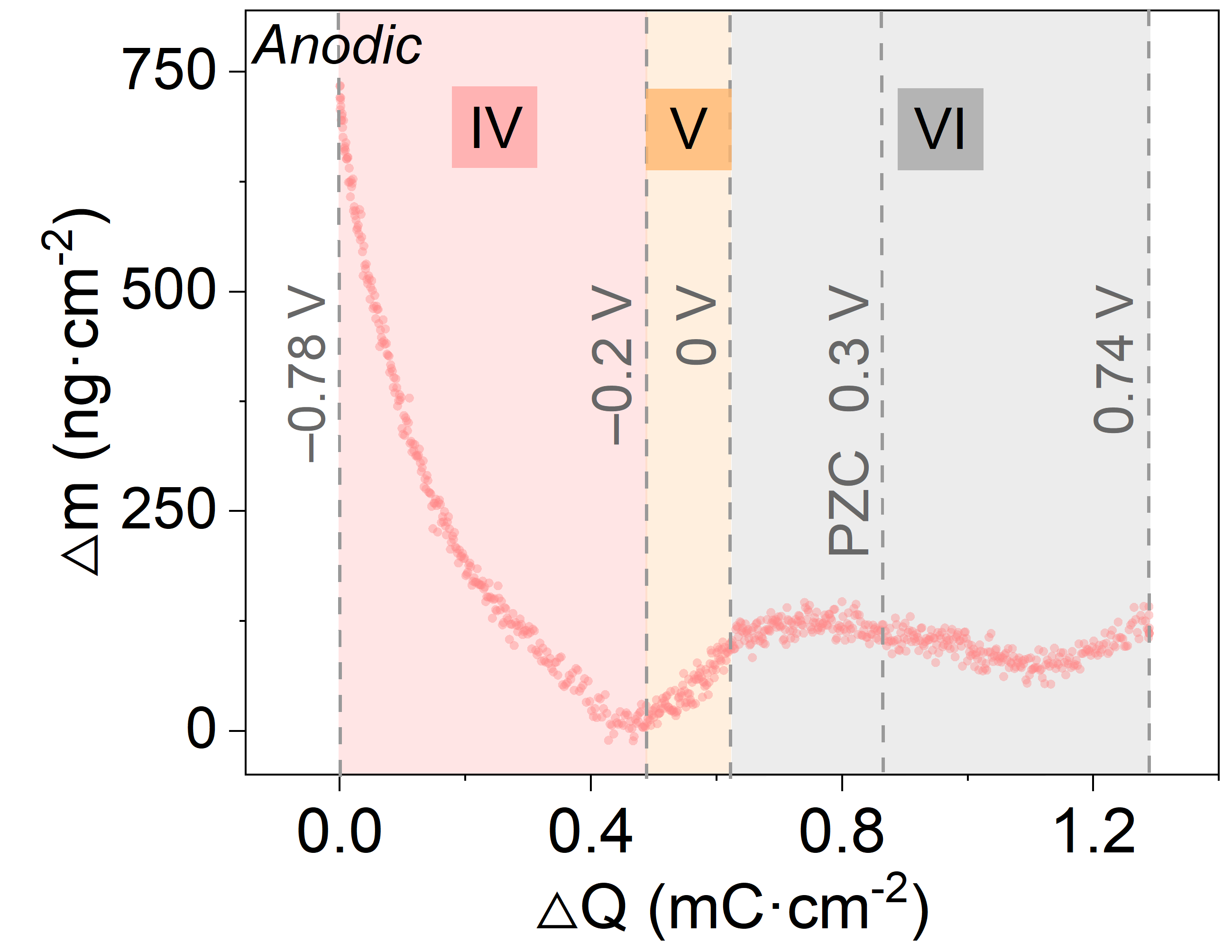


**Figure S29.** Electrode mass change (Δm) vs. accumulated positive charge change (ΔQ) during an anodic scan, with three distinct regions highlighted in different colors, measured in an EQCM set-up including UC1:5-coated gold-quartz electrode in 2 M ZnSO_4_ aqueous solution. Vertical grey dashed lines mark the cut-off potentials for each region.

In Region IV (−0.8 to −0.2 V vs. SCE), an oxidation peak is observed in the CV curve, accompanied by a frequency increase and decrease in motional resistance (**Figure S26a**), corresponding to proton evolution and ZHS dissolution. Further, the mass increase in Region V is attributed to the rehydration of Zn ions as the anodic charging. Between 0 and 0.74 V (Region VI), no noticeable mass change is observed, aligning with a combination process of solvated Zn ion desorption and SO_4_^2−^ adsorption.


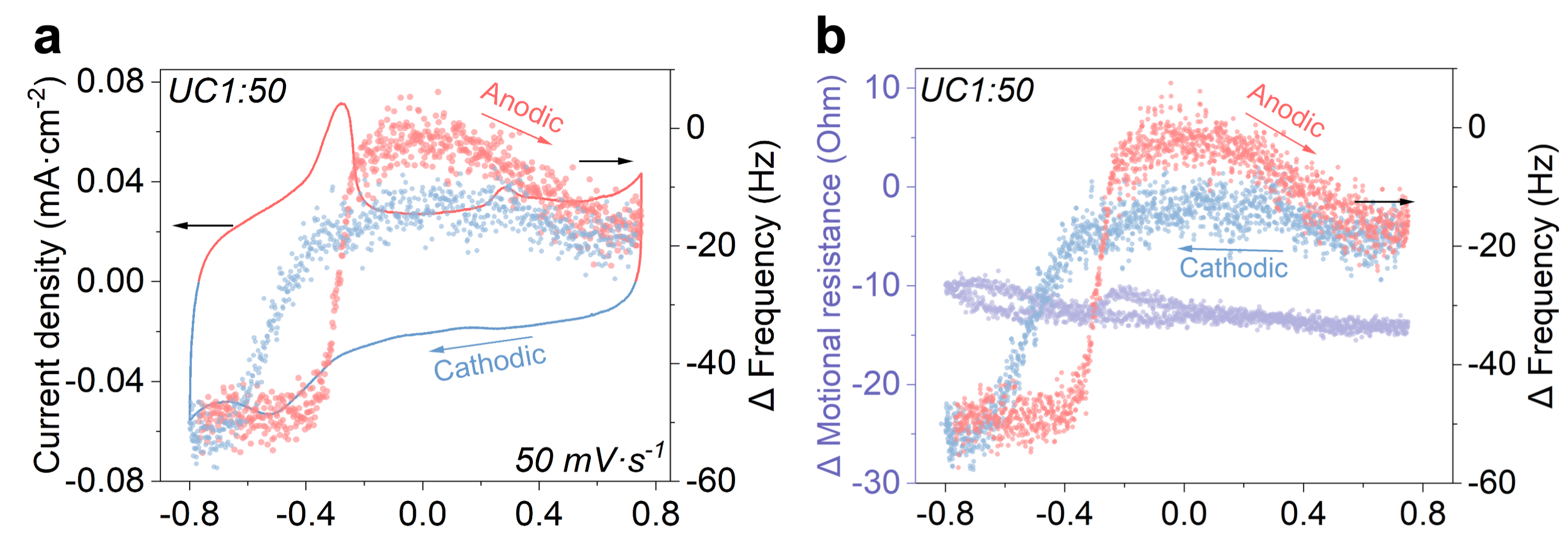


**Figure S30.** EQCM results of UC1:50 coated Au-quartz electrode in 2 M ZnSO_4_ aqueous solution. (a) CV curve at a scan rate of 50 mV⋅s^−1^ and EQCM frequency response; (b) Corresponding motional resistance changes and frequency change during CV scan.

For UC1:50, the CV and frequency response are generally similar to UC1:5, though some differences likely reflect pore size effects. In the cathodic scan of UC1:50, no noticeable frequency change (potentially linked to dehydration of hydrated Zn ion) is observed around 0 V vs. SCE. Firstly, this is likely because water molecules released during dehydration process remain trapped within the smaller pores of UC1:50 instead of diffusing out to the electrolyte reservoir, due to slower transfer. Secondly, this is likely because only Zn ions with a lower degree of hydration can enter the smaller pores of UC1:50 during this measurement, resulting in less dehydration and water expulsion compared with UC1:5.

During the anodic process, the anodic peaks associated with the electrochemical oxidation of H_ad_ appear at similar potentials (−0.3 V vs. SCE) for both UC1:5 and UC1:50. However, ZHS dissolution (indicated by an increase in Δf) occurs later at higher potentials for UC1:50 (−0.4 V vs. SCE) than UC1:5 (−0.8 V vs. SCE). This suggests that more H_ad_ needs to be oxidized to generate sufficient H^+^ in the pores of UC1:50 to lower the pH and initiate ZHS dissolution. Additionally, more energy is needed to drive species like Zn^2+^ and SO_4_^2−^ away from the smaller nanopores to the electrolyte reservoir.

***Table S1.*** Comparison of structure and CO_2_ uptake with some reported materials.

| Type | Materials | S_BET_  (m^2^·g^−1^) | V_Micro_  (cm^3^·g^−1^) | CO_2_ uptake at  273 K, 100 kPa  (mmol·g^−1^) | *Ref.* |
| --- | --- | --- | --- | --- | --- |
| Porous carbons | UC1:50 | 3245 | 1.26 | 8.3 | This work  ^[7]^ |
|  | CsAc600 | 1983 | 0.64 | 8.7 |  |
|  | cG@600SZ | 852 | / | 2.5 | ^[8]^ |
|  | PDVA-C-800 | 874 | 0.4 | 4.47 | ^[9]^ |
|  | SNMC-2-600 | 1884 | 0.78 | 7.38 | ^[10]^ |
|  | CS-Py-K100 | 1051 | 0.91 | 7.35 | ^[11]^ |
|  | FC4 | 941 | 0.93 | 4.05 | ^[12]^ |
|  | CPC2 | 2278 | 1.00 | 6.1 | ^[13]^ |
| Metal-organic frameworks | JLU-Liu46 | 1787 | 0.87 | 8.5 | ^[14]^ |
|  | [Sm (HL) (DMA)_2_]·DMA·2H_2_O | / | / | 3.8 | ^[15]^ |
|  | MOF-74(Ni) | 1080 | 0.43 | 8.29 | ^[16]^ |
|  | ZU-66 | 177 | 0.40 | 5.8 | ^[17]^ |
| Zeolites | Li-LSX | 660 | 0.32 | 4.31 | ^[18]^ |
| Porous organic polymers | TMCOP | 1104 | / | 3.12 | ^[19]^ |
|  | CTPP | 779 | 0.77 | 3.48 | ^[20]^ |
|  | PAF-26-COOMg | 572 | 0.28 | 2.8 | ^[21]^ |

***Table S2.*** Comparison of structure and water uptake with reported materials.

| Type | Materials | S_BET_  (m^2^·g^−1^) | V_Micro_  (cm^3^·g^−1^) | V_Meso_  (cm^3^·g^−1^) | V_Total_  (cm^3^·g^−1^) | Max. H_2_O uptake (g·g^−1^) | *Ref.* |
| --- | --- | --- | --- | --- | --- | --- | --- |
| Porous carbons | UC1:5 | 3200 | 1.26 | 2.90 | 3.16 | 2.2 | This work  ^[22]^ |
|  | UA-800-SZ | 2590 | 0.38 | 1.75 | 2.13 | 1.38 |  |
|  | C-HAT-CN-700 | 785 | 0.3 | / | / | 0.23 | ^[23]^ |
|  | RUF-p900-CO3 | 1075 | 0.510 | / | / | 0.50 | ^[24]^ |
|  | UM350 | 1420 | 0.516 | 0.516 | 1.32 | 0.64 | ^[25]^ |
|  | PMC550 | 2240 | 0.788 | 0.821 | 1.609 | 0.85 |  |
|  | CMK-8 | 1373 | / | / |  | 0.89 | ^[26]^ |
| Metal-organic frameworks | Cr-soc-MOF-1 | 4549 | / | / | / | 2.0 | ^[27]^ |
|  | MIL-101Cr | 2059 | / | / | / | 1.0 | ^[28]^ |
|  | UIO-66 | 1105 | / | / | 0.55 | 0.44 | ^[29]^ |
|  | MIL-160 | 1070 | 0.398 | / | / | ~0.36 | ^[30]^ |
|  | Co_2_Cl_2_(BTDD) | 1912 | / | / | / | 0.97 | ^[31]^ |
| Covalent triazine frameworks | bipy-CTF500 | 1548 | 0.64 | 0.71 | 1.35 | 0.44 | ^[32]^ |
| Covalent organic frameworks | TpPa-1 | 984 | / | / | / | 0.44 | ^[33]^ |
|  | COF-432 | 895 | / | / | 0.43 | 0.3 | ^[34]^ |

***Table S3.*** Pore structure analysis of porous carbons in powder and film

| Samples | | V_0−0.7 nm_^[d]^  (m^3^·g^−1^) | V_0.7−2 nm_^[d]^  (m^3^·g^−1^) | V_2−50 nm_^[e]^  (cm^3^·g^−1^) | | V_0−50 nm_^[f]^  (cm^3^·g^−1^) | | SSA_Ar_^[g]^  (m^2^·g^−1^) | | Retention ratio of SSA_Ar_^[h]^ | | Retention ratio of V_0.7−2 nm_ ^[h]^ | | Retention ratio of  V_2−50 nm_^[h]^ |
| --- | --- | --- | --- | --- | --- | --- | --- | --- | --- | --- | --- | --- | --- | --- |
| Powder^[a]^ | UC1:5 | 0 | 0.68 | 2.90 | 3.58 | | 3200 | | / | | / | | / | |
|  | UC1:50 | 0.09 | 1.17 | 1.11 | 2.37 | | 3245 | | / | | / | | / | |
|  | YP-50F | 0.14 | 0.52 | 0.17 | 0.93 | | 1783 | | / | | / | | / | |
| Film^[b]^ | UC1:5 | 0 | 0.54 | 2.12 | 2.66 | | 2434 | | / | | / | | / | |
|  | UC1:50 | 0.08 | 0.71 | 0.86 | 1.65 | | 2094 | | / | | / | | / | |
|  | YP-50F | 0.11 | 0.33 | 0.12 | 0.56 | | 1136 | | / | | / | | / | |
| Normalized porous carbon in film^[c]^ | UC1:5 | 0 | 0.68 | 2.65 | 3.33 | | 3042 | | 95% | | 100% | | 91% | |
|  | UC1:50 | 0.10 | 0.89 | 1.08 | 2.06 | | 2618 | | 81% | | 76% | | 92% | |
|  | YP-50F | 0.14 | 0.41 | 0.15 | 0.70 | | 1420 | | 80% | | 79% | | 88% | |

[a] Porous carbon powders were obtained through the direct thermal treatment of uric acid and CsAc, followed by washing and drying. [b] Film refers to a freestanding electrode film composed of 80 wt% carbon powder, 10 wt% Super P, and 10 wt% PTFE binder. Both powders and films were dried at 150 ^o^C for 20 hours before Ar and N_2_ sorption analyses. [c] Since Super P and PTFE are non-porous, and only porous carbons contribute to the sorption data, pore volumes and SSAs of films were normalized by the 80 wt% porous carbon content to assess pore blockage. [d] Micropore volumes determined from Ar sorption at 87 K using the QSDFT for Ar adsorbed on carbon with slit pores. [e] Pore size contributions and the related pore volume cuts were calculated from N_2_ sorption at 77 K using QSDFT for N_2_ adsorbed on carbon with slit/cylindrical/sphere pore shape. [f] Total pore volume was calculated as the sum of these individual pore volumes. [g] SSAs were evaluated at *P/P_0_* (0.05−0.30) of Ar sorption isotherms at 87 K or N_2_ sorption isotherms at 77 K based on the Brunauer-Emmett-Teller (BET) equation. [h] The retention ratio (%) represents the fraction of surface area or pore volume preserved in the film relative to the corresponding porous carbon powder. These values were calculated by normalizing the SSA or pore volume of porous carbon in the film (after correcting for 80 wt% carbon content) to the values measured for the powder form.

**Table S4.** Performance comparison of Zn-ion hybrid capacitors based on various cathode materials.

| Cathodes | | Active materials | Current collectors | Mass loading of active material  (mg·cm^−2^) | SSA  (m^2^·g^−1^) | Electrolyte | Voltage  window  (V) | Specific capacitance (F·g^−1^)  /Current  (A·g^−1^) | Specific capacity (mAh·g^−1^)  /Current (A·g^−1^) | Specific energy  (Wh·kg^−1^) | Specific power  (kW·kg^−1^) | Capacity retention/  Cycling number/  Current (A·g^−1^) | Self-discharge  (Potential retention /Time (hours) | *Ref.* |
| --- | --- | --- | --- | --- | --- | --- | --- | --- | --- | --- | --- | --- | --- | --- |
| Porous carbons | | UC1:5 | No | 1.6 ± 0.2 | 3200 | 2 M ZnSO_4_ | 0.2−1.8 | 326/0.1  79/100 | 145/0.1  35/100 | 117.2  26.2 | 0.08  18.9 | 80%/637000/10 | 67.8%/500 | This work |
|  |  | UC1:50 | No | 1.6 ± 0.2 | 3245 | 2 M ZnSO_4_ | 0.2−1.8 | 446/0.1  52/100 | 198/0.1  23/100 | 161.3  20.8 | 0.08  19.2 | 84%/288000/10 | 67.2%/500 |  |
|  |  | YP-50F | No | 1.6 ± 0.2 | 1783 | 2 M ZnSO_4_ | 0.2−1.8 | 146/0.1  29/30 | 65/0.1  13/30 | 49.7  3.6 | 0.08  8 | 0/50000/10 | 71.1%/500 |  |
|  |  | Activated meso-microporous shell carbon | Graphitic paper | 1 | 3823 | 2 M ZnSO_4_ | 0.2−1.8 | − | 257/0.2  68/20 | 200 | 0.078 | 95%/100000 | − | ^[35]^ |
|  |  | MC800 | No | 1.6 | 3066 | 2 M ZnSO_4_ | 0.2−1.8 | 356/0.1 122/10 | 158/0.1 54/10 | 126.6  43.4 | 0.05  7.44 | 110%/100000/10 | *−* | ^[36]^ |
|  |  | MC600 | No | 1.6 | 1988 | 2 M ZnSO_4_ | 0.2−1.8 | 567/0.1 144/10 | 252/0.1 64/10 | 204.8  51.2 | 0.13  8.78 | 19%/50000/10 | *−* |  |
|  |  | Oxygen-enriched hierarchical porous carbon | Steel mesh | 2 | 2259.5 | 2 M ZnSO_4_ | 0.2–1.8 | 317.8/0.5  202.5/20 | − | 8.73 | 12.5 | 91%/20000/10 | 68.9%/240 | ^[37]^ |
|  |  | PN-HOPC | Stainless steel mesh | 1 | 1702 | 2 M ZnSO_4_ | 0.2−1.8 | − | 211.9/0.2  94.5/80 | 169.5  − | −  64 | 99.3%/60000 | 62.7%/500 | ^[38]^ |
|  |  | O-doped porous carbons | Stainless steel mesh | 0.8 | 1527.7 | 1 M Zn(CF_3_SO_3_)_2_ | 0.2−1.8 | − | 247.6/1  130.6/60  72.2/120 | 178.2  ~52.2 | ~0.3  72.3 | 99.8%/20000/10 | *−* | ^[39]^ |
|  |  | N-doped porous carbon/ | Stainless steel mesh | 1 | 3553.1 | 2 M ZnSO_4_ | 0.2−1.8 | − | 199.1/0.5  118.8/60 | 155.2  45 | 0.2  41.4 | 99.1%/65000/10 | 64.4%/500 | ^[40]^ |
|  |  | Amino functionalized carbon | Carbon paper | 0.4−0.56 | 1568.5 | 1 M ZnCl_2_ | 0−2.0 | − | 255.2/0.3  93.1/10 | 130.9 | 0.154 | 95.5%/50000/10 | *−* | ^[41]^ |
|  |  | Thin-walled porous carbon tile-packed paper | No | ~2.7 | 982 | 1 M ZnSO_4_ | 0.2−1.8 | − | 127.6/0.1  72.4/20 | 92.2  54 | 0.071  15.1 | 90%/10000/5 | *−* | ^[42]^ |
|  |  | Defect-rich carbon | Stainless steel foil | ~1 | 3657.5 | 3 M Zn(CF_3_SO_3_)_2_ | 0.1−1.8 | − | 257/1  72/100 | 168 | 61.7 | 10000/5 | *−* | ^[43]^ |
|  |  | Oxygen-rich porous carbons | Stainless steel mesh | 1.4−2.0 | 1976.6 | 3 M Zn(ClO_4_)_2_ | 0−1.9 | 340.7 | 179.8 | 104.8  40.4 | 0.058  48.8 | 99.2%/30000/20 | 68.4%/240 | ^[44]^ |
|  |  | Highly ordered and compact porous carbon | Stainless steel  mesh | ~2 | 1324 | 2 M ZnSO_4_ | 0.2−1.8 | − | − | 112  81 | 0.1  0.124 | 100%/10000/5 | − | ^[5]^ |
|  |  | N-doped tubular carbons | No | 1−1.5 | 844 | 1 M ZnSO_4_ | 0−2.0 | 341.2/0.1 | 189.5/0.1 | 189.6  100.7 | 0.102  11.26 | 91.5%/50000/ | − | ^[45]^ |
|  |  | P, B-co-doped porous carbons | − | − | − | 2 M ZnSO_4_ | 0.2−1.8 | − | 169.4/0.51816  84.0/10 | 169.4  66.7 | 0.5  20 | 88%/30000/10 | − | ^[46]^ |
|  |  | Hollow bowl-like carbons | Cu foil | 1−1.2 | 791.5 | 2 M ZnSO_4_ | 0.2−1.8 | − | 95.4/0.1  43.6/20 | − | − | 98%/1000/1 | 77.8%/20 | ^[47]^ |
|  |  | Mesoporous hollow carbon sphere | Stainless steel foil | 1−20 | 1275 | 2 M ZnSO_4_ | 0.2−1.8 | − | − | 129.3  36.8 | 0.2664  13.7 | 96%/10000/1 | − | ^[48]^ |
|  |  | Activated carbon | Au | − | 1825 | 2 M ZnSO_4_ | 0.5−1.5 | − | − | 24.9 | 0.78 | −/10000/10 | − | ^[49]^ |
|  |  | Activated graphene | Ti mesh | − | 2957 | 3 M Zn(CF_3_SO_3_)_2_ | 0−1.9 | − | − | 106.3  ~55 | ~0.1  31.4 | 93%/80000/8 | − | ^[50]^ |
|  |  | B,N-co-doped porous carbon | Graphitic paper | ~2.0 | 594 | Gelatin/  1 M ZnSO_4_ | 0.2−1.8 | − | 127.7/0.5  42.8/20 | 86.8  ~17 | ~0.4  12.2 | 81.3%/6500/5 | 77.8%/24 | ^[51]^ |
|  |  | Activated carbon | Stainless steel foil | 0.7−0.8 | 1923 | 2 M ZnSO_4_ | 0.2−1.8 | − | 121/0.1  41/20 | 84  − | 0.069  14.9 | 91%/10000/1 | *−* | ^[52]^ |
|  |  | Composite hydrogel | No | 2.55 | 2630 | 2 M ZnSO_4_ | 0.3−1.6 | − | 154/0.1 | 205  138 | 0.458  2.455 | 80.5%/6000/5 | *−* | ^[53]^ |
|  |  | Bio-carbon-derived porous material | Carbon coated Al foil | − | 3554 | 0.1 M ZnSO_4_ | 0−1.7 | 170/0.1 | 80.3/0.180.392 | 52.7 | 1.725 | 91%/20000/2 | *−* | ^[54]^ |
| MXene |  | Ti_3_C_2_ | − | 2.8 | − | 0.1 M ZnSO_4_ | 0.01−1.0 | 92/0.02 | 25.3/0.1 | − | − | 96%/1000/  0.2 | − | ^[55]^ |
|  |  | Sn^4+^-Ti_2_CT_x_/Carbon cloth | Carbon cloth | − | 6.4 | 23.75 M LiTFSI +  1 M Zn(CF_3_SO_3_)_2_ | 0.1−2.0 | − | 138/0.1  92/5.0 | − | − | 95%/12500/  0.5 | 75.5%/  548 h | ^[56]^ |
|  |  | MXene/nanocellulose | No | − | − | ZnSO_4_ | 0−1.3 | − | 92.1/0.5 mA cm^−2^ | − | − | 94%/10000/8 mA cm^−2^ | *−* | ^[57]^ |
| Metal oxide |  | RuO_2_·H_2_O | Stainless steel foil | 2.5-3.0 | 57 | 2 M Zn(CF_3_SO_3_)_2_ /2 M ZnSO_4_ | 0.4−1.6 | − | 122/0.1  98/20 | 82 | 16.74 | 87.5%/10000/20 | − | ^[58]^ |
|  |  | Zn-doped δ-MnO_­2_ | Activated carbon cloth | 1.6-2.4 | 153.78 | 2 M ZnSO_4_ | 0−2 | 282.9/0.2 | 157.2/0.2157 | 157.2 | 16 | 80.2%/30000/2 | − | ^[59]^ |
| Others |  | Few-layer phosphorene | Carbon cloth | 0.6-0.8 | − | 21 M LiTFSI  1 M Zn(CF_3_SO_3_)_2_ | 0−2.2 | 304/0.2  145.9/6.4 | − | 204.4  ~40 | ~0.9  18.79 | 5000 | 76.2%/  300 h | ^[60]^ |
|  |  | TiN | Carbon cloth | 0.8 | − | ZnSO_4_ | 0.2−1.8 | 489.8 | 314.5 | 135  56 | 0.186  3.472 | 10000/1 | 83.92%/  500 h | ^[61]^ |

**Table S5.** Comparison of self-discharge in supercapacitors using carbon-based electrode materials and non-Zn-based electrolytes.

| Electrodes | Electrolytes | Separators | Range of voltage (V) | Time (h) | Voltage retention | *Ref.* |
| --- | --- | --- | --- | --- | --- | --- |
| UC1:5 | 2 M ZnSO_4_/H_2_O | Glass fiber | 1.8 🡪 1.22 | 500 | 67.8% | This work |
| UC1:50 | 2 M ZnSO_4_/H_2_O | Glass fiber | 1.8 🡪 1.21 | 500 | 67.2% |  |
| YP-50F | 2 M ZnSO_4_/H_2_O | Glass fiber | 1.8 🡪 1.28 | 500 | 71.1% |  |
| Activated carbon | 2 M KOH/H_2_O | PAN@SDBS | 1.0 🡪 0.58 | 3.3 | 58% | ^[62]^ |
| Activated carbon | 1 M H_2_SO_4_/H_2_O | Modifying cellulose membrane with PEI | 0.8 🡪 0.3 | 10 | 37.5% | ^[63]^ |
| Graphene/polyaniline | PVA-H_2_SO_4_ gel/H_2_O | / | 0.8 🡪 0.53 | 4 | 66.2% | ^[64]^ |
| Activated carbon | PVA-Li_2_SO_4_ gel/H_2_O | / | 1.8 🡪 0.8 | 5 | 44.4% | ^[65]^ |
| Carbon | Organic electrolyte  1 M SBP-BF_4_/PC | Cellulose | 2.7 🡪 2.3 | 10 | 85% | ^[66]^ |
| Activated carbon | 2% of 5CB in TEMABF_4_/ACN | Cellulose | 2 🡪 1.5 | 22 | 75% | ^[67]^ |
| Activated carbon | TEMABF_4_/ACN | Cellulose | 2 🡪 1 | 22 | 50% | ^[67]^ |
| Activated carbon | Bentonite clay@BMIMBF_4_  based electrolyte | / | 3 🡪 2.1 | 60 | 61.1% | ^[68]^ |
| Activated carbon | EMIMBF_4_ | Cellulose | 3 🡪 1.8 | 12 | 40% | ^[68]^ |
| Graphene | EMIMBF_4_ | Glass fiber | 3 🡪 0.5 | 83 | 16.7% | ^[69]^ |

**References**

[1] D. B. Schuepfer, F. Badaczewski, J. M. Guerra-Castro, D. M. Hofmann, C. Heiliger, B. Smarsly, P. J. Klar, Assessing the structural properties of graphitic and non-graphitic carbons by Raman spectroscopy. *Carbon* **2020**, *161*, 359.

[2] T. Stanimirova, R. Nikolova, N. Petrova, Crystal structure of new zinc-hydroxy-sulfate-hydrate Zn₄(OH)₆SO₄·2–2.25H₂O. *Crystals* **2024**, *14*, 183.

[3] L. Liu, Y. C. Wu, L. Huang, K. Liu, B. Duployer, P. Rozier, P. L. Taberna, P. Simon, Alkali ions pre-intercalated layered MnO2 nanosheet for zinc-ions storage. *Adv. Energy Mater.* **2021**, *11*, 2101287.

[4] E. Zhang, Y. C. Wu, H. Shao, V. Klimavicius, H. Zhang, P. L. Taberna, J. Grothe, G. Buntkowsky, F. Xu, P. Simon, S. Kaskel, Unraveling the capacitive charge storage mechanism of nitrogen-doped porous carbons by EQCM and ssNMR. *J. Am. Chem. Soc.* **2022**, *144*, 14217.

[5] H. Ma, H. Chen, M. Wu, F. Chi, F. Liu, J. Bai, H. Cheng, C. Li, L. Qu, Maximization of spatial charge density: an approach to ultrahigh energy density of capacitive charge storage. *Angew. Chem. Int. Ed.* **2020**, *59*, 14541.

[6] F. Herziger, R. Mirzayev, E. Poliani, J. Maultzsch, In-situ Raman study of laser-induced graphene oxidation. *Phys. Status Solidi B* **2015**, *252*, 2451.

[7] J. Li, J. Kossmann, K. Zeng, K. Zhang, B. Wang, M. Antonietti, M. Odziomek, N. López-Salas, When high-temperature cesium chemistry meets self-templating: metal acetates as building blocks of unusual highly porous carbons. *Angew. Chem. Int. Ed.* **2023**, *62*, e202217808.

[8] J. Kossmann, D. Piankova, N. V. Tarakina, J. Heske, T. D. Kühne, J. Schmidt, M. Antonietti, N. López-Salas, Guanine condensates as covalent materials and the concept of cryptopores. *Carbon* **2021**, *172*, 497.

[9] W. Cai, J. Ding, Y. He, X. Chen, D. Yuan, C. Chen, L. Cheng, W. Du, H. Wan, G. Guan, Nitrogen-doped microporous carbon prepared by one-step carbonization: rational design of a polymer precursor for efficient CO₂ capture. *Energy Fuels* **2021**, *35*, 8857.

[10] P. Zhang, Y. Zhong, J. Ding, J. Wang, M. Xu, Q. Deng, Z. Zeng, S. Deng, A new choice of polymer precursor for solvent-free method: Preparation of N-enriched porous carbons for highly selective CO₂ capture. *Chem. Eng. J.* **2019**, *355*, 963.

[11] A. C. Dassanayake, M. Jaroniec, Dual optimization of microporosity in carbon spheres for CO2 adsorption by using pyrrole as the carbon precursor and potassium salt as the activator. *J. Mater. Chem. A* **2017**, *5*, 19456.

[12] C. Chen, H. Huang, Y. Yu, J. Shi, C. He, R. Albilali, H. Pan, Template-free synthesis of hierarchical porous carbon with controlled morphology for CO₂ efficient capture. *Chem. Eng. J.* **2018**, *353*, 584.

[13] G. Singh, S. Tiburcius, S. M. Ruban, D. Shanbhag, C. I. Sathish, K. Ramadass, A. Vinu, Pure and strontium carbonate nanoparticles functionalized microporous carbons with high specific surface areas derived from chitosan for CO₂ adsorption. *Emergent Mater.* **2019**, *2*, 337.

[14] B. Liu, S. Yao, X. Liu, X. Li, R. Krishna, G. Li, Q. Huo, Y. Liu, Two analogous polyhedron-based MOFs with high density of Lewis basic sites and open metal sites: significant CO₂ capture and gas selectivity performance. *ACS Appl. Mater. Interfaces* **2017**, *9*, 32820.

[15] C. Wang, L. Li, J. G. Bell, X. Lv, S. Tang, X. Zhao, K. M. Thomas, Hysteretic gas and vapor sorption in flexible interpenetrated lanthanide-based metal-organic frameworks with coordinated molecular gating via reversible single-crystal-to-single-crystal transformation for enhanced selectivity. *Chem. Mater.* **2015**, *27*, 1502.

[16] L. Lei, Y. Cheng, C. Chen, M. Kosari, Z. Jiang, C. He, Taming structure and modulating carbon dioxide (CO₂) adsorption isosteric heat of nickel-based metal organic framework (MOF-74(Ni)) for remarkable CO₂ capture. *J. Colloid Interface Sci.* **2022**, *612*, 132.

[17] L. Yang, X. Cui, Y. Zhang, Q. Wang, Z. Zhang, X. Suo, H. Xing, Anion pillared metal–organic framework embedded with molecular rotors for size-selective capture of CO₂ from CH₄ and N₂. *ACS Sustain. Chem. Eng.* **2019**, *7*, 3138.

[18] R. Kodasma, J. Fermoso, A. Sanna, Li–LSX–zeolite evaluation for post-combustion CO₂ capture. *Chem. Eng. J.* **2019**, *358*, 1351.

[19] S. K. Das, X. Wang, Z. Lai, Facile synthesis of triazine-triphenylamine-based microporous covalent polymer adsorbent for flue gas CO₂ capture. *Micropor. Mesopor. Mater.* **2018**, *255*, 76.

[20] S. K. Das, X. Wang, M. M. Ostwal, Y. Zhao, Y. Han, Z. Lai, Highly stable porous covalent triazine–piperazine linked nanoflower as a feasible adsorbent for flue gas CO₂ capture. *Chem. Eng. Sci.* **2016**, *145*, 21.

[21] H. Ma, H. Ren, X. Zou, S. Meng, F. Sun, G. Zhu, Post-metalation of porous aromatic frameworks for highly efficient carbon capture from CO₂ + N₂ and CH₄ + N₂ mixtures. *Polym. Chem.* **2014**, *5*, 144.

[22] J. Kossmann, R. Rothe, T. Heil, M. Antonietti, N. López-Salas, Ultrahigh water sorption on highly nitrogen doped carbonaceous materials derived from uric acid. *J. Colloid Interface Sci.* **2021**, *602*, 880.

[23] R. Walczak, B. Kurpil, A. Savateev, T. Heil, J. Schmidt, Q. Qin, M. Antonietti, M. Oschatz, Template- and metal-free synthesis of nitrogen-rich nanoporous “noble” carbon materials by direct pyrolysis of a preorganized hexaazatriphenylene precursor. *Angew. Chem. Int. Ed.* **2018**, *57*, 10765.

[24] L. Huber, S. B. Hauser, E. Brendlé, P. Ruch, J. Ammann, R. Hauert, R. N. Widmer, C. J. Ubert, S. K. Matam, S. Yoon, Y. Zhang, M. M. Koebel, The effect of activation time on water sorption behavior of nitrogen-doped, physically activated, monolithic carbon for adsorption cooling. *Micropor. Mesopor. Mater.* **2019**, *276*, 239.

[25] S. Gadipelli, C. A. Howard, J. Guo, N. T. Skipper, H. Zhang, P. R. Shearing, D. J. L. Brett, Superior multifunctional activity of nanoporous carbons with widely tunable porosity: enhanced storage capacities for carbon-dioxide, hydrogen, water, and electric charge. *Adv. Energy Mater.* **2020**, *10*, 1903649.

[26] M. Thommes, J. Morell, K. A. Cychosz, M. Fröba, Combining nitrogen, argon, and water adsorption for advanced characterization of ordered mesoporous carbons (CMKs) and periodic mesoporous organosilicas (PMOs). *Langmuir* **2013**, *29*, 14893.

[27] S. M. Towsif Abtab, D. Alezi, P. M. Bhatt, A. Shkurenko, Y. Belmabkhout, H. Aggarwal, Ł. J. Weseliński, N. Alsadun, U. Samin, M. N. Hedhili, M. Eddaoudi, Reticular chemistry in action: a hydrolytically stable MOF capturing twice its weight in adsorbed water. *Chem* **2018**, *4*, 94.

[28] J. Ehrenmann, S. K. Henninger, C. Janiak, Water adsorption characteristics of MIL-101 for heat-transformation applications of MOFs. *Eur. J. Inorg. Chem.* **2011**, 471.

[29] G. E. Cmarik, M. Kim, S. M. Cohen, K. S. Walton, Tuning the adsorption properties of UIO-66 via ligand functionalization. *Langmuir* **2012**, *28*, 15606.

[30] A. Cadiau, J. S. Lee, D. Damasceno Borges, P. Fabry, T. Devic, M. T. Wharmby, C. Martineau, D. Foucher, F. Taulelle, C. H. Jun, Y. K. Hwang, N. Stock, M. F. De Lange, F. Kapteijn, J. Gascon, G. Maurin, J. S. Chang, C. Serre, Design of hydrophilic metal organic framework water adsorbents for heat reallocation. *Adv. Mater.* **2015**, *27*, 4775.

[31] A. J. Rieth, S. Yang, E. N. Wang, M. Dincǎ, Record atmospheric fresh water capture and heat transfer with a material operating at the water uptake reversibility limit. *ACS Cent. Sci.* **2017**, *3*, 668.

[32] S. Hug, L. Stegbauer, H. Oh, M. Hirscher, B. V. Lotsch, Nitrogen-rich covalent triazine frameworks as high-performance platforms for selective carbon capture and storage. *Chem. Mater.* **2015**, *27*, 8001.

[33] B. P. Biswal, S. Kandambeth, S. Chandra, D. B. Shinde, S. Bera, S. Karak, B. Garai, U. K. Kharul, R. Banerjee, Pore surface engineering in porous, chemically stable covalent organic frameworks for water adsorption. *J. Mater. Chem. A* **2015**, *3*, 23664.

[34] H. L. Nguyen, N. Hanikel, S. J. Lyle, C. Zhu, D. M. Proserpio, O. M. Yaghi, A porous covalent organic framework with voided square grid topology for atmospheric water harvesting. *J. Am. Chem. Soc.* **2020**, *142*, 2218.

[35] X. Li, C. Cai, P. Hu, B. Zhang, P. Wu, H. Fan, Z. Chen, L. Zhou, L. Mai, H. J. Fan, Gradient pores enhance charge storage density of carbonaceous cathodes for Zn-ion capacitor. *Adv. Mater.* **2024**, *36*, 2400184.

[36] J. Li, Y. Xu, P. Li, A. Völkel, I. Saldaña, M. Antonietti, N. López-Salas, M. Odziomek, Beyond conventional carbon activation: creating porosity without etching using cesium effect. *Adv. Mater.* **2024**, *36*, 2311655.

[37] H. Li, P. Su, Q. Liao, Y. Liu, Y. Li, X. Niu, X. Liu, K. Wang, Olive leaves-derived hierarchical porous carbon as cathode material for anti-self-discharge zinc-ion hybrid capacitor. *Small* **2023**, *19*, 2304172.

[38] Z. Peng, A. G. Bannov, S. Li, Y. Huang, L. Tang, L. Tan, Y. Chen, Coupling uniform pore size and multi chemisorption sites: hierarchically ordered porous carbon for ultra-fast and large zinc ion storage. *Adv. Funct. Mater.* **2023**, *33*, 2303205.

[39] L. Wang, M. Peng, J. Chen, X. Tang, L. Li, T. Hu, K. Yuan, Y. Chen, High energy and power zinc ion capacitors: a dual-ion adsorption and reversible chemical adsorption coupling mechanism. *ACS Nano* **2022**, *16*, 2877.

[40] L. Wang, M. Peng, J. Chen, T. Hu, K. Yuan, Y. Chen, Eliminating the micropore confinement effect of carbonaceous electrodes for promoting Zn-ion storage capability. *Adv. Mater.* **2022**, *34*, 2203744.

[41] X. Shi, J. Xie, F. Yang, F. Wang, D. Zheng, X. Cao, Y. Yu, Q. Liu, X. Lu, Compacting electric double layer enables carbon electrode with ultrahigh Zn ion storage capability. *Angew. Chem. Int. Ed.* **2022**, *61*, e202214773.

[42] Y. Cao, X. Tang, M. Liu, Y. Zhang, T. Yang, Z. Yang, Y. Yu, Y. Li, J. Di, Q. Li, Thin-walled porous carbon tile-packed paper for high-rate Zn-ion capacitor cathode. *Chem. Eng. J.* **2022**, *431*, 133241.

[43] W. Fan, J. Ding, J. Ding, Y. Zheng, W. Song, J. Lin, C. Xiao, C. Zhong, H. Wang, W. Hu, Identifying heteroatomic and defective sites in carbon with dual-ion adsorption capability for high energy and power zinc ion capacitor. *Nano-Micro Lett.* **2021**, *13*, 59.

[44] J. Yin, W. Zhang, W. Wang, N. A. Alhebshi, N. Salah, H. N. Alshareef, Electrochemical zinc ion capacitors enhanced by redox reactions of porous carbon cathodes. *Adv. Energy Mater.* **2020**, *10*, 2001705.

[45] R. Yuksel, O. Buyukcakir, P. K. Panda, S. H. Lee, Y. Jiang, D. Singh, S. Hansen, R. Adelung, Y. K. Mishra, R. Ahuja, R. S. Ruoff, Necklace-like nitrogen-doped tubular carbon 3D frameworks for electrochemical energy storage. *Adv. Funct. Mater.* **2020**, *30*, 1909725.

[46] Y. G. Lee, G. H. An, Synergistic effects of phosphorus and boron co-incorporated activated carbon for ultrafast zinc-ion hybrid supercapacitors. *ACS Appl. Mater. Interfaces* **2020**, *12*, 41342.

[47] R. Fei, H. Wang, Q. Wang, R. Qiu, S. Tang, R. Wang, B. He, Y. Gong, H. J. Fan, In situ hard-template synthesis of hollow bowl-like carbon: a potential versatile platform for sodium and zinc ion capacitors. *Adv. Energy Mater.* **2020**, *10*, 2002741.

[48] P. Liu, W. Liu, Y. Huang, P. Li, J. Yan, K. Liu, Mesoporous hollow carbon spheres boosted, integrated high performance aqueous Zn-Ion energy storage. *Energy Storage Mater.* **2020**, *25*, 858.

[49] P. Zhang, Y. Li, G. Wang, F. Wang, S. Yang, F. Zhu, X. Zhuang, O. G. Schmidt, X. Feng, Zn-Ion hybrid micro-supercapacitors with ultrahigh areal energy density and long-term durability. *Adv. Mater.* **2019**, *31*, 1806005.

[50] S. Wu, Y. Chen, T. Jiao, J. Zhou, J. Cheng, B. Liu, S. Yang, K. Zhang, W. Zhang, An aqueous zn-ion hybrid supercapacitor with high energy density and ultrastability up to 80000 cycles. *Adv. Energy Mater.* **2019**, *9*, 1902915.

[51] Y. Lu, Z. Li, Z. Bai, H. Mi, C. Ji, H. Pang, C. Yu, J. Qiu, High energy-power Zn-ion hybrid supercapacitors enabled by layered B/N co-doped carbon cathode. *Nano Energy* **2019**, *66*, 104132.

[52] L. Dong, X. Ma, Y. Li, L. Zhao, W. Liu, J. Cheng, C. Xu, B. Li, Q. H. Yang, F. Kang, Extremely safe, high-rate and ultralong-life zinc-ion hybrid supercapacitors. *Energy Storage Mater.* **2018**, *13*, 96.

[53] J. Han, K. Wang, W. Liu, C. Li, X. Sun, X. Zhang, Y. An, S. Yi, Y. Ma, Rational design of nano-architecture composite hydrogel electrode towards high performance Zn-ion hybrid cell. *Nanoscale* **2018**, *10*, 13083.

[54] H. Wang, M. Wang, Y. Tang, A novel zinc-ion hybrid supercapacitor for long-life and low-cost energy storage applications. *Energy Storage Mater.* **2018**, *13*, 1.

[55] P. A. Maughan, N. Tapia-Ruiz, N. Bimbo, In-situ pillared MXene as a viable zinc-ion hybrid capacitor. *Electrochim. Acta* **2020**, *341*, 136061.

[56] X. Li, M. Li, Q. Yang, D. Wang, L. Ma, G. Liang, Z. Huang, B. Dong, Q. Huang, C. Zhi, Vertically aligned Sn4+ preintercalated Ti2CTX MXene sphere with enhanced Zn ion transportation and superior cycle lifespan. *Adv. Energy Mater.* **2020**, *10*, 2001394.

[57] J. Chen, H. Chen, M. Chen, W. Zhou, Q. Tian, C. P. Wong, Nacre-inspired surface-engineered MXene/nanocellulose composite film for high-performance supercapacitors and zinc-ion capacitors. *Chem. Eng. J.* **2022**, *428*, 131380.

[58] L. Dong, W. Yang, W. Yang, C. Wang, Y. Li, C. Xu, S. Wan, F. He, F. Kang, G. Wang, High‑power and ultralong‑life aqueous zinc‑ion hybrid capacitors based on pseudocapacitive charge storage. *Nano-Micro Lett.* **2019**, *11*, 94.

[59] S. He, Z. Mo, C. Shuai, W. Liu, R. Yue, G. Liu, H. Pei, Y. Chen, N. Liu, R. Guo, Pre-intercalation δ-MnO2 zinc-ion hybrid supercapacitor with high energy storage and ultra-long cycle life. *Appl. Surf. Sci.* **2022**, *577*, 151904.

[60] Z. Huang, A. Chen, F. Mo, G. Liang, X. Li, Q. Yang, Y. Guo, Z. Chen, Q. Li, B. Dong, C. Zhi, Phosphorene as cathode material for high-voltage, anti-self-discharge zinc ion hybrid capacitors. *Adv. Energy Mater.* **2020**, *10*, 2001024.

[61] Z. Huang, T. Wang, H. Song, X. Li, G. Liang, D. Wang, Q. Yang, Z. Chen, L. Ma, Z. Liu, B. Gao, J. Fan, C. Zhi, Effects of anion carriers on capacitance and self‐discharge behaviors of zinc Ion capacitors. *Angew. Chem. Int. Ed.* **2021**, *60*, 1024.

[62] H. Peng, L. Xiao, K. Sun, G. Ma, G. Wei, Z. Lei, Preparation of a cheap and environmentally friendly separator by coaxial electrospinning toward suppressing self-discharge of supercapacitors. *J. Power Sources* **2019**, *435*, 226800.

[63] H. Wang, Q. Zhou, B. Yao, H. Ma, M. Zhang, C. Li, G. Shi, Suppressing the self-discharge of supercapacitors by modifying separators with an ionic polyelectrolyte. *Adv. Mater. Interfaces* **2018**, *5*, 1701547.

[64] K. Li, J. Liu, Y. Huang, F. Bu, Y. Xu, Integration of ultrathin graphene/polyaniline composite nanosheets with a robust 3D graphene framework for highly flexible all-solid-state supercapacitors with superior energy density and exceptional cycling stability. *J. Mater. Chem. A* **2017**, *5*, 5466.

[65] L. Q. Fan, Q. M. Tu, C. L. Geng, J. L. Huang, Y. Gu, J. M. Lin, Y. F. Huang, J. H. Wu, High energy density and low self-discharge of a quasi-solid-state supercapacitor with carbon nanotubes incorporated redox-active ionic liquid-based gel polymer electrolyte. *Electrochim. Acta* **2020**, *331*, 135425.

[66] S. Yuan, X. Huang, H. Wang, L. Xie, J. Cheng, Q. Kong, G. Sun, C. M. Chen, Structure evolution of oxygen removal from porous carbon for optimizing supercapacitor performance. *J. Energy Chem.* **2020**, *51*, 396.

[67] M. Xia, J. Nie, Z. Zhang, X. Lu, Z. L. Wang, Suppressing self-discharge of supercapacitors via electrorheological effect of liquid crystals. *Nano Energy* **2018**, *47*, 43.

[68] Z. Wang, X. Chu, Z. Xu, H. Su, C. Yan, F. Liu, B. Gu, H. Huang, D. Xiong, H. Zhang, W. Deng, H. Zhang, W. Yang, Extremely low self-discharge solid-state supercapacitors: Via the confinement effect of ion transfer. *J. Mater. Chem. A* **2019**, *7*, 8633.

[69] J. Wang, B. Ding, X. Hao, Y. Xu, Y. Wang, L. Shen, H. Dou, X. Zhang, A modified molten-salt method to prepare graphene electrode with high capacitance and low self-discharge rate. *Carbon* **2016**, *102*, 255.
